# An HHEX/IKKα positive feedback loop promotes intestinal inflammation

**DOI:** 10.1172/JCI192074

**Published:** 2026-03-17

**Authors:** Zhebin Hua, Weimin Xu, Wenjun Ding, Zhuoyue Fu, Yaosheng Wang, Yiqing Yang, Fangyuan Liu, Zhujiang Dai, Wenbo Tang, Weijun Ou, Wensong Ge, YingWei Chen, Zhongchuan Wang, Chen-Ying Liu, Peng Du

**Affiliations:** 1 Department of Colorectal Surgery, Xinhua Hospital, Shanghai Jiaotong University School of Medicine, Shanghai, China.; 2 Shanghai Colorectal Cancer Research Center, Shanghai, China.; 3 Department of Gastroenterology, Xinhua Hospital, Shanghai Jiaotong University School of Medicine, Shanghai, China.

**Keywords:** Gastroenterology, Immunology, Inflammation, Inflammatory bowel disease, NF-kappaB

## Abstract

The dynamic assembly and regulation of the IκB kinase (IKK) complex in the NF-κB pathway are central to the pathogenesis and progression of inflammatory bowel disease (IBD). We recently reported that the transcription factor hematopoietically expressed homeobox (HHEX) promotes colitis-associated colorectal cancer, but the potential role of HHEX in intestinal inflammation remains uncharacterized. Here, we found that HHEX is upregulated in inflamed colons in a colitis mouse model and in clinical IBD samples. HHEX overexpression increased inflammatory cytokine expression, and HHEX loss largely abrogated the inflammatory response in vitro and intestinal inflammation in vivo. Mechanistically, IKKα phosphorylates HHEX at S213 to stabilize HHEX in response to TNF-α by inhibiting the interaction of HHEX with the E3 ubiquitin ligase MID2 and subsequent K48-linked ubiquitination and protein degradation. Importantly, HHEX interacted with and stabilized the IKKα/IKKβ complex via its N-terminal domain, thereby activating the NF-κB pathway and establishing a positive feedback loop that exacerbates intestinal inflammation. Our study reveals a transcription-independent function of HHEX in promoting IKK complex assembly and colitis, identifying HHEX as an IBD susceptibility gene and a potential target for IBD treatment.

## Abstract 2

HHEX as a positive regulator of the IKK complex that activates the NF-κB pathway. Inflammatory stimuli can stabilize HHEX by IKKα-dependent phosphorylation.

## Graphical abstract

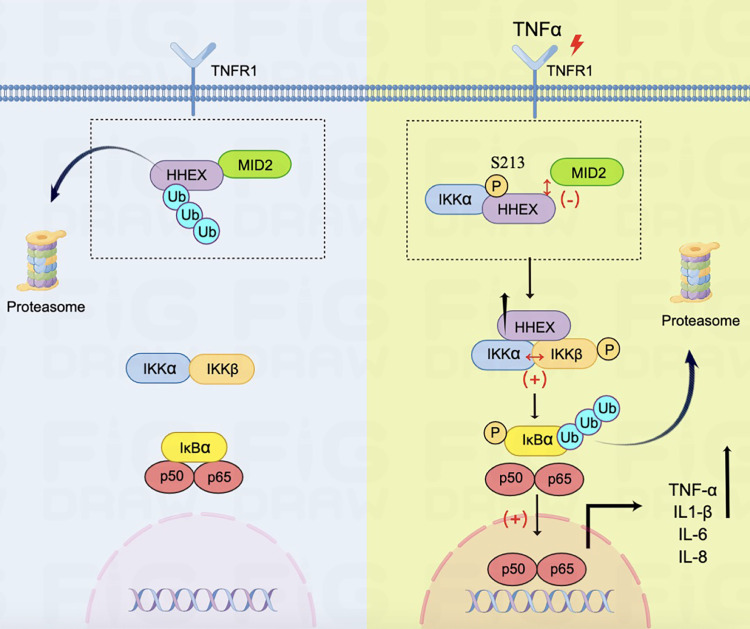

## Introduction

Inflammatory bowel disease (IBD), including Crohn’s disease (CD) and ulcerative colitis (UC), is a chronic, nonspecific intestinal inflammatory disorder characterized by long-term mucosal injury and pathological inflammation (1, 2). Approximately 70% of patients with CD and 25% of those with UC eventually require surgical intervention due to either failure of biologic therapies, such as infliximab, or the onset of severe complications (3–5). Thus, the etiology and pathology of IBD are complicated, and identification of new drug targets for IBD treatment is urgently needed. Dysregulation of multiple inflammatory signaling pathways contributes to the development and progression of IBD (6, 7). NF-κB is one of the key signaling pathways involved in the activated inflammatory response in IBD (8, 9). In the canonical NF-κB pathway, signal-induced phosphorylation of IκB by the IκB kinase (IKK) complex is the central event that triggers the activation of NF-κB transcription factors, the subsequent induction of transcription, and the production of various pro-inflammatory cytokines, such as TNF-α, IL-6, and IL-8. Inhibiting IKKs is a promising therapeutic strategy for targeting the NF-κB pathway to treat IBD. However, the broad tissue distribution and critical biological functions of the IKK complex pose concerns regarding the potential side effects and safety concerns of systematic IKK inhibition (10, 11). In addition to the regulatory subunit IKKγ/NEMO, 2 catalytic subunits, IKKα/IKK1 and IKKβ/IKK2, are dynamically modulated by multiple interacting proteins and posttranslational modifications in the pathological inflammatory context. Thus, characterizing the dynamic assembly and regulation of the IKK complex during the development and progression of IBD could lead to new strategies for targeting the NF-κB pathway.

Hematopoietically expressed homeobox (HHEX), also called proline-rich homeodomain (PRH), is a transcription factor with a DNA-binding homeobox (12). HHEX can act as either a transcriptional repressor or activator depending on the cell context and interacting transcriptional cofactors (13, 14). In addition to its critical role in hematopoiesis and the development of the vasculature, liver, and pancreas, dysregulation of HHEX is implicated in several diseases, including leukemia, solid tumors, and type 2 diabetes (T2D) (15–19). A GWAS revealed that the rs7911264 SNP near the HHEX gene locus is associated with susceptibility to IBD (20, 21). Recently, HHEX was identified as a hub gene between T2D and IBD by integrated Mendelian randomization and bioinformatic analysis, further indicating the potential role of HHEX in IBD (22). In addition, our previous study revealed that the overexpression of HHEX in colorectal cancer (CRC) promotes tumorigenesis by interacting with and activating the oncogenic YAP-TEAD4 complex, the main downstream transcriptional effector of the Hippo pathway (23). However, the biological function and dysregulated mechanisms of HHEX during intestinal inflammation remain largely uncharacterized.

In this study, we report that HHEX is a positive regulator of the IKK complex in intestinal epithelial cells (IECs). HHEX interacts with and promotes the formation of the IKK complex, the subsequent activation of NF-κB, and the induction of pro-inflammatory genes. In response to TNF-α, IKKα kinase phosphorylates HHEX at S213, which disrupts its interaction with the E3 ubiquitin ligase MID2 and inhibits the K48-linked ubiquitination and proteasome-mediated degradation of HHEX, thus leading to the stabilization of HHEX under inflammatory stimuli. HHEX protein levels are increased in inflamed colon tissues from patients with IBD. Therefore, our study revealed a positive feedback loop of the IKK complex modulated by HHEX, highlighting the pro-inflammatory function of HHEX and potential therapeutic strategies for targeting the HHEX/IKK complex in IBD.

## Results

### HHEX expression is increased in inflamed colon tissues of patients with UC.

**Figure 1:**
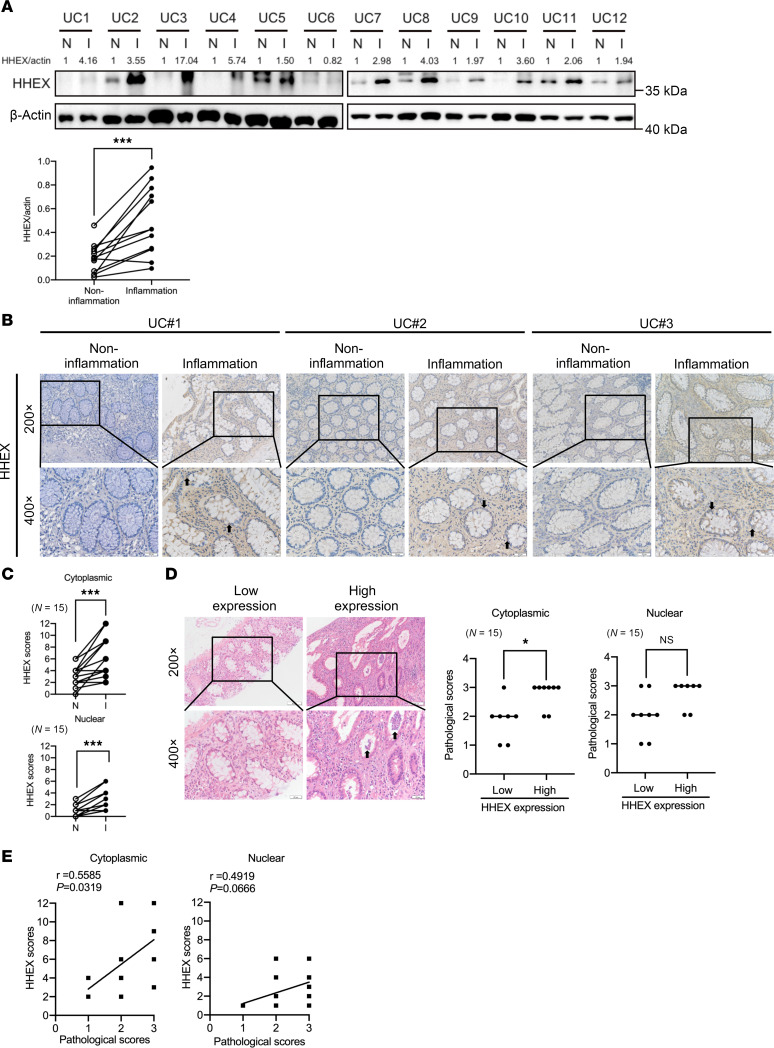
HHEX expression is increased in inflamed colon tissues of patients with UC. (**A**) Western blot analysis of HHEX expression in 12 pairs of inflamed and paired adjacent noninflamed colon tissues from patients with UC. (**B** and **C**) IHC was used to examine, evaluate, and score the expression level of HHEX in 15 pairs of inflamed and paired adjacent noninflamed colon tissues from patients with UC. (Arrows indicate nuclei with high HHEX expression, magnification: 200×, upper panels; 400×, lower panels.) (**D**) Hematoxylin-eosin staining images of a representative pair of inflamed colon tissues with low and high levels of cytoplasmic/nuclear HHEX expression and pathological scores of 15 pairs of patients (figure shows crypt atrophy, irregularity, and associated ulceration; arrows indicate crypt abscesses, magnification: 200×, upper panels; 400×, lower panels). (**E**) Spearman’s correlation analysis was used to evaluate the correlation between the pathological scores and IHC scores of cytoplasmic/nuclear HHEX in 15 pairs of inflamed colon tissues from patients with UC. Two-tailed paired Student’s *t* test (**A**), Wilcoxon’s signed-rank test (**C**), and Mann-Whitney *U* test (**D**) were performed to assess statistical significance. * *P* < 0.05, *** *P* < 0.001.

To explore the potential biological function of HHEX in intestinal inflammation, we first determined whether HHEX is dysregulated in the intestinal mucosa of patients with IBD by conducting Western blot analysis on inflamed and paired adjacent noninflamed mucosal tissues from 12 patients with UC. We observed a slight increase in HHEX protein levels (fold-change [FC] < 3) in inflamed tissues from 5 patients, whereas 6 patients presented a moderate to significant increase (FC > 3) in inflamed tissues (Figure 1A). Semiquantitative analysis also showed that HHEX protein levels were statistically upregulated in inflamed tissues (Figure 1A). Immunohistochemistry (IHC) confirmed the elevated HHEX expression in inflamed UC tissues, and HHEX was highly expressed in both cytoplasm and nucleus (Figure 1B). Specifically, HHEX expression was upregulated in both the cytoplasm and nucleus of IECs (Figure 1C). The pathological scores of inflamed tissues with high cytoplasmic HHEX expression in IECs were significantly greater than those of inflamed tissues with low cytoplasmic HHEX expression (Figure 1D). A similar, though less significant, trend was observed between pathological scores and nuclear HHEX expression (Figure 1D). Additionally, a positive correlation was observed between cytoplasmic HHEX expression in IECs and pathological score across 15 UC samples (*R* = 0.5585, *P* = 0.0319), whereas correlation between nuclear HHEX expression in IECs and pathological score did not reach statistical significance (*R* = 0.4919, *P* = 0.0666) (Figure 1E). Taken together, these results indicate that HHEX is highly expressed in inflamed intestines and that the upregulation of HHEX could play a role in the development of intestinal inflammation.

### HHEX promotes the TNF-α–induced inflammatory response in IECs.

**Figure 2:**
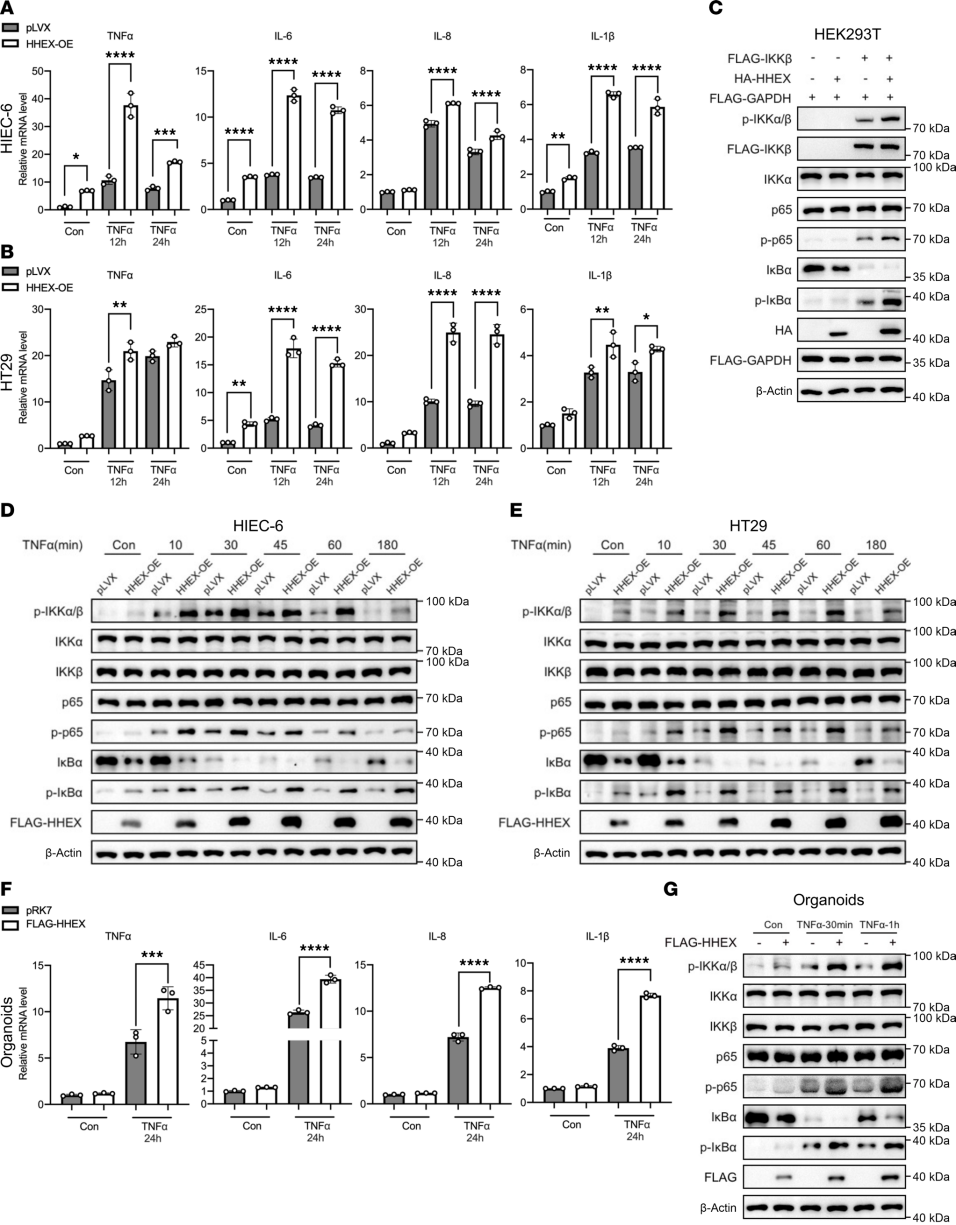
HHEX promotes the TNF-α–induced inflammatory response in human IECs. (**A** and **B**) The mRNA levels of pro-inflammatory cytokines in HHEX overexpression and control HIEC-6 (**A**) or HT29 (**B**) cells were measured via qRT-PCR. The cells were treated with TNF-α (10 ng/mL) for the indicated times before qRT-PCR analysis. (**C**) Overexpression of HHEX activated the NF-κB pathway in HEK293T cells. (**D** and **E**) Western blot analysis of the activation status of the NF-κB pathway in the control and FLAG-HHEX–overexpressing HIEC-6 (**D**) or HT29 (**E**) cells following short-term TNF-α stimulation. (**F**) The mRNA levels of pro-inflammatory cytokines in the HHEX-overexpressing and control colonic organoids were measured via qRT-PCR. The cells were treated with TNF-α (10 ng/mL) for the indicated times before qRT-PCR analysis. (**G**) Western blot analysis of the activation status of the NF-κB pathway in the control and FLAG-HHEX–overexpressing colonic organoids following short-term TNF-α stimulation. The data are presented as the means ± SDs and represent 3 independent experiments in this figure. One-way ANOVA with Tukey’s multiple-comparison test (**A**, **B**, and **F**) was performed to assess statistical significance. * *P* < 0.05, ** *P* < 0.01, *** *P* < 0.001, **** *P* < 0.0001. “pLVX” indicates the pLVX vector control for HHEX overexpression.

To investigate the biological role of HHEX in the inflammatory response, we established HIEC-6 and HT29 cell lines with stable HHEX knockdown or overexpression (Supplemental Figure 1, A–D; supplemental material available online with this article; https://doi.org/10.1172/JCI192074DS1). Cells treated with TNF-α presented increased transcription of inflammatory cytokines, including TNF-α, IL-1β, IL-6, and IL-8, which was largely abrogated by HHEX knockdown in both HIEC-6 and HT29 cells (Supplemental Figure 1, E and F). Conversely, HHEX overexpression further increased the mRNA levels of these inflammatory cytokine genes upon TNF-α stimulation (Figure 2, A and B). Next, we explored the effect of HHEX on the canonical NF-κB pathway activated by TNF-α. Transient overexpression of IKKβ led to activation of the IKK kinase complex, as indicated by the increased phosphorylation levels of IKKα/β and IKK substrates (p-IκBα, p-p65) as well as the decreased protein level of IκBα in HEK293T cells (Figure 2C). Strikingly, co-overexpression of HHEX with IKKβ further increased the levels of p-IKKα/β, p-IκBα, and p-p65 induced by IKKβ overexpression (Figure 2C). Similarly, stable expression of HHEX in both HIEC-6 and HT29 cells dramatically increased the levels of p-IκBα and p-p65 and decreased the protein levels of IκBα shortly after TNF-α stimulation (Figure 2, D and E). The suppressive effect of HHEX overexpression on NF-κB signaling was still observed after long-term TNF-α treatment in HIEC-6 and HT29 cells (Supplemental Figure 1G). Conversely, HHEX knockdown inhibited the activation of IKK kinase activity and the downregulation of IκBα upon TNF-α stimulation (Supplemental Figure 1H). We further established primary intestinal organoids and observed that transient overexpression of HHEX also activated NF-κB signaling and subsequent enhanced transcription of pro-inflammatory cytokines in intestinal organoids (Figure 2, F and G, and Supplemental Figure 1I). Moreover, overexpression of HHEX also promoted TNF-α–induced transcription of pro-inflammatory cytokines and phosphorylation of IκBα and p65 in THP-1 and Jurkat cells, which suggested that HHEX could also play a pro-inflammatory role in immune cells (Supplemental Figure 2, A–D). In this study, we focused on exploring the function and mechanism underlying the pro-inflammatory effects of HHEX in IECs. Notably, the overexpression/knockdown of HHEX also affected the activation of IKK and the transcription of pro-inflammatory cytokine genes under basal conditions without TNF-α treatment in IECs (Figure 2, A and B, and Supplemental Figure 1, E and F). Collectively, these data suggest that HHEX functions as a pro-inflammatory protein, amplifying the TNF-α–induced inflammatory response in IECs.

### Knockout of HHEX alleviates dextran sulfate sodium-induced acute colitis.

**Figure 3:**
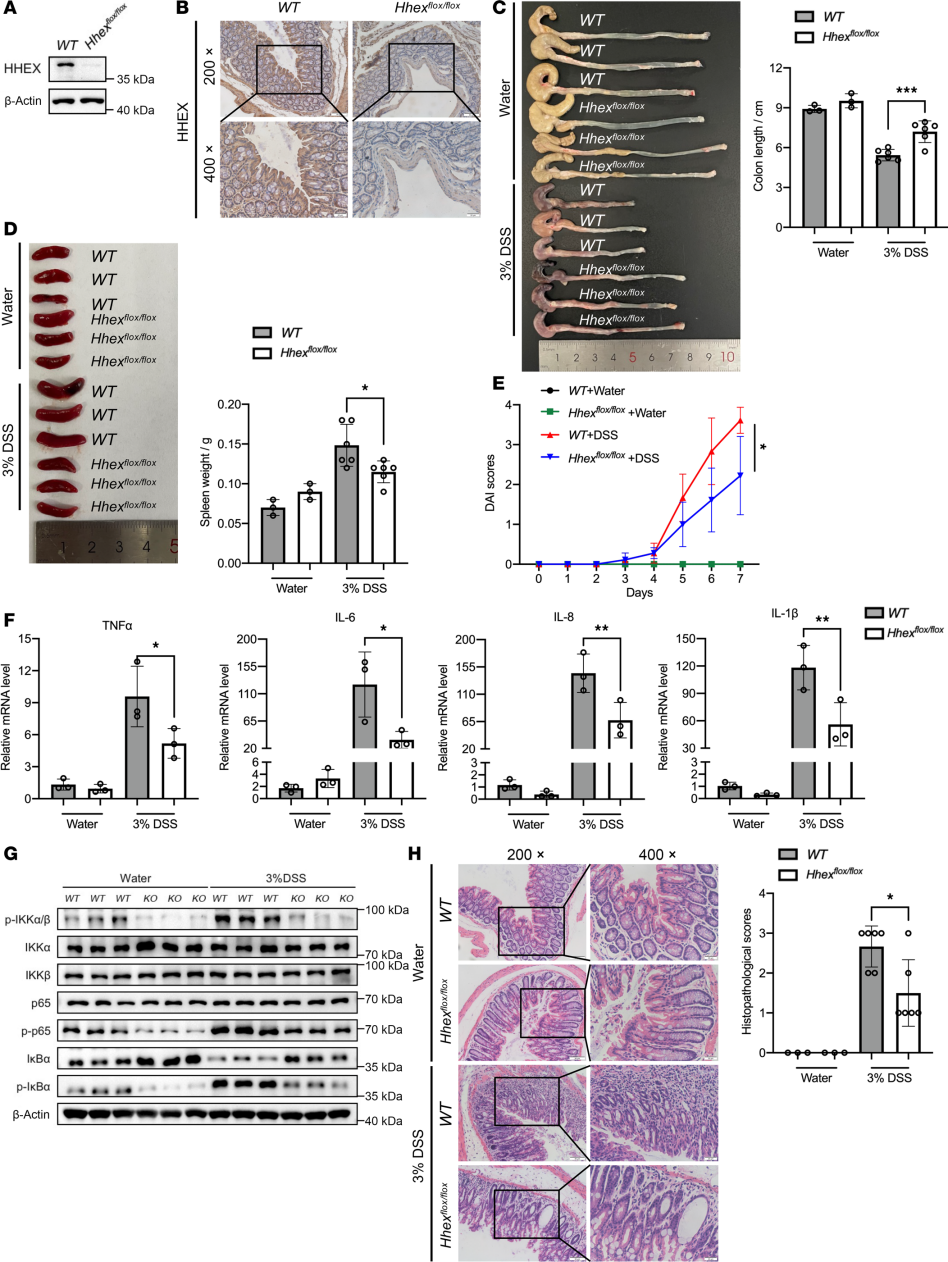
Knockout of HHEX alleviates DSS-induced acute colitis. (**A** and **B**) Western blot analysis and IHC were used to determine the efficacy of HHEX knockdown in the intestinal epithelium of *WT* and *Villin-Hhex^fl/fl^* mice. (**C** and **D**) Representative images displaying the length of the colorectum and the weight of the spleen in the control and DSS-treated *WT* and *Villin-Hhex^fl/fl^* mice. (**E**) Weight loss and severity of diarrhea and bleeding were assessed through DAI scoring in the control and DSS-treated *WT* and *Villin-Hhex^fl/fl^* mice. (**F**) The mRNA levels of pro-inflammatory cytokines in the representative control and DSS-treated *WT* and *Villin-Hhex^fl/fl^* mice were measured via qRT-PCR. (**G**) Western blot analysis of the activation status of the NF-κB pathway in the representative control and DSS-treated *WT* and *Villin-Hhex^fl/fl^* mice. (**H**) Hematoxylin-eosin staining images of representative control and DSS-treated *WT* and *Villin-Hhex^fl/fl^* mice and histopathological scores of all 18 mice (magnification: 200×, left panels; 400×, right panels). *n* = 3 biologically independent samples for the control group; *n* = 6 biologically independent samples for the DSS-treated group. One-way ANOVA with Tukey’s multiple-comparison test (**C**, **D**, and **F**), 2-way ANOVA with Bonferroni’s multiple-comparison test (**E**), and Mann-Whitney *U* test (**H**) were performed to assess statistical significance. * *P* < 0.05, ** *P* < 0.01, *** *P* < 0.001.

To further confirm the pro-inflammatory role of HHEX in vivo, we used *Villin-Hhex^fl/fl^* mice with specific *Hhex* knockout in IECs generated in our previous study (Figure 3, A and B) (23). Compared with *WT* mice, *Villin-Hhex^fl/fl^* mice presented significantly reduced colitis severity after 7 days of DSS treatment, as indicated by increased colon length, decreased spleen weight, and decreased disease activity index (DAI) scores (Figure 3, C–E). Consistent with these observations, the inflammatory cytokine mRNA levels and activation of NF-κB signaling in the intestinal tissue of the *Villin-Hhex^fl/fl^* mice were markedly lower than those in the intestinal tissue of the *WT* controls (Figure 3, F and G). Histopathological analysis further supported these results, revealing less tissue damage and lower pathological scores in the *Villin-Hhex^fl/fl^* mice than in the *WT* mice (Figure 3H). Together, these results demonstrate that HHEX knockout mitigates dextran sulfate sodium (DDS)-induced intestinal mucosal inflammation in mice.

### HHEX interacts with the IKK complex to activate the NF-κB pathway.

**Figure 4:**
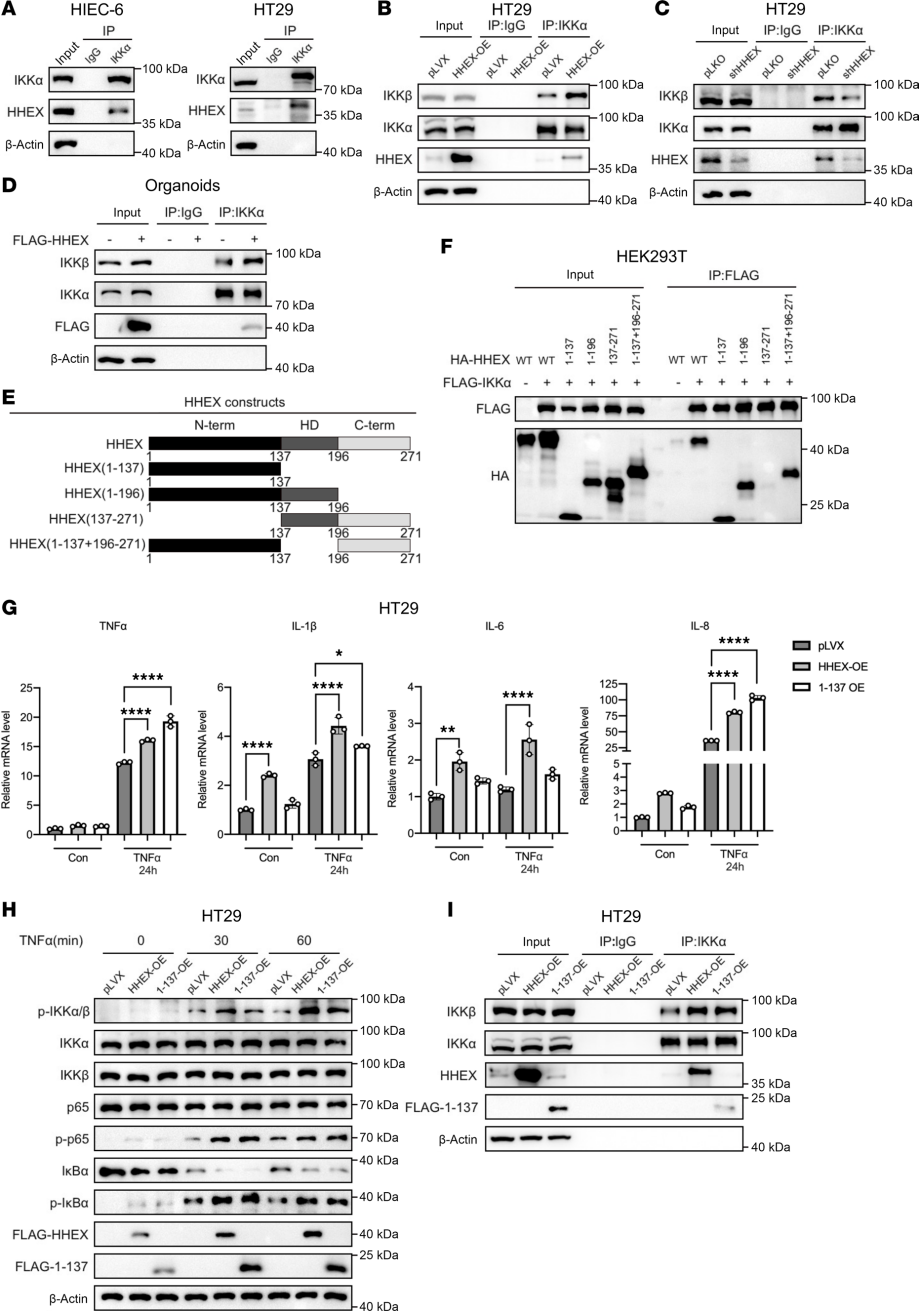
HHEX interacts with IKK complex to activate the NF-κB pathway via its N-terminal domain. (**A**) Co-IP of endogenous HHEX and IKKα in HIEC-6 and HT29 cells. (**B** and **C**) Endogenous co-IP of IKKα and IKKβ in HT29 cells with or without overexpression (**B**) or knockdown (**C**) of HHEX. (**D**) Endogenous co-IP of IKKα and IKKβ in the control and FLAG-HHEX–overexpressing colonic organoids. (**E**) A schematic showing the protein structure of the full-length and truncated HHEX proteins. (**F**) Co-IP of exogenous full-length/truncated HA-HHEX and FLAG-IKKα in HEK293T cells. (**G**) The mRNA levels of pro-inflammatory cytokines in HHEX and N-terminal domain–overexpressing and control HT29 cells were measured via qRT-PCR. The cells were treated with TNF-α (10 ng/mL) for the indicated times before qRT-PCR analysis. (**H**) Western blot analysis of the activation status of the NF-κB pathway in HT29 cells with or without overexpression of HHEX and the N-terminal domain following short-term TNF-α stimulation. (**I**) Endogenous co-IP of IKKα and IKKβ in HT29 cells with or without overexpression of HHEX and the N-terminal domain. “pLKO” indicated the pLKO vector control for gene knockdown of HHEX. The data are presented as the means ± SDs and represent 3 independent experiments in this figure. One-way ANOVA with Tukey’s multiple-comparison test (**G**) was performed to assess statistical significance. * *P* < 0.05, ** *P* < 0.01, **** *P* < 0.0001.

Since HHEX overexpression activates the kinase activity of the IKK complex, we examined whether HHEX could interact with IKKα or IKKβ. To test this hypothesis, we performed exogenous coimmunoprecipitation (co-IP) in HEK293T cells and observed that HHEX interacts with both IKKα and IKKβ (Supplemental Figure 3A). We further performed exogenous co-IP assays after nucleocytoplasmic fractionation of HEK293T and HIEC-6 cells and found that HHEX-IKKα/IKKβ interactions occur in both cytoplasm and nucleus (Supplemental Figure 3, B and C). Endogenous co-IP confirmed the endogenous interaction between HHEX and IKKα (Figure 4A). Interestingly, semiendogenous co-IP in HEK293T cells revealed that the overexpression of HHEX promoted the interaction of IKKα and IKKβ, likely resulting in the activation of the IKK complex (Supplemental Figure 3, D and E). Similar results were observed in HT29 and HIEC-6 cells with stable HHEX overexpression or knockdown, in which the endogenous IKKα/IKKβ interaction was promoted by HHEX overexpression but diminished by HHEX knockdown (Figure 4, B and C, and Supplemental Figure 3, F and G). Furthermore, transient overexpression of HHEX enhanced the interaction between IKKα and IKKβ in primary intestinal organoids, as well as in THP-1 and Jurkat immune cells (Figure 4D and Supplemental Figure 2, E and F).

To further investigate the mechanism by which HHEX promotes the interaction of IKKα/β, we generated a series of plasmids expressing truncated IKKα and IKKβ (24) (Supplemental Figure 4, A and B). Intriguingly, exogenous co-IP experiments in HEK293T cells revealed that both IKKα and IKKβ interact with HHEX via the N-terminal kinase domain but not the LZ domain, which mediates IKKα/β binding and the C-terminal IKKγ/NEMO binding domain (Supplemental Figure 4, C and D). Notably, the overexpression of truncated IKKα (amino acids 1-672), which lacks the IKKγ binding domain, still moderately increased the levels of p-IKKα/β, p-IκBα, and p-p65 in HEK293T cells though to a lesser extent than did full-length IKKα (Supplemental Figure 4E). Interestingly, we observed that the activity of IKKγ binding–deficient truncated IKKα was also increased by the coexpression of HHEX, which suggested that HHEX might positively modulate the IKK complex independent of IKKγ (Supplemental Figure 4E). IKKγ is required for the activation of IKKα/β kinase by TNF-α in the classic NF-κB pathway (11, 25). Thus, we established stable HIEC-6 and HEK293T cells with IKKγ knockdown and assessed the effect of HHEX overexpression on TNF-α–induced activation of IKKα/β (Supplemental Figure 4, F–I). As expected, knockdown of IKKγ largely abrogated the activation of IKKα/β kinase stimulated by TNF-α in both HIEC-6 and HEK293T cells (Supplemental Figure 4, H and I). Furthermore, the increased activation of IKKα/β by HHEX overexpression was attenuated by IKKγ knockdown under both basal conditions and TNF-α–treated conditions (Supplemental Figure 4, H and I). In addition, semiendogenous co-IP revealed that HHEX did not affect the binding of IKKα/β to IKKγ (Supplemental Figure 4, J–M). These findings suggest that HHEX might positively regulate the IKKα/β complex downstream of IKKγ, which is likely independent of IKKγ.

### The N-terminal domain is required for HHEX to interact with and activate the IKK complex.

HHEX consists of 3 domains: an N-terminal proline-rich domain, a homeodomain for DNA binding, and an acidic C-terminal region (12). Next, we characterized the domain of HHEX that is responsible for stabilization (Figure 4E). Exogenous co-IP analysis revealed that the N-terminus of HHEX was required for its interaction with both IKKα and IKKβ (Figure 4F and Supplemental Figure 5A). To further explore whether the N-terminal domain mediates the pro-inflammatory function of HHEX, we generated stable HT29 and HIEC-6 cells overexpressing the HHEX N-terminal domain (Supplemental Figure 5, B and C). Intriguingly, overexpression of N-terminal HHEX still significantly activated the gene transcription of pro-inflammatory cytokine genes in both HT29 and HIEC-6 cells, which was comparable to that of full-length HHEX (Figure 4G and Supplemental Figure 5D). Consistent with these findings, the overexpression of N-terminal HHEX was sufficient to increase the phosphorylation of p65 and promote IκBα degradation in HT29 and HIEC-6 cells (Figure 4H and Supplemental Figure 5, E and F). Unsurprisingly, both endogenous co-IP in HT29 and HIEC-6 cells and semiendogenous co-IP in HEK293T cells confirmed that the N-terminal domain of HHEX increases the interaction between IKKα and IKKβ (Figure 4I and Supplemental Figure 5, G and H). Collectively, these data suggest that HHEX interacts with and activates the IKK complex through its N-terminal domain.

### TNF-α stabilizes the HHEX protein via IKKα kinase in IECs.

**Figure 5:**
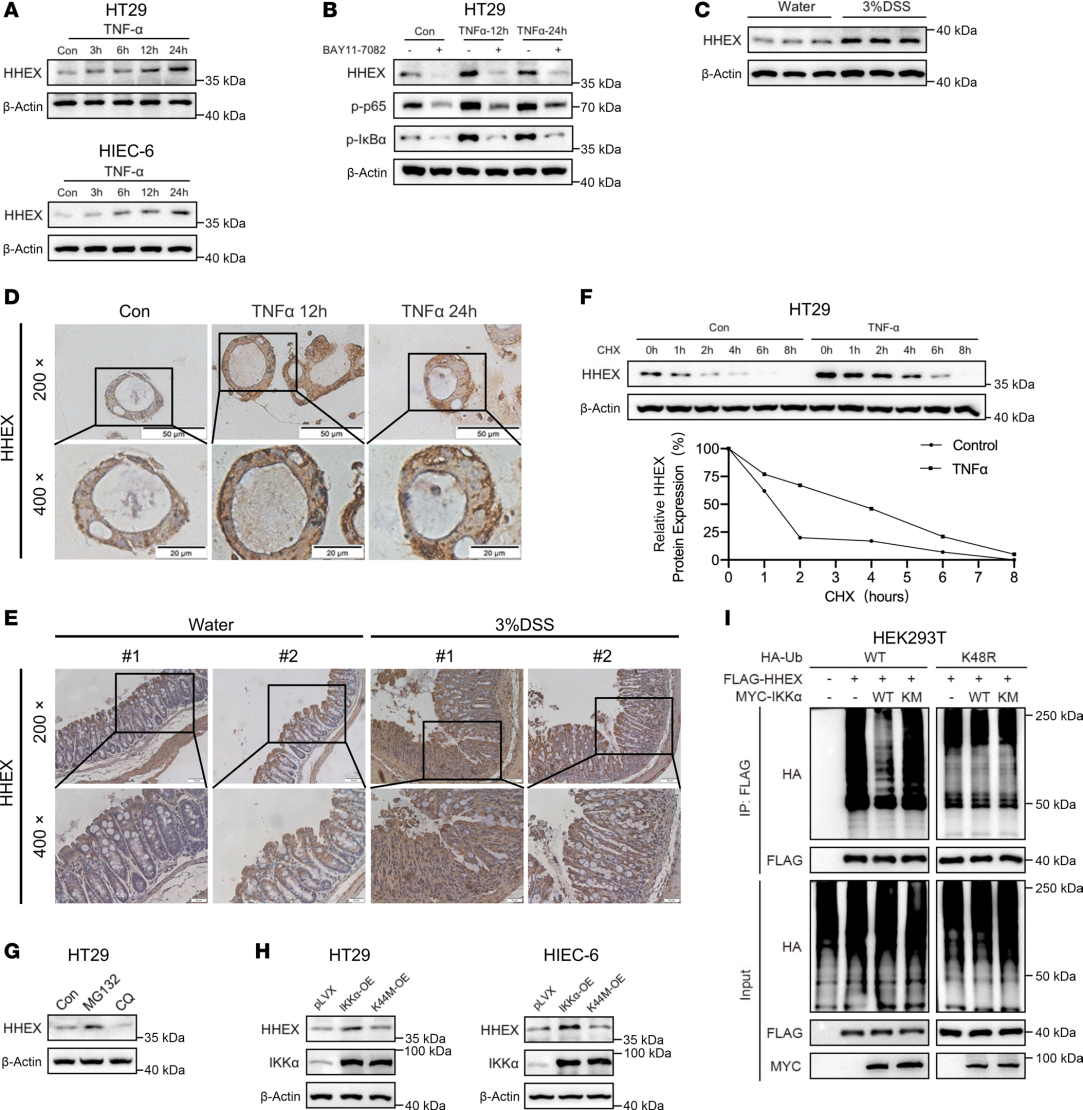
TNF-α stabilizes the HHEX protein via IKKα kinase in IECs. (**A**) Western blot analysis of HHEX expression in HT29 and HIEC-6 cells treated with TNF-α (10 ng/mL) for the indicated times. (**B**) Western blot analysis of HHEX expression and the activation status of the NF-κB pathway in HT29 cells treated with TNF-α (10 ng/mL) alone or in combination with BAY11-7082 (30 μM) for the indicated times. (**C**) Western blot analysis of HHEX expression in colons from 3 pairs of control and DSS-treated mice. (**D**) IHC was used to examine HHEX expression in human colonoids after exposure to TNF-α (10 ng/mL) for the indicated times. (**E**) IHC was used to examine HHEX expression in colon tissues from the control and DSS-induced mice (magnification: 200×, upper panels; 400×, lower panels). (**F**) TNF-α stimulation increased the HHEX half-life in HT29 cells. The cells were pretreated with or without TNF-α (10 ng/mL) for 16 hours and then treated with cycloheximide (CHX) (75 μg/mL) for the indicated times before Western blot analysis. (**G**) Western blot analysis of HHEX expression in the HT29 cells treated with MG132 (10 μM) for 6 hours or chloroquine (CQ) (30 μM) for 24 hours. (**H**) IKKα^WT^ but not IKKα^K44M^ increased HHEX protein expression. Western blot analysis of HHEX expression in HT29 and HIEC-6 cells with or without overexpression of IKKα^WT^ or IKKα^K44M^. (**I**) IKKα^WT^ but not IKKα^K44M^ reduced the level of HHEX K48-linked ubiquitination. Western blot analysis of the level of HHEX ubiquitination in HEK293T cells with or without MYC-IKKα^WT^ or MYC-IKKα^K44M^ overexpression. “pLVX” indicated the pLVX vector control for IKKα overexpression. The data are representative of 3 independent experiments in this figure.

We noted that TNF-α treatment not only increased endogenous HHEX protein levels but also dramatically increased the protein levels of FLAG-HHEX in HT29 and HIEC-6 cells after 12 hours/24 hours of TNF-α treatment, which suggested that TNF-α might stabilize the HHEX protein (Supplemental Figure 1, G and H). Strikingly, FLAG-HHEX protein levels were upregulated 30 minutes after TNF-α treatment in HT29 and HIEC-6 cells (Figure 2, D and E). First, we confirmed the increase in endogenous HHEX protein levels induced by TNF-α treatment in a time-dependent manner, while the HHEX mRNA levels were stable in the HT29 and HIEC-6 cells treated with TNF-α (Figure 5A and Supplemental Figure 6, A and B). Treatment with the NF-κB inhibitor BAY11-7082 abrogated the increase in HHEX protein levels induced by TNF-α treatment (Figure 5B). Similar results were observed in the TNF-α–treated intestinal organoids in vitro and in the DSS-induced colitis mouse model in vivo (Figure 5, C–E). Next, we examined the impact of TNF-α on HHEX protein stability. As expected, TNF-α prolonged the half-life of the HHEX protein in HT29 cells (Figure 5F). Treatment with the proteasome inhibitor MG132 substantially increased HHEX protein levels, whereas treatment with the lysosome inhibitor CQ had a minimal effect, indicating that HHEX undergoes protein degradation via the proteasome (Figure 5G). Consistent with the above observations, the overexpression of IKKα but not the kinase-dead mutant IKKα^K44M^ led to increased protein levels of endogenous HHEX in HEK293T, HT29, and HIEC-6 cells (Figure 5H and Supplemental Figure 6C). In addition, ubiquitination analysis of HHEX revealed that overexpression of IKKα inhibited K48-linked polyubiquitination of HHEX (Figure 5I). Furthermore, upon TNF-α stimulation, HHEX was stabilized to further activate IKK complex via promoting IKKα/IKKβ interaction in IECs (Supplemental Figure 6, D and E). Taken together, our findings indicate that the pro-inflammatory protein HHEX can be stabilized by TNF-α in IECs in a manner dependent on the kinase activity of IKKα.

### HHEX protein degradation is regulated by MID2-mediated K48-linked ubiquitination.

**Figure 6:**
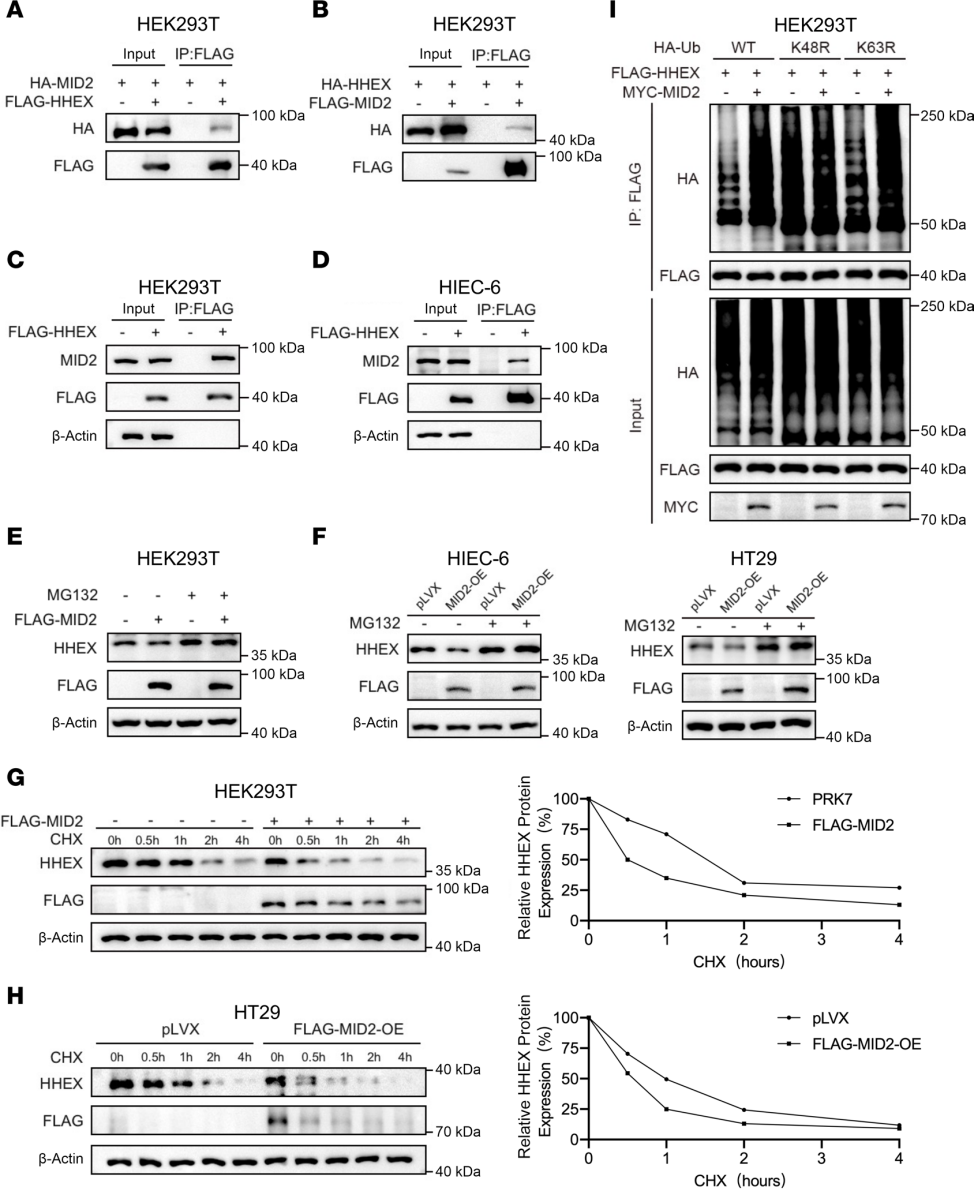
HHEX protein degradation is regulated by MID2-mediated K48-linked ubiquitination. (**A**) Co-IP of exogenous FLAG-HHEX and HA-MID2 in HEK293T cells. (**B**) Co-IP of exogenous HA-HHEX and FLAG-MID2 in HEK293T cells. (**C** and **D**) Semiendogenous co-IP of exogenous FLAG-HHEX and endogenous MID2 in HEK293T (**C**) and HIEC-6 (**D**) cells. (**E** and **F**) MID2 reduced HHEX protein expression. Western blot analysis of HHEX expression in HEK293T (**E**), HIEC-6, and HT29 (**F**) cells with or without MID2 overexpression. The cells were treated with or without MG132 (10 μM) for 6 hours before Western blot analysis. (**G** and **H**) Overexpression of MID2 reduced the HHEX half-life in HEK293T (**G**) and HT29 (**H**) cells. The cells were treated with CHX (75 μg/mL) for the indicated times before Western blot analysis. (**I**) MID2 increased the level of HHEX K48-linked ubiquitination. Western blot analysis of the level of HHEX ubiquitination in HEK293T cells with or without MYC-MID2 overexpression. “pLVX” indicated the pLVX vector control for MID2 overexpression. The data are representative of 3 independent experiments in this figure.

We performed IP-MS/MS analysis of HHEX in HT29 cells but failed to identify the E3 ligase that ubiquitinates HHEX. We also performed exogenous co-IP by screening the E3 ligase expression plasmids constructed previously in-house and detected the interaction between HHEX and the E3 ligase MID2 (Figure 6, A and B). We confirmed the HHEX/MID2 interaction by semiendogenous co-IP in both HEK293T and HIEC-6 cells (Figure 6, C and D). MID2, encoded by the TRIM1 gene, belongs to the tripartite motif (TRIM) protein family and contains a RING domain, 2 B-box zinc fingers, and a coiled-coil domain, which confer E3 ubiquitin ligase activity (26). Transient overexpression of MID2 in HEK293T cells, as well as stable overexpression in HT29 and HIEC-6 cells, resulted in reduced HHEX protein levels (Figure 6, E and F). Moreover, MID2 overexpression no longer affected HHEX protein levels in MG132-treated cells (Figure 6, E and F). The overexpression of MID2 also decreased the half-life of the HHEX protein, which further demonstrated that MID2 promotes HHEX protein degradation (Figure 6, G and H). Consistently, MID2 overexpression upregulated the K48-linked ubiquitination of HHEX (Figure 6I). In conclusion, MID2 promotes HHEX degradation through K48-linked ubiquitination, highlighting a key regulatory mechanism in controlling HHEX protein levels.

### IKKα phosphorylates HHEX at S213 to stabilize the HHEX protein by disrupting the HHEX/MID2 interaction.

**Figure 7:**
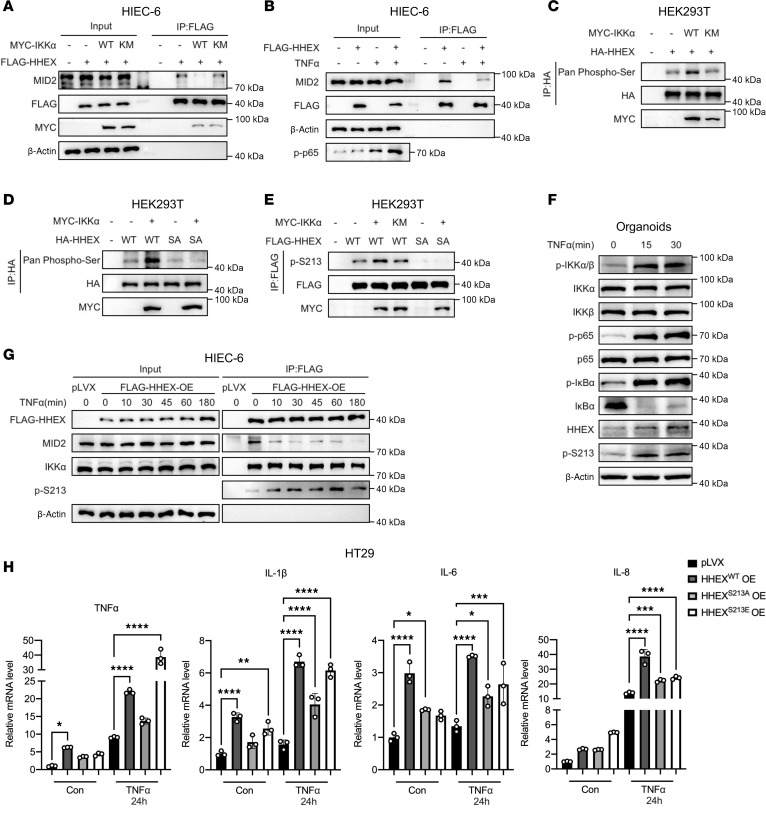
IKKα phosphorylates HHEX at S213 to stabilize the HHEX protein by disrupting the HHEX/MID2 interaction. (**A** and **B**) The interaction between HHEX and MID2 was reduced under inflammatory conditions. Semiendogenous co-IP of exogenous FLAG-HHEX and endogenous MID2 in HIEC-6 cells with or without overexpression of MYC-IKKα^WT^ or MYC-IKKα^K44M^ (**A**). Semiendogenous co-IP of exogenous FLAG-HHEX and endogenous MID2 in HIEC-6 cells with or without TNF-α (10 ng/mL) stimulation for 24 hours (**B**). (**C**) Western blot analysis of the pan-phosphorylation level of HHEX in HEK293T cells with or without overexpression of MYC-IKKα^WT^ or MYC-IKKα^K44M^. (**D**) Western blot analysis of the panphosphorylation level of HHEX^WT^ or HHEX^S213A^ in HEK293T cells with or without overexpression of MYC-IKKα. (**E**) Western blot analysis of the phosphorylation level of the S213 site in HEK293T cells with or without overexpression of MYC-IKKα^WT^ or MYC-IKKα^K44M^. (**F**) Western blot analysis of the phosphorylation level of the S213 site in colonic organoids following short-term TNF-α stimulation. (**G**) Semiendogenous co-IP of exogenous FLAG-HHEX with endogenous MID2 and IKKα and Western blot analysis of S213 phosphorylation levels in control and FLAG-HHEX–overexpressing HIEC-6 cells following short-term TNF-α stimulation. (**H**) The mRNA levels of pro-inflammatory cytokines in HHEX^WT^, HHEX^S213A^-overexpressing, and HHEX^S213E^-overexpressing, and control HT29 cells were measured via qRT-PCR. The cells were treated with TNF-α (10 ng/mL) for the indicated times before qRT-PCR analysis. The data are presented as the means ± SDs and represent 3 independent experiments in this figure. One-way ANOVA with Tukey’s multiple-comparison test (**H**) was performed to assess statistical significance. * *P* < 0.05, ** *P* < 0.01, *** *P* < 0.001, **** *P* < 0.0001.

Next, we assessed whether IKKα affects the interaction between HHEX and its E3 ligase MID2. By performing exogenous co-IP in HEK293T cells, we found that the overexpression of IKKα but not the IKKα^K44M^ mutant diminished the interaction between HHEX and MID2 (Supplemental Figure 7A). Similar results were obtained in HIEC-6 cells through semiendogenous co-IP (Figure 7A). Additionally, TNF-α treatment weakened the HHEX/MID2 interaction in both HEK293T and HIEC-6 cells (Figure 7B and Supplemental Figure 7, B and C). The minimal effect of the kinase-dead mutant IKKα^K44M^ prompted us to explore whether IKKα can phosphorylate HHEX to modulate its degradation. By using a pan–phospho-Ser antibody, we observed that HHEX is a phosphorylated protein and that the overexpression of IKKα increased the phosphorylation level of HHEX (Figure 7C). The PhosphoSite database is a data portal consisting of numerous protein posttranslational modifications identified by large-scale proteomics and substrate sequence specificities for more than 300 protein Ser/Thr kinases (27, 28). Public phosphoproteomic studies have revealed 5 phosphorylation sites in HHEX (S53, S127, S163, S213, and S214). Interestingly, on the basis of the recently profiled substrate specificities for the human Ser/Thr kinome, only S213 was reliably predicted to be phosphorylated by IKKα (28). Furthermore, overexpression of IKKα did not affect the phosphorylation levels of phosphodeficient HHEX^S213A^ (Figure 7D). We also developed a site-specific antibody recognizing p-Ser213 HHEX. The antibody’s specificity for recognizing this phosphorylation was validated by dot blot experiments (Supplemental Figure 7D). Consistent with the results obtained with the pan–phospho-Ser antibody, Western blot analysis revealed that the p-S213 signal was lost in the HHEX^S213A^ mutant, supporting the specificity of the p-Ser213 HHEX antibody (Figure 7E). The p-S213 level of WT HHEX was dramatically increased by coexpressing WT but not kinase-dead mutant IKKα (Figure 7E). Furthermore, we observed that TNF-α treatment induced rapid S213 phosphorylation of HHEX in primary intestinal organoids, correlating with activation of IKK kinase (Figure 7F).

Next, we assessed the impact of phosphorylation of S213 by IKKα on the interaction between HHEX and MID2. Although the S213A mutation had a minimal effect on the HHEX/MID2 interaction, probably due to the relatively low basal IKKα activity in HEK293T cells, the phosphomimetic HHEX^S213E^ exhibited less binding to MID2 than HHEX^WT^ did (Supplemental Figure 7E). In addition, overexpression of IKKα did not diminish the interaction between HHEX^S213A^/HHEX^S213E^ and MID2, which suggested that S213 phosphorylation by IKKα attenuated the HHEX/MID2 interaction (Supplemental Figure 7, F and G). Correspondingly, overexpression of IKKα^WT^ but not IKKα^K44M^ inhibited the MID2-mediated ubiquitination of HHEX (Supplemental Figure 7H). Overexpression of MID2 still increased the ubiquitination level of HHEX^S213A^ and HHEX^S213E^ but slightly increased the ubiquitination level of HHEX^S213E^ compared with HHEX^WT^ (Supplemental Figure 7, I and J). Ubiquitination of HHEX^S213A^ and HHEX^S213E^ by MID2 was not impaired by IKKα overexpression (Supplemental Figure 7, I and J). Additionally, semiendogenous co-IP analysis of the FLAG-HHEX/MID2 interaction showed that TNF-α treatment led to both rapid dissociation of FLAG-HHEX/MID2 and increased p-S213 level of FLAG-HHEX in HIEC-6 and HT29 cells (Figure 7G and Supplemental Figure 7K).

Then, we evaluated the functional effects of S213 phosphorylation on HHEX stability and its pro-inflammatory role upon TNF-α stimulation. HT29 cells stably overexpressing HHEX^WT^, HHEX^S213A^, or HHEX^S213E^ were generated (Supplemental Figure 8, A and B). Half-life experiments revealed that TNF-α treatment did not alter the half-life of HHEX^S213A^ or HHEX^S213E^ proteins (Supplemental Figure 8C). HHEX^S213A^ exhibited a shorter half-life than HHEX^WT^, while the HHEX^S213E^ protein was relatively more stable (Supplemental Figure 8C). Consistent with these results, phosphodeficient HHEX^S213A^ cells presented reduced activation of inflammatory cytokine gene expression after TNF-α treatment (Figure 7H). Interestingly, the phosphomimetic HHEX^S213E^ also reduced the activation of a subset of cytokine genes, such as IL-6 and IL-8, which needs to be explored in future studies (Figure 7H). Nevertheless, these results indicate that IKKα kinase can phosphorylate HHEX at S213 and that S213 phosphorylation leads to attenuated interaction with MID2, MID2-mediated K48-linked ubiquitination, and subsequent protein degradation of HHEX.

### HHEX positively modulates noncanonical NF-κB signaling in IECs.

**Figure 8:**
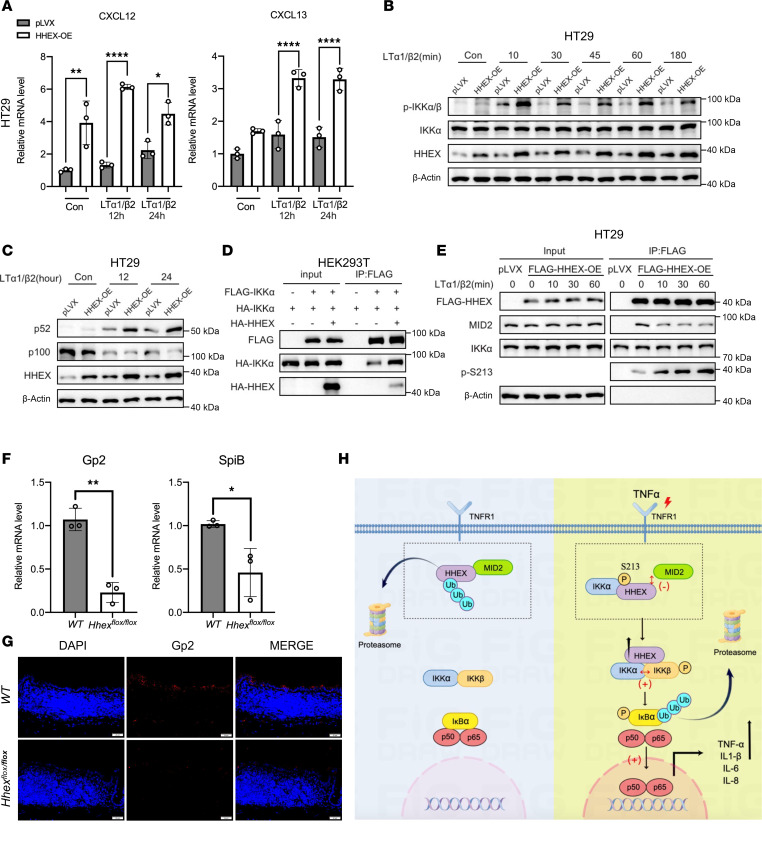
HHEX positively modulates noncanonical NF-κB signaling in IECs. (**A**) The mRNA levels of CXCL12 and CXCL13 in the control and HHEX overexpression HT29 cells were measured via qRT-PCR. The cells were treated with LTα1/β2 (0.2 μg/mL) for the indicated times before qRT-PCR analysis. (**B** and **C**) Western blot analysis of the activation status of the noncanonical NF-κB pathway in control and HHEX overexpression HT29 cells following LTα1/β2 stimulation for the indicated times. (**D**) Exogenous co-IP of FLAG-IKKα and HA-IKKα in HEK293T cells with or without overexpression of HA-HHEX. (**E**) Semiendogenous co-IP of exogenous FLAG-HHEX with endogenous MID2 and IKKα and Western blot analysis of S213 phosphorylation levels in control and FLAG-HHEX–overexpressing HT29 cells following short-term LTα1/β2 stimulation. (**F**) The mRNA levels of the M cell markers *Gp2* and *SpiB* in the *WT* and *Villin-Hhex^fl/fl^* mice were measured via qRT-PCR. (**G**) Representative images of Gp2 immunofluorescence staining in the intestinal epithelium of *WT* and *Villin-Hhex^fl/fl^* mice. Scale bar: 20 μm. (**H**) A schematic diagram of the findings in this study is shown. Model of how HHEX forms a positive feedback regulatory loop under inflammatory conditions. *n* = 3 biologically independent samples for the *WT* and *Villin-Hhex^fl/fl^* mice. The data are presented as the means ± SDs and represent 3 independent experiments in this figure. One-way ANOVA with Tukey’s multiple-comparison test (**A**) and 2-tailed unpaired Student’s *t* test (**F**) were performed to assess statistical significance. * *P* < 0.05, ** *P* < 0.01, **** *P* < 0.0001.

Given the important role of IKKα in noncanonical NF-κB signaling and the HHEX/IKKα positive feedback loop in canonical NF-κB signaling, we investigated whether HHEX could regulate noncanonical NF-κB signaling in IECs. LTβR is highly expressed in HT29 cells, a cell line which is widely used for studying noncanonical NF-κB signaling in IECs (29). HT29 cells were treated with LTα1β2 complex to activate noncanonical NF-κB signaling, and we assessed the gene expression of noncanonical NF-κB–dependent cytokines, such as CXCL12 and CXCL13 (30, 31). Interestingly, stable overexpression of HHEX enhanced the mRNA levels of CXCL12 and CXCL13 at the basal level and after LTα1β2 treatment (Figure 8A). Consistently, HHEX overexpression increased the level of p-IKK, cleavage of p100, and subsequent production of p52 induced by LTα1β2 (Figure 8, B and C). Dimerization and hexamer formation of IKKα are critical for IKKα activation in the noncanonical NF-κB pathway (32). Importantly, we observed that overexpression of HHEX could promote IKKα/IKKα interactions, which could further lead to the activation of IKKα (Figure 8D). Similar to the result of TNF-α treatment, activation of noncanonical NF-κB signaling by LTα1β2 also led to upregulation of HHEX protein level (Figure 8, B and C). In addition, semiendogenous co-IP analysis showed the rapid dissociation of FLAG-HHEX/MID2 upon LTα1β2 treatment in HT29 cells, which was correlated with increased p-S213 level of FLAG-HHEX (Figure 8E). A previous study reported the vital role of epithelial noncanonical NF-κB signaling in M cell (microfold cell) maintenance (33). We reanalyzed the colon samples of *Villin-Hhex^fl/fl^* mice and examined the expressions of M cell markers SPIB and glycoprotein 2 (GP2) in intestines (34, 35). We observed the decreased mRNA levels of *SpiB* and *Gp2* and GP2-positive cell numbers in the colons from *Villin-Hhex^fl/fl^* mice, which suggests the decreased M cells in intestinal *Hhex-KO* mice (Figure 8, F and G). Together, these data indicate that HHEX could be a positive regulator of noncanonical NF-κB signaling in IECs.

## Discussion

The IKK complex is the signal hub for NF-κB activation (11, 25). The IKK complex integrates signals from upstream NF-κB–activating stimuli, including extracellular ligands, such as TNF-α and LPS; intracellular stress, such as DNA damage and reactive oxygen species; and recognition of intracellular pathogens mediated by NOD proteins (11, 25, 36). The activity and formation of the IKK complex are dynamically modulated by posttranslational modifications and protein-protein interactions. In addition to the regulatory subunit NEMO, various proteins, such as ELKS, HSP90, AMBRA1, and CRYAB, have been shown to interact with and modulate IKKα/IKKβ activity under physiological and pathological conditions (37–41). In this study, we discovered that HHEX, a transcription factor, acts as a scaffold protein and agonist of the IKK complex to activate NF-κB in IECs. Thus, HHEX functions as a pro-inflammatory factor during intestinal inflammation. The pro-inflammatory role of HHEX in intestinal inflammation is also consistent with the suppressive role of HHEX during Treg differentiation (42). HHEX-overexpressing Tregs failed to prevent colitis in a transfer-induced colitis model (42). Furthermore, HHEX expression was reportedly increased in Tregs under inflammatory conditions (42). This finding is also consistent with our observation that HHEX expression is upregulated in the intestinal tissues of patients with IBD, supporting the pro-inflammatory function of HHEX in the intestine. In contrast, HHEX was constitutively expressed in microglia and downregulated upon TLR2/4 stimulation, and HHEX overexpression attenuated TLR4-induced expression of inflammation-related genes in microglia (43). These findings suggest that the function of HHEX in inflammation is dependent on the cell context, probably due to the interplay between HHEX and different binding partners in different contexts.

HHEX interacts with many functional proteins, mainly through its N-terminal domain, including the Groucho/TLE (transducin-like enhancer) corepressor family members PML, eIF-4E, and FOXP3 (12, 16, 42, 44). Interestingly, all 3 domains of HHEX contribute to the regulation of FOXP3 target genes and are critical for inhibiting FOXP3 and Treg immunosuppressive functions (42). We found that the N-terminal domain of HHEX also mediates the interaction between HHEX and IKKα/IKKβ. Intriguingly, we observed that the N-terminal domain of HHEX is sufficient to stabilize the IKKα/IKKβ complex and drive the inflammatory response. The chaperone HSP90 can act as a stabilizing factor of IKK through interactions between its cochaperone Cdc37 and the kinase domains of IKKα/IKKβ (38, 39). Since both kinase domains of IKKα and IKKβ interact with HHEX, we speculate that HHEX might serve as a stabilizer of kinase domain folding, similar to HSP90. Further studies regarding the biochemical and structural nature of the HHEX-IKKα/IKKβ complex are anticipated to shed light on the mechanism of HHEX-dependent IKK activation and provide structural insights for the development of new IKK inhibitors that target HHEX-IKK–interacting interfaces. Notably, TLE1, the corepressor of HHEX, is a CD susceptibility gene (45). TLE1 can interact with the cytoplasmic sensor NOD2 and inhibit NOD2/NF-κB signaling (46–48). HHEX can promote the nuclear retention of TLE proteins, including TLE1 (49). In this context, HHEX could also promote the inflammatory response of NOD2/NF-κB signaling through the nuclear retention of TLE1 and the inhibition of the TLE1/NOD2 interaction in the cytoplasm (Supplemental Figure 9, A–D).

HHEX is expressed in both the cytoplasm and nucleus (44). In addition to its classic transcriptional repressor function in the nucleus, HHEX can associate with eIF-4E in both the nucleus and cytoplasm to retard CCND1 mRNA transport from the nucleus to the cytoplasm (44). Similarly, our study revealed that HHEX can interact with IKKα in both the nucleus and the cytoplasm. Given that NF-κB signaling transduction occurs in the cytoplasm, our study revealed the cytoplasmic and transcription-independent functions of HHEX during the inflammatory response. Moreover, IKKα can play a crucial role in the nucleus by regulating NF-κB–dependent and NF-κB–independent gene transcription. Thus, HHEX might recruit IKKα to specific gene loci to modulate gene transcription, which could be explored in future studies. Moreover, IKKα plays a critical role in noncanonical NF-κB signaling, which not only mediates the survival of naive B cells but also regulates germinal center (GC) formation and maintenance in B cells (50). Recent studies have also revealed the vital role of HHEX in B cell development in the spleen, bone marrow, and GC (51, 52). In this study, we demonstrated HHEX could also act as an agonist of the IKKα complex in noncanonical NF-κB signaling in IECs. Thus, we hypothesize that HHEX might function through noncanonical NF-κB signaling in immune cells, a possibility that needs to be explored in future studies.

As a multifunctional regulator of cell fate, HHEX is dynamically regulated at both transcriptional and posttranslational levels in response to various stimuli (12, 16). TGF-β/SMAD3 and BCL6 suppress HHEX transcription in CD4^+^ T cells and GC B cells, respectively (42, 51). HHEX is known as a proteosome-interacting protein and is degraded by the proteasome (53). We determined that MID2/TRIM1 is an E3 ligase that ubiquitinates and destabilizes HHEX in IECs. Emerging reports suggest that TRIM proteins are involved in multiple steps of the TNF-α–induced NF-κB pathway (54). HHEX protein destabilization by TRIM1 is consistent with the findings of a recent study reporting that TRIM1 is a negative regulator of the canonical NF-κB pathway and inflammation in CRC cells (55). In particular, TNF-α stimulation triggers HHEX phosphorylation by IKKα at S213, which disrupts the HHEX/MID2 interaction and consequently increases HHEX protein stability. The upregulation of HHEX upon TNF-α stimulation results in the formation of a new positive feedback loop with the NF-κB pathway through the activation of the IKK complex. Since HHEX-promoted IKK activation upon TNF-α stimulation can be largely abrogated by the loss of NEMO, we consider HHEX an agonist but not an alternative signal transducer of the NF-κB pathway, similar to NEMO. In addition, HHEX overexpression dramatically increased IKK activation under basal conditions without TNF-α treatment in IECs, indicating that HHEX might activate IKK and promote inflammation independently of TNF-α stimulation. Thus, constitutive expression of HHEX could be responsible for the limited therapeutic effect of TNF-α antagonists, such as infliximab, which warrants exploration in patients with IBD with infliximab treatment failure.

There are 4 genes located around the IBD susceptibility SNP rs7911264, including HHEX (20, 21). Our study suggests that HHEX could be the key gene related to the SNP rs7911264 that confers genetic susceptibility to predispose individuals to IBD. It would be interesting to explore the mechanism and clinical relevance of rs7911264 in modulating HHEX expression in patients with IBD in future studies. Overall, we identified HHEX as a positive regulator of the IKK complex that activates the NF-κB pathway. Inflammatory stimuli can stabilize HHEX by IKKα-dependent phosphorylation. This study highlights the positive feedback loop of HHEX-IKKα during the intestinal inflammatory response, which could be disrupted for IBD treatment by targeting the interfaces of the HHEX-IKK interaction (Figure 8H).

## Methods

### Sex as a biological variable.

Our study collected clinical samples from male and female patients. Sex was not considered as a biological variable. For animal experiments, our study examined only male mice, since male animals exhibited stronger susceptibility, lower hormone variability, and smaller experimental variability (56–60). It is unknown whether the findings are relevant for female mice.

### Human tissue samples and IHC.

Paired inflamed and noninflamed colon tissues were obtained from patients with IBD who underwent colectomy at the Department of Colorectal Surgery, Xinhua Hospital, Shanghai Jiaotong University School of Medicine. In accordance with the standard protocol, IHC was performed using the heat-induced epitope retrieval method. The semiquantitative analysis was as follows: the staining intensity was divided into 0 (no staining), 1 (weak staining), 2 (moderate staining), or 3 (strong staining), and the staining percentage was divided into 1 (<25%), 2 (25%–50%), 3 (50%–75%), or 4 (>75%). IHC staining was evaluated by 2 independent pathologists, and the immunoreactive score (IRS) was obtained by multiplying the staining intensity by the percentage of stained cells (61). We scored HHEX protein levels in the cytoplasm and nucleus, separately. Low and high expression levels of HHEX in the cytoplasm were defined as an IRS of < 6 and ≥ 6, respectively, whereas those in the nucleus were defined as an IRS of < 3 and ≥ 3, respectively.

### Organoid culture.

For human intestinal organoids, fresh normal colonic mucosa was collected from colorectal surgical resections at Xinhua Hospital. For mouse intestinal organoids, colonic tissues were harvested after euthanizing the mice. Then, the colonic crypts were isolated according to the method described by Sato et al. (62). Human Colonic Organoid Kit (Serum-free) (K2003-HC) and Mouse Colonic Organoid Kit (Serum-free) (K2204-MC) were purchased from bioGenous. Matrix for Organoid culture (40191ES08) and primocin (60281ES50) were purchased from YEASON. SB202190 and Y27632 were purchased from Selleck Chemicals. Newly seeded organoids were supplemented with SB202190, Y27632, and primocin. The Colonic Organoid Basal Medium was changed every 2 days.

### Cell lines and reagents.

All cell lines were purchased from the American Type Culture Collection and validated through short tandem repeat analysis. HT29 and HEK293T cells were cultured in DMEM supplemented with 10% fetal bovine serum, streptomycin, and penicillin (Gibco) at 37°C in 5% CO_2_. HIEC-6 cells were cultured in RPMI 1640 medium supplemented with 10% fetal bovine serum, streptomycin, and penicillin (Gibco).

The transient expression, truncated form, and retroviral plasmids of HHEX, IKKα, and IKKβ have been described in our previous study (40). The shHHEX-1 and shHHEX-2 plasmids were also described in our previous study (23). pRK7-FLAG-MID2, pcDNA-HA-MID2, pcDNA-MYC-MID2, pEGFP-C3-GFP-MID2, and pRK7-FLAG-NOD2 were constructed by using the ClonExpress I One Step Cloning Kit (Vazyme). The HA-Ub, HA-Ub-K48R, and HA-Ub-K63R plasmids were provided by Wei Yu from Fudan University, Shanghai, China. The expression plasmid of the HHEX mutant was generated via a KOD mutagenesis kit (Toyohiro) according to the manufacturer’s instructions. In addition, FLAG-HHEX^WT^, FLAG-HHEX^S213A^, and FLAG-HHEX^S213E^ and FLAG-HHEX-1-137 and FLAG-MID2 were amplified by PCR using 5′ primers containing the FLAG-tag sequence and then cloned and inserted into the pLVX-puro lentiviral vector. For genetic knockdown of IKKγ, shRNAs targeting this gene were constructed using the pLKO.1 system. The targeting sequence was as follows: IKKγ-sh: 5′-GCTCGATCTGAAGAGGCAGAA-3′.

Human and Mouse TNF-α and Recombinant Human Lymphotoxin alpha1/beta2 Protein (LTα1/β2) were purchased from R&D Systems, and BAY11-7082 was purchased from Selleck Chemicals. The proteasome inhibitor MG132 and protein synthesis inhibitor CHX were purchased from Sigma-Aldrich, and the lysosome inhibitor CQ was purchased from MedChemExpress. Lipofectamine 3000 was purchased from Invitrogen.

Antibodies specific for the following proteins were used in this study: HHEX (MAB83771, R&D Systems, dilutions: 1:1,000 for WB and 1:100 for IHC), IKKα (A2062, ABclonal, dilutions: 1:1,000 for WB and 1:500 for IP), IKKβ (A19606, ABclonal, dilutions: 1:1,000 for WB), IKKγ (A0917, ABclonal, dilutions: 1:1,000 for WB), MID2 (PA5-117727, Invitrogen, dilutions: 1:1,000 for WB), p-IKKα/β (2078S, Cell Signaling Technology, dilutions: 1:1,000 for WB), p-IκBα (AF1870, Beyotime, dilutions: 1:1,000 for WB), IκBα (AF1282, Beyotime, dilutions: 1:1,000 for WB), p-p65 (AN371, Beyotime, dilutions: 1:1,000 for WB), p65 (AF1234, Beyotime, dilutions: 1:1,000 for WB), NFκB2 (15503-1-AP, Proteintech, dilutions: 1:1,000 for WB), TLE1 (66216-1-lg, Proteintech, dilutions: 1:1,000 for WB), NOD2 (A25228, ABclonal, dilutions: 1:1,000 for WB), GP2 (GTX134145, GeneTex, dilutions: 1:100 for immunofluorescence [IF]), HA (3724, Cell Signaling Technology, dilutions: 1:3,000 for WB), FLAG (14793, Cell Signaling Technology, dilutions: 1:1,000 for WB), MYC (2276, Cell Signaling Technology, dilutions: 1:1,000 for WB), GFP (sc9996, Santa Cruz Biotechnology, dilutions: 1:1,000 for WB), pan-Ser (sc-81514, Santa Cruz Biotechnology, dilutions: 1:1,000 for WB), p-S213 (Sanyou Bio, dilutions: 1:1,000 for WB), β-actin (A2228, Sigma-Aldrich, dilutions: 1:10,000 for WB), Lamin B1 (12987-1-AP, Proteintech, dilutions: 1:1,000 for WB), β-tubulin (A12289, ABclonal, dilutions: 1:1,000 for WB), and normal rabbit IgG (2729, Cell Signaling Technology, dilutions: 1:500 for IP).

### Preparation method for p-S213 antibody by Sanyou Bio.

For stage 1, peptide design and synthesis, 2 peptides targeting the protein of interest were designed and synthesized, each conjugated to keyhole limpet hemocyanin (KLH) and subjected to phosphorylation modification. The specific amino acid sequences were KKEELESLDSSC and EELESLDSSCD, respectively. For stage 2, animal immunization, phosphorylated Peptide 1-KLH and Peptide 2-KLH were used for single immunization (2 mice/1 rabbit per group), and the 2 phosphorylated peptide-KLH conjugates were applied for cross-immunization (2 mice/1 rabbit). Routine immunization was conducted 4 times at a 2-week interval, followed by a booster immunization at the end of the routine immunization schedule. For titer detection, serum samples were collected from the immunized animals after the third and fourth immunizations for antibody titer determination. For stage 3, validation of polyclonal antibody positivity, the positivity of the polyclonal antibody was validated by Dot blot assay (Supplemental Figure 7D).

### Western blot analysis, immunoprecipitation, and ubiquitination assay.

For direct Western blot analysis, the cells were lysed in NP-40 lysis buffer supplemented with protease inhibitor cocktail (Roche) (1% NP-40, 50 mM Tris HCl at pH 7.5, 150 mM NaCl, 1 mM PMSF, 25 mM NaF, and 1 mM Na_3_VO_4_). For routine exogenous/semi-endogenous immunoprecipitation, the cells were lysed using 0.3% NP-40 lysis buffer, and then, immunoprecipitation was performed using anti-FLAG/HA/GFP magnetic beads (Bimake) at 4°C for 3 hours. For immunoprecipitation for ubiquitination detection, the cells were pretreated with MG132 for 4 hours before lysis. For endogenous immunoprecipitation, the cell lysates were incubated overnight at 4°C with the designated primary antibodies or control IgG bound to A/G protein agarose. After boiling at 95°C for 10 minutes, the precipitated protein from the beads was washed with 1× loading buffer. The relative quantitative analysis of immunoblots was performed by measuring the densitometry values of Western blot bands. The relative protein levels were calculated by using the ratio formula of integrated density value.

### qRT-PCR.

Total RNA was extracted from tissues or cells using TRIzol reagent (TaKaRa). Reverse transcription was performed via the PrimeScript RT Master Mix Kit (TaKaRa). SYBR Premix ExTaq (Yeasen) and an Applied Biosystems 7500 rapid real-time PCR system were used for the qPCR analysis. The relative expression level of mRNA was determined via the 2^−ΔΔCt^ method and normalized to the expression level of β-actin. All the experiments were conducted in triplicate. The detailed sequences of the qRT-PCR primers used in this study are shown in Supplemental Table 1.

### Transient transfection of cell lines and organoids.

For HEK293T cells, transient transfection was performed by using polyethylenimine (PEI). For other cell lines, cells were seeded 1 day before transfection to reach 70%–90% confluence at the time of transfection. Transfection was performed by using Lipofectamine 3000 reagent (Thermo Fisher Scientific): the overexpression plasmid (supplemented with P3000 reagent, Thermo Fisher Scientific) and Lipofectamine 3000 reagent were separately diluted in Opti-MEM (Gibco) at a DNA/Lipofectamine 3000 ratio of 1:1. The mixture was incubated for 10–15 minutes, and then the DNA-lipid complexes were added to the cell culture. Cells were maintained at 37°C for 24–48 hours before subsequent analysis.

For organoids, those in the logarithmic growth phase were evenly dispersed in Matrigel in 24-well plates, with the number controlled at approximately 100 per well to avoid aggregation. Fresh medium was replaced 1–2 days before transfection. Following a similar protocol for cell lines, Lipofectamine 3000 reagent was used at a DNA/Lipofectamine 3000 ratio of 1:2.

### Establishment of cell lines with stable knockdown and overexpression.

For knockdown of the HHEX and IKKγ genes in cells and overexpression of HHEX, MID2, and IKKα, lentiviruses were generated in HEK293T cells by transfecting designated lentiviral plasmids and pMD.2G/psPAX2 packaging/envelope plasmids. Then, the cells were infected with viral particles for 1 day and selected with 1 μg/mL puromycin for 1 week to establish stable gene knockdown or overexpression cell lines for subsequent detection. Two shRNAs targeting HHEX were constructed by using the pLKO.1 system, referred to as shHHEX_1 and shHHEX_2, respectively. “pLKO” indicates the pLKO vector control for gene knockdown of HHEX. pLVX-puro lentiviral system was used for establishing stable cells overexpressing HHEX, where “pLVX” indicates the pLVX vector control for HHEX overexpression.

### Mice and mouse model of DSS-induced colitis.

*Hhex^fl/fl^* mice and *Villin-Cre* mice on a C57BL/6 background were purchased from Jackson Laboratories (Stock Nos. 025396 and 004586, respectively) and crossed to generate *Villin-Hhex^fl/fl^* mice for the DSS-induced model, which was described in our previous study (23). Three percent DSS was dissolved in the drinking water to induce colitis in the mice. Eight-week-old male mice (20–24 g) were randomly divided into a control group or a DSS-induced group, and changes in body weight, activity, and fecal characteristics were monitored. All the mice were euthanized after 7 days of DSS-induced colitis. The colon and spleen were dissected, and their lengths and weights were compared. Protein and RNA were extracted from a portion of the colon tissue. In addition, the colon tissues of these mice were fixed with 4% paraformaldehyde for IHC and hematoxylin-eosin staining for further experiments. In this study, the *WT* mice used as controls were unfloxed, age-matched, male littermates of the *Villin-Hhex^fl/fl^* mice.

### DAI and histopathological scoring.

After DSS induction, the mice were monitored daily for body weight, fecal characteristics, and the presence of gross blood or occult blood in stool. A standard scoring was performed using the DAI assessment method, with the scoring criteria detailed as follows: percentage of body weight loss: 0 points for no weight change, 1 point for 1%–5% loss, 2 points for 5%–10% loss, 3 points for 10%–20% loss, and 4 points for >20% loss; stool consistency: 0 points for normal stool, 2 points for loose stools, and 4 points for diarrhea; stool bleeding: 0 points for normal (no bleeding), 2 points for positive occult blood, and 4 points for gross blood in stool.

The total score from 3 parameters was divided by 3 to obtain the final DAI score for subsequent statistical analysis (63).

Histopathological scores were evaluated on the basis of neutrophil infiltration, crypts, cross-sectional involvement, and erosion or ulceration in mouse intestinal slides stained with hematoxylin-eosin (64).

### IF.

For IF staining of mouse intestinal sections, SABC Cy3-conjugated anti-rabbit IgG antibody (SA1074, BOSTER) served as the secondary antibody. Following this, nuclei were counterstained with DAPI, and all stained samples were mounted with coverslips before being observed and analyzed under a laser scanning confocal microscope (Olympus).

### Statistics.

Statistical analysis was conducted using SPSS version 19.0 (IBM 2010) and GraphPad Prism 8.0. The quantitative data are presented as the means ± SDs. For comparisons between 2 groups, a 2-tailed, paired/unpaired Student’s *t* test was used. One-way ANOVA was used to evaluate the statistical significance of experiments with >2 independent groups. Tukey’s test was used as a post hoc test for pairwise comparisons via 1-way ANOVA. For DAI evaluation, 2-way ANOVA was conducted to assess statistical significance. For the paired IHC score data, the Wilcoxon signed-rank test was used. The Mann-Whitney *U* test was used to compare the differences in pathological scores between the HHEX high- and low-expression groups. Spearman’s correlation analysis was used to determine the relationship between the pathological score and HHEX expression in tissues. All the statistical tests were 2 sided, and a *P* < 0.05 was considered to indicate a significant difference.

### Study approval.

This study was approved by the Ethics Committee of Xinhua Hospital (No. XHEC-NSFC-2022-113). The Animal Ethical and Welfare Committee of XHEC (Approval No. XHEC-F-2025-015) approved the mouse studies. Written informed consent was obtained for all collected samples.

### Data availability.

The raw data supporting the conclusions of this article, including those in the Supporting Data Values file, will be made available by the authors.

## Author contributions

PD, CYL, and ZW designed the research. ZH, WX, and ZF performed the experiments and/or analyzed the data. ZH and CYL wrote the manuscript. WD, YW, and YY assisted in some analyses and collected the specimens from patients. FL, ZD, WT, WO, WG, and YWC assisted in some analyses and reviewed the manuscript. All the authors approved the final version.

## Conflict of interest

The authors have declared that no conflict of interest exists.

## Funding support

- National Natural Science Foundation of China (No. 82570638, 82470549, 82270549).
- Natural Science Foundation of Shanghai (No. 25ZR1402360, 22ZR1440500).
- The Clinical Research Special Project of Shanghai Municipal Health Commission (20254Y0118).
- The Interdisciplinary Program of Shanghai Jiaotong University (YG2025QNB40).

## Supplementary Material

Supplemental data

Unedited blot and gel images

Supporting data values

## Version 1 03/17/2026

In-Press Preview

## Version 2 05/15/2026

Electronic publication
