# Supplementary material for: An HHEX/IKKα positive feedback loop promotes intestinal inflammation
Source: J Clin Invest. 2026 Mar 17;136(10):e192074. doi: 10.1172/JCI192074 (PMC13178647; doi:10.1172/JCI192074)
Supplement: Supplemental data [file jci-136-192074-s169.pdf]

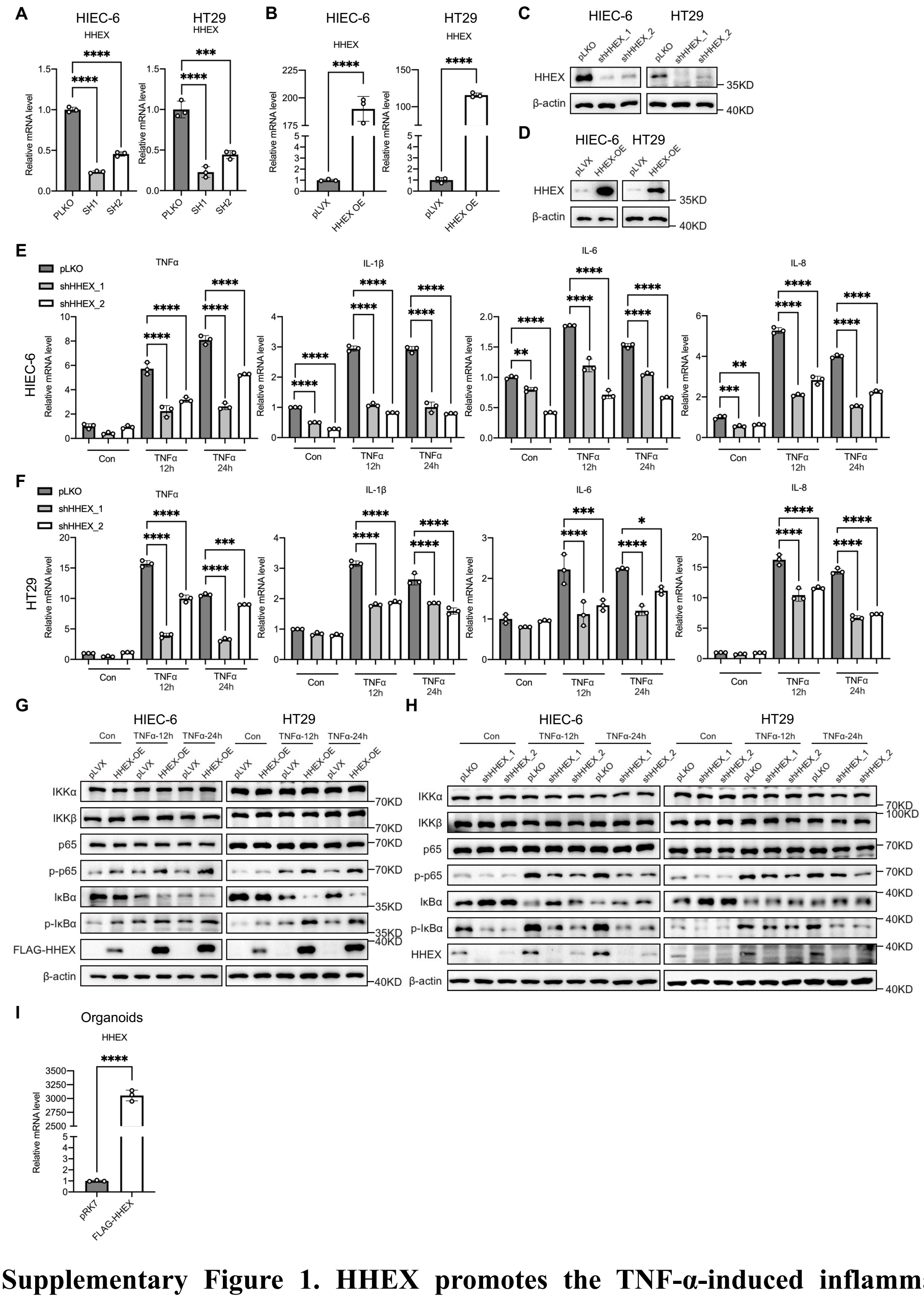

Supplementary Figure 1. HHEX promotes the TNF-α-induced inflammatory response in human intestinal epithelial cells.

(A-D) The efficacy of HHEX knockdown (A, C) and overexpression (B, D) were examined in HIEC-6 and HT29 cells via qRT–PCR(A-B) and Western blot analysis (C-D). (E-F) The mRNA levels of proinflammatory cytokines in the HHEX-knockdown and control HIEC-6 (E) and HT29 (F) cells were measured via qRT–PCR. The cells were treated with TNF-α (10 ng/ml) for the indicated times before qRT–PCR analysis.

(G) Western blot analysis of the activation status of the NF-κB pathway in the control and FLAG-HHEX-overexpressing HIEC-6 or HT29 cells after TNF-α stimulation for the indicated times. (H) Western blot analysis of the activation status of the NF-κB pathway in the control and HHEX-knockdown HIEC-6 or HT29 cells after TNF-α stimulation for the indicated times. (I)The efficacy of FLAG-HHEX overexpression in colonic organoids was examined via qRT–PCR. "pLKO" indicated the pLKO vector control for gene knockdown of HHEX, "pLVX" indicated the pLVX vector control for HHEX overexpression. The data are presented as the means ± SDs and represent 3 independent experiments in this figure. One-way ANOVA with Tukey's multiple comparison test (A), and Two-tailed, unpaired Student's t test (B, I) were performed to assess statistical significance. * $P < 0.05$, ** $P < 0.01$, *** $P < 0.001$, **** $P < 0.0001$.

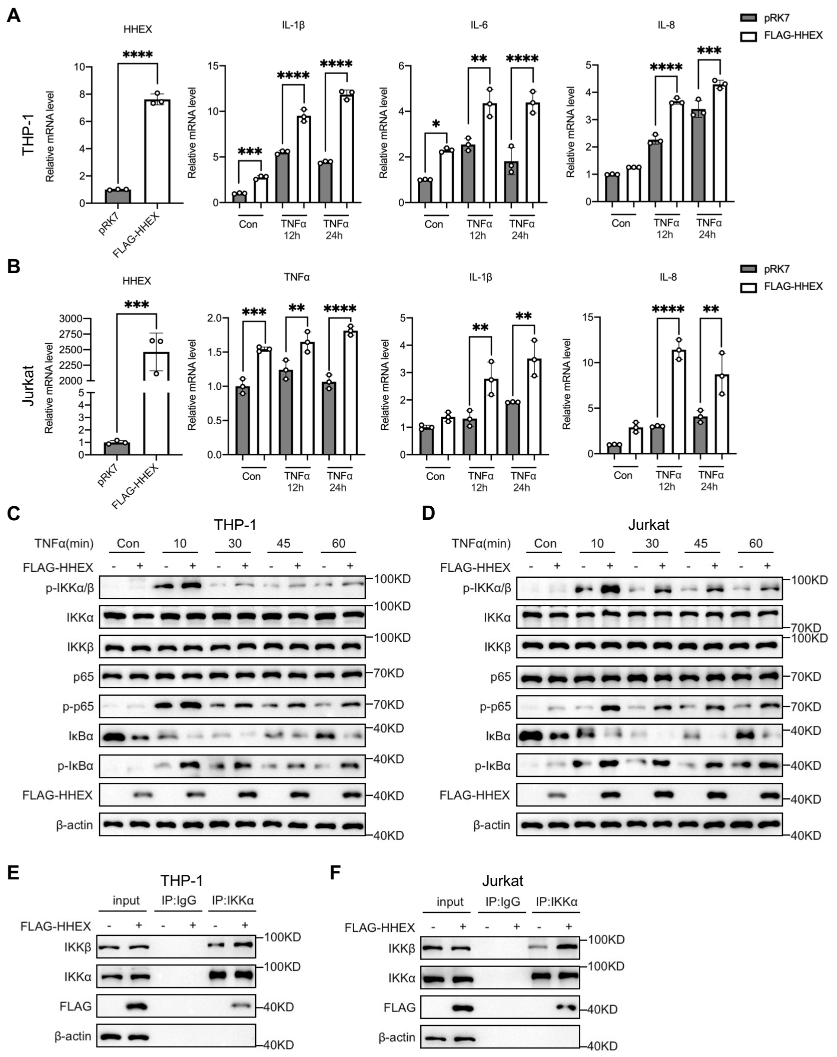

Supplementary Figure 2. HHEX promotes inflammatory response in THP-1 and Jurkat cells.

(A-B) qRT–PCR was used to measure the mRNA levels of FLAG-HHEX (to verify overexpression efficacy) and proinflammatory cytokine genes in FLAG-HHEX-overexpressing and control THP-1 (A) or Jurkat (B) cells. The cells were treated with TNF-α (10 ng/ml) for the indicated times before qRT–PCR analysis. (C-D) Western blot analysis of the activation status of the NF-κB pathway in the control and FLAG-HHEX-overexpressing THP-1 (C) or Jurkat (D) cells following short-term TNF-α stimulation. (E-F) Endogenous co-IP of IKKα and IKKβ in the control and FLAG-HHEX-overexpressing THP-1 (E) or Jurkat (F) cells. The data are presented as the means ± SDs and represent 3 independent experiments in this figure. One-way ANOVA with Tukey's multiple comparison test and two-tailed, unpaired Student's t test (A-B)

were performed to assess statistical significance. * $P < 0.05$, ** $P < 0.01$, *** $P < 0.001$, **** $P < 0.0001$.

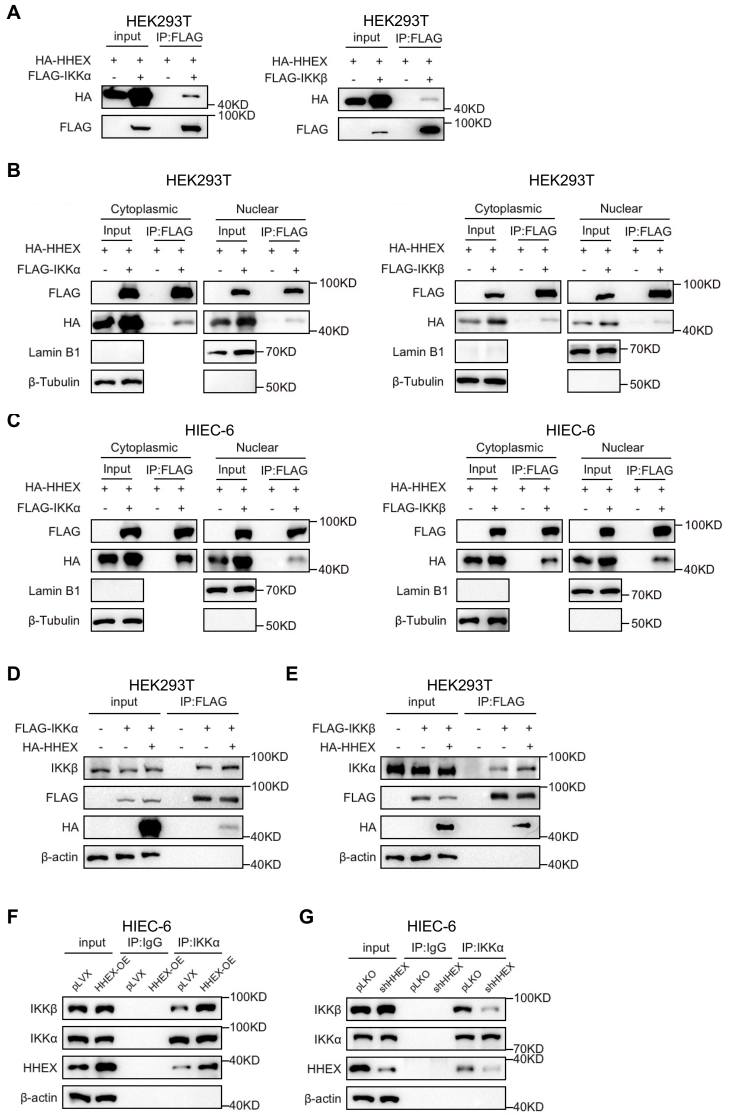

Supplementary Figure 3. HHEX interacts with IKK complex in both the cytoplasm and the nucleus.

(A) Co-IP of exogenous HA-HHEX and FLAG-IKKα/FLAG-IKKβ in HEK293T cells. (B-C) Co-IP of exogenous HA-HHEX and FLAG-IKKα/FLAG-IKKβ in HEK293T (B) or HIEC-6 (C) cells following cytoplasmic and nuclear fractionation. (D) Semiendogenous co-IP of exogenous FLAG-IKKα and endogenous IKKβ in HEK293T cells with or without HA-HHEX overexpression. (E)Semiendogenous co-IP of exogenous FLAG-IKKβ and endogenous IKKα in HEK293T cells with or without HA-HHEX overexpression. (F-G) Endogenous co-IP of IKKα and IKKβ in HIEC-6 cells with or without overexpression (F) or knockdown (G) of HHEX. The data are representative of 3 independent experiments in this figure.

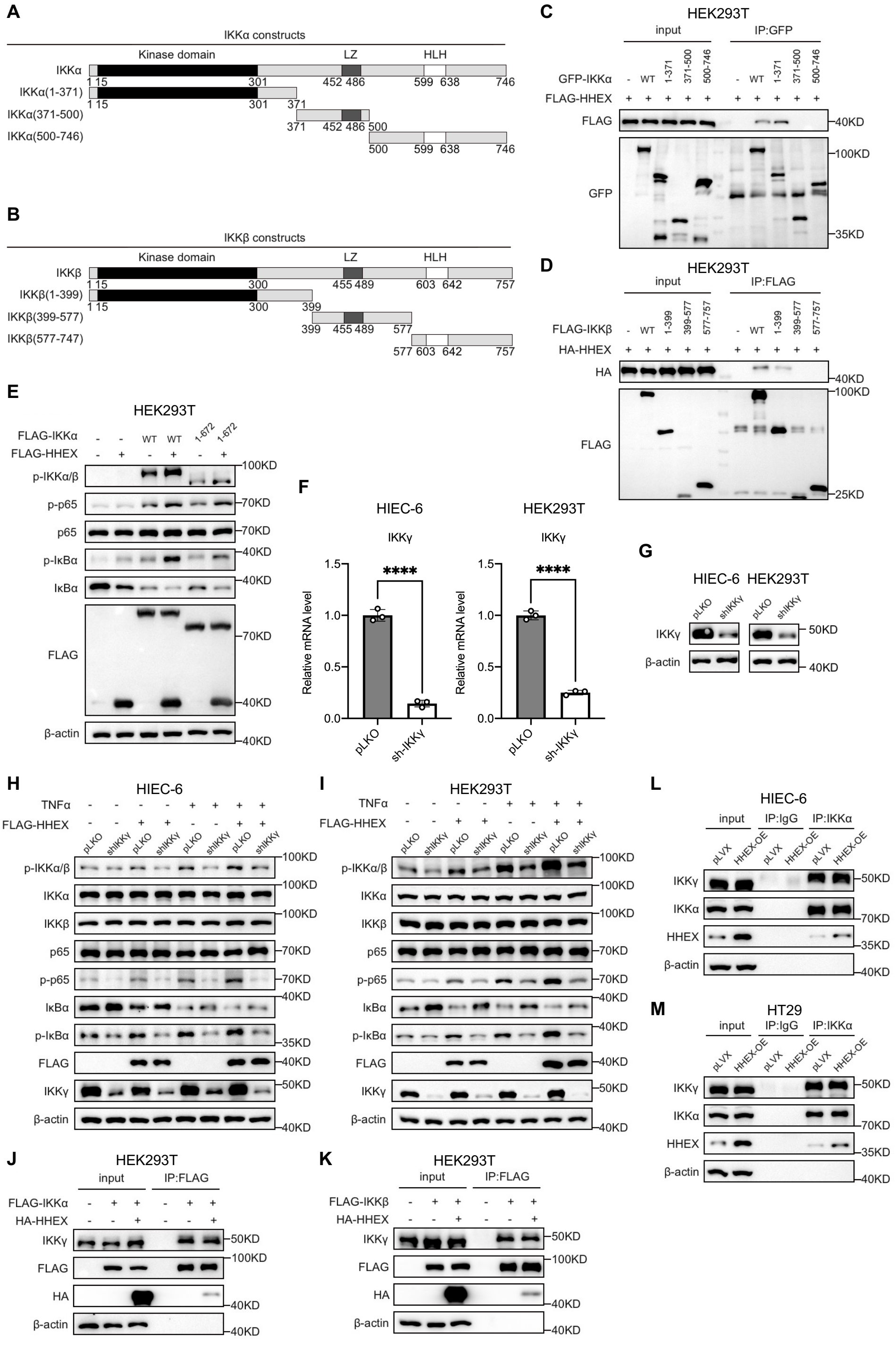

Supplementary Figure 4. HHEX may positively regulate IKKα/β complexes downstream of IKKγ independently of IKKγ.

(A-B) Schematic showing the protein structure of the full-length and truncated IKKα or IKKβ proteins. (C) Co-IP of exogenous full-length/truncated GFP-IKKα and FLAG-HHEX in HEK293T cells. (D) Co-IP of exogenous full-length/truncated FLAG-IKKβ and HA-HHEX in HEK293T cells. (E) Overexpression of HHEX and the IKKα full-

length/1-672 domain activated the NF-κB pathway in HEK293T cells. (F-G) The efficacy of IKKγ knockdown was examined in HIEC-6 and HEK293T cells via qRT–PCR (F) and Western blot analysis (G). (H-I) Western blot analysis of the activation status of the NF-κB pathway in control and IKKγ-knockdown HIEC-6(H) or HEK293T(I) cells with or without overexpression of FLAG-HHEX after TNF-α stimulation for 12h. (J-K) Semiendogenous co-IP of exogenous FLAG-IKKα(J) or FLAG-IKKβ(K) and endogenous IKKγ in HEK293T cells with or without HA-HHEX overexpression. (L-M) Endogenous co-IP of IKKα and IKKγ in HIEC-6(L) or HT29(M) cells with or without overexpression of HHEX. "pLKO" indicated the pLKO vector control for gene knockdown of IKKγ. The data are representative of 3 independent experiments in this figure. Two-tailed, unpaired Student's t test (F) was performed to assess statistical significance. * $P < 0.05$, ** $P < 0.01$, *** $P < 0.001$, **** $P < 0.0001$.

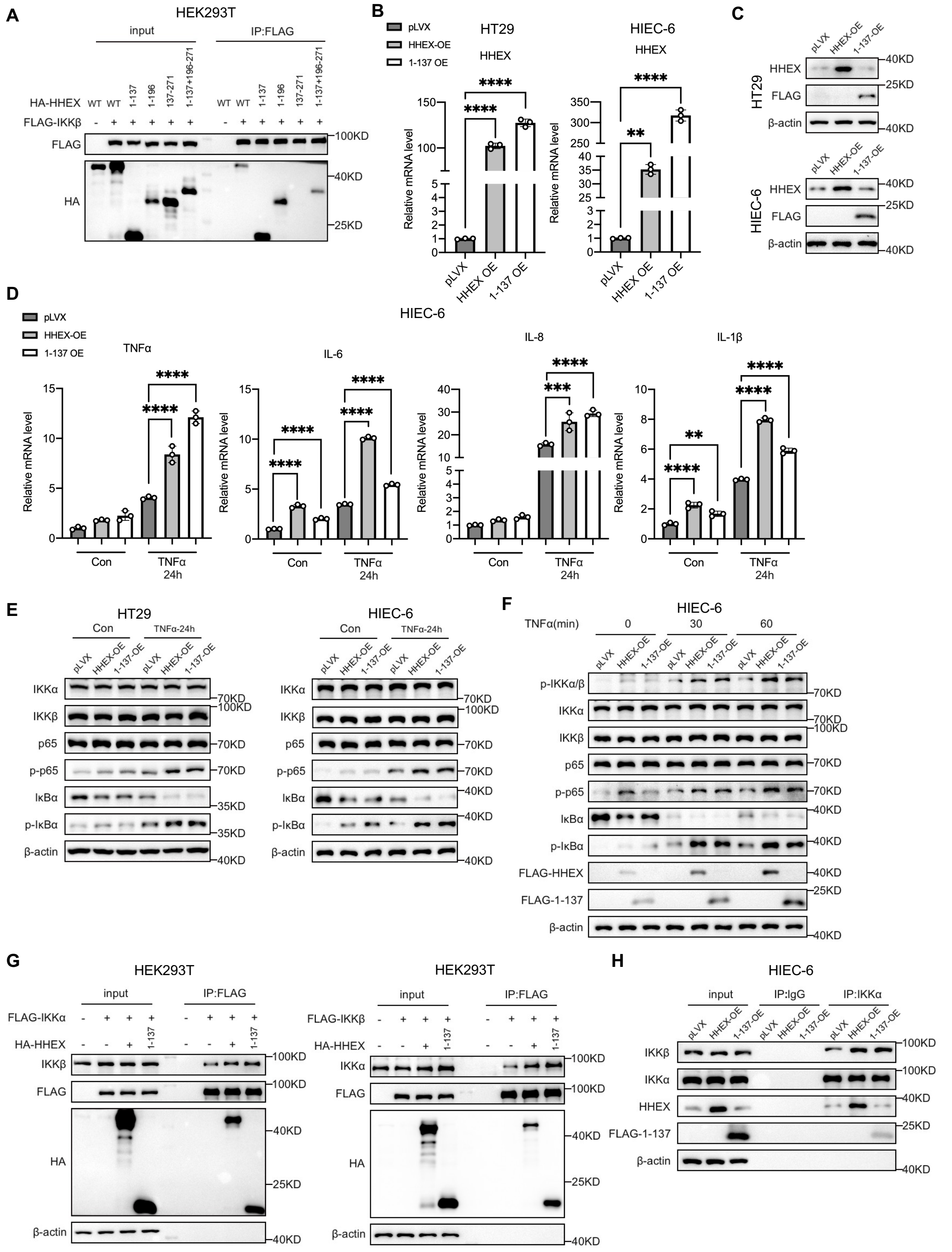

Supplementary Figure 5. The N-terminal domain is required for HHEX to interact with and activate the IKK complex.

(A) Co-IP of exogenous full-length/truncated HA-HHEX and FLAG-IKKβ in HEK293T cells. (B-C) The efficacy of HHEX and N-terminal domain overexpression was examined in HT29 and HIEC-6 cells by qRT–PCR (B) and Western blot analysis (C). (D) The mRNA levels of proinflammatory cytokines in HHEX and N-terminal domain-overexpressing and control HIEC-6 cells were measured via qRT–PCR. The

cells were treated with TNF-α (10 ng/ml) for the indicated times before qRT–PCR analysis. (E) Western blot analysis of the activation status of the NF-κB pathway in control, HHEX-overexpressing, and N-terminal domain-overexpressing HT29 and HIEC-6 cells after TNF-α stimulation for the indicated times. (F) Western blot analysis of the activation status of the NF-κB pathway in HIEC-6 cells with or without overexpression of HHEX and the N-terminal domain following short-term TNF-α stimulation. (G)Left panel: Semiendogenous co-IP of exogenous FLAG-IKKα and endogenous IKKβ in HEK293T cells with or without overexpression of HA-HHEX or HA-HHEX (1-137). Right panel: Semiendogenous co-IP of exogenous FLAG-IKKβ and endogenous IKKα in HEK293T cells with or without overexpression of HA-HHEX or HA-HHEX (1-137). (H) Endogenous co-IP of IKKα and IKKβ in HIEC-6 cells with or without overexpression of HHEX and the N-terminal domain. The data are presented as the means ± SDs and represent 3 independent experiments in this figure. One-way ANOVA with Tukey's multiple comparison test (B, D) was performed to assess statistical significance. * $P < 0.05$, ** $P < 0.01$, *** $P < 0.001$, **** $P < 0.0001$.

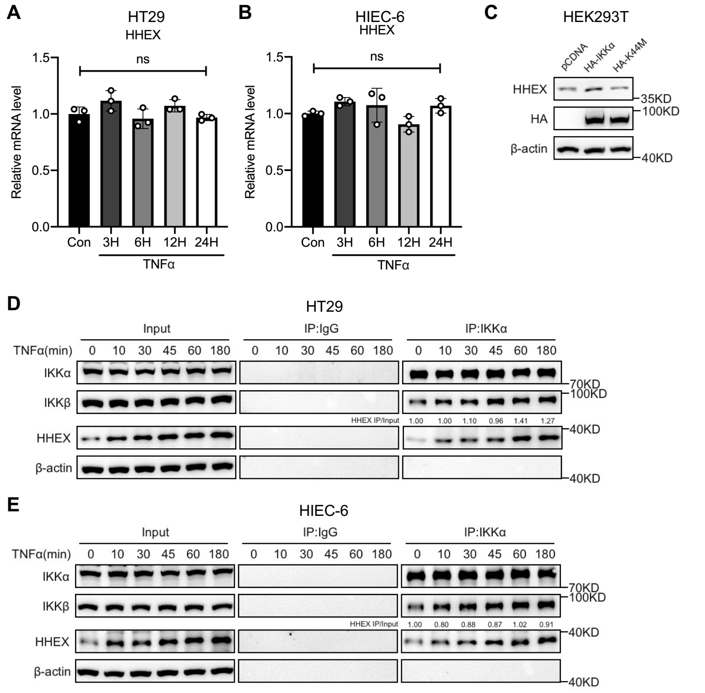

Supplementary Figure 6. The effect of TNF-α stimulation on HHEX expression and HHEX/IKK complex in intestinal epithelial cells.

(A-B) HHEX mRNA levels in HT29 (A) or HIEC-6 (B) cells were measured via qRT–PCR. The cells were treated with TNF-α (10 ng/ml) for the indicated times before qRT–PCR analysis. (C) Western blot analysis of HHEX expression in HEK293T cells with or without overexpression of IKKα^{WT} or IKKα^{K44M}. (D-E) Endogenous co-IP of IKKα and IKKβ/HHEX in HT29(D) or HIEC-6(E) cells following short-term TNF-α stimulation. The data are presented as the means ± SDs and represent 3 independent experiments in this figure. One-way ANOVA with Tukey's multiple comparison test (A, B) was performed to assess statistical significance.

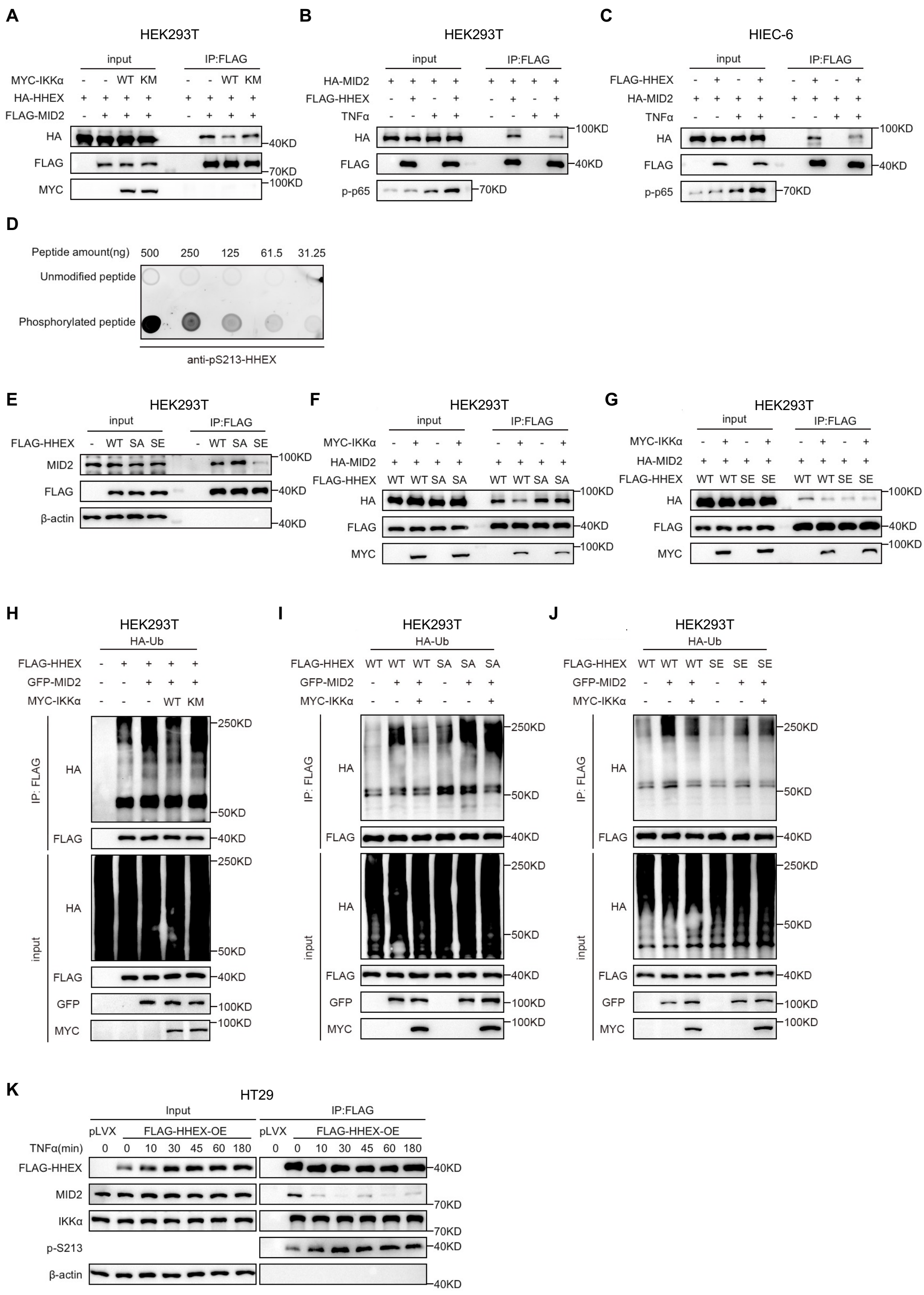

Supplementary Figure 7. IKKα-mediated HHEX S213 phosphorylation stabilizes HHEX via disrupting HHEX-MID2 interaction, and the effect of HHEX S213 mutation on its function in intestinal epithelial cells.

(A-C) The interaction between HHEX and MID2 was reduced under inflammatory conditions. Co-IP of exogenous HA-HHEX and FLAG-MID2 in HEK293T cells with or without overexpression of MYC-IKKα^{WT} or MYC-IKKα^{K44M} (A). Co-IP of

exogenous FLAG-HHEX and HA-MID2 with or without TNF-α (10 ng/ml) stimulation for 24 hours in HEK293T cells (B) or HIEC-6 cells (C). (D) Efficacy detection of S213 phosphorylation-specific antibodies via the dot blot method with different amounts of phosphorylated and unmodified peptides. (E) Semiendogenous co-IP of exogenous FLAG-HHEX^{WT}, FLAG-HHEX^{S213A} or FLAG-HHEX^{S213E} and endogenous MID2 in HEK293T cells. (F-G) Co-IP of exogenous HA-MID2 and FLAG-HHEX^{WT} or FLAG-HHEX^{S213A}/FLAG-HHEX^{S213E} in HEK293T cells with or without overexpression of MYC-IKKα. (H-J) IKKα^{WT} reduced the level of HHEX^{WT} ubiquitination mediated by MID2 but not that of HHEX^{S213A} or the HHEX^{S213E} mutant. Western blot analysis of the level of HHEX ubiquitination in HEK293T cells with or without overexpression of GFP-MID2 and MYC-IKKα^{WT} or MYC-IKKα^{K44M} (H). Western blot analysis of the levels of HHEX and HHEX^{S213A} mutant (I) or HHEX^{S213E} mutant (J) ubiquitination with or without overexpression of GFP-MID2 and MYC-IKKα in HEK293T cells. (K) Semiendogenous co-IP of exogenous FLAG-HHEX with endogenous MID2 and IKKα, and Western blot analysis of S213 phosphorylation levels in control and FLAG-HHEX-overexpressing HT29 cells following short-term TNF-α stimulation. The data are representative of 3 independent experiments in this figure.

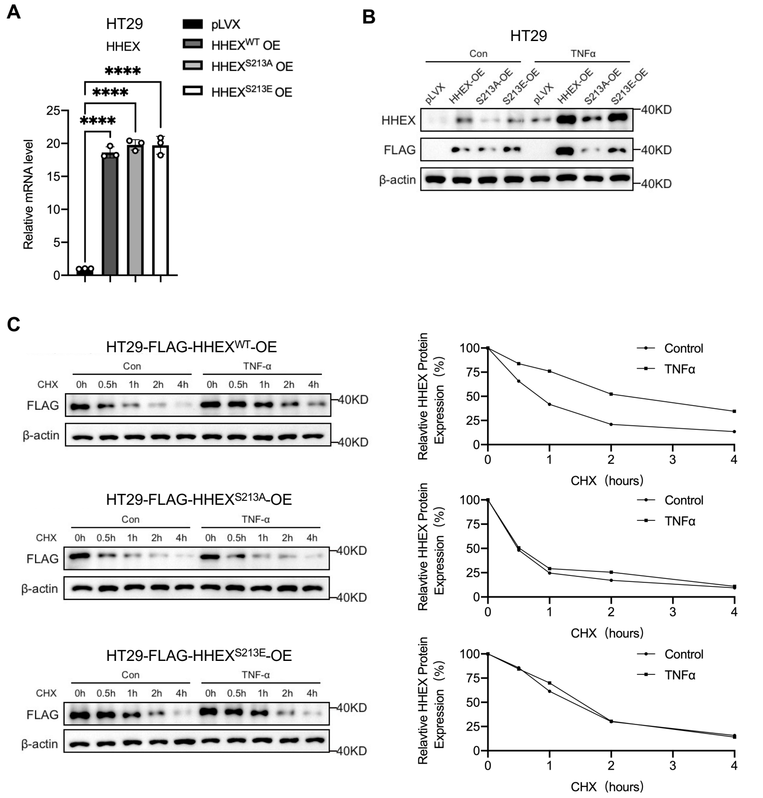

Supplementary Figure 8. The effect of S213 site mutation on the function of HHEX in intestinal epithelial cells.

(A) The efficacy of $HHEX^{WT}$, $HHEX^{S213A}$ and $HHEX^{S213E}$ overexpression was examined in HT29 cells by qRT–PCR. (B) Western blot analysis of $HHEX^{WT}$, $HHEX^{S213A}$ and $HHEX^{S213E}$ expression in HT29 cells after TNF-α stimulation for 12 hours. (C) Effects of TNF-α stimulation on the half-life of $HHEX^{WT}$, $HHEX^{S213A}$ or $HHEX^{S213E}$ in HT29 cells. The cells were pretreated with or without TNF-α (10 ng/ml) for 16 hours and then treated with cycloheximide (CHX) (75 μg/ml) for the indicated times before Western blot analysis. The data are presented as the means ± SDs and represent 3 independent experiments in this figure. One-way ANOVA with Tukey's multiple comparison test (A) was performed to assess statistical significance. **** $P < 0.0001$.

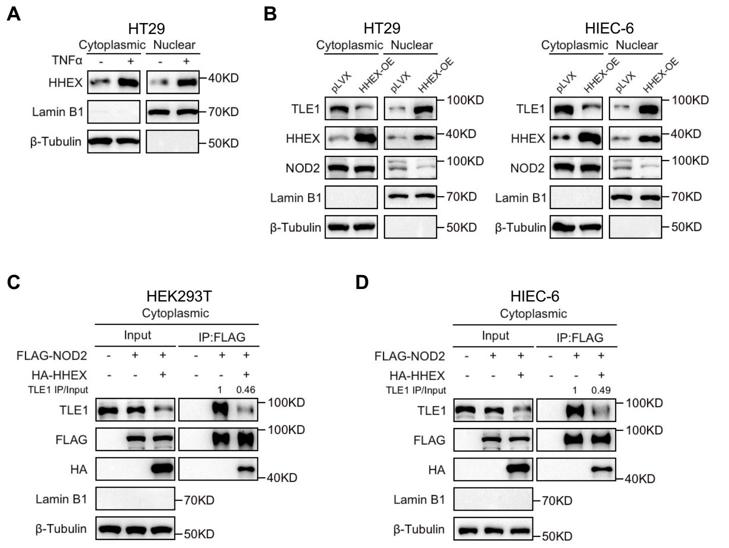

Supplementary Figure 9. Overexpression of HHEX diminishes the interaction between NOD2 and TLE1 in cytoplasm.

(A) Western blot analysis of HHEX expression in the cytoplasm and nucleus of HT29 cells following 12h of TNF-α stimulation. (B) Western blot analysis of TLE1 and NOD2 expression in the cytoplasmic and nuclear fractions of HT29 and HIEC-6 cells with or without overexpression of HHEX. (C-D) Exogenous co-IP of the effect of HHEX overexpression on the cytoplasmic interaction between TLE1 and NOD2 in HEK293T(C) and HIEC-6(D) cells. The data are representative of 3 independent experiments in this figure.

Supplementary Table 1: Sequences for real-time PCR used in this study.

Gene	Sequence
HHEX (human)	Forward: AGCTCTCAATGTTCGCCCTC
	Reverse: TCGCCCTCAATGTCCACTTC
TNF-α (human)	Forward: CCTCTCTCTAATCAGCCCTCTG
	Reverse: GAGGACCTGGGAGTAGATGAG
IL-6 (human)	Forward: ACTCACCTCTTCAGAACGAATTG
	Reverse: CCATCTTTGGAAGGTTCAGGTTG
IL-1β (human)	Forward: ATGATGGCTTATTACAGTGGCAA
	Reverse: GTCGGAGATTCGTAGCTGGA
IL-8 (human)	Forward: ACTCCAAACCTTTCCACC
	Reverse: CTTCTCCACAACCCTCTG
IKKγ (human)	Forward: AGAATACGACAACCACATCA
	Reverse: AACGGTCTCCATCACAATC
β-actin (human)	Forward: CATGTACGTTGCTATCCAGGC
	Reverse: CTCCTTAATGTCACGCACGAT
Hhex (mouse)	Forward: TCAGAATCGCCGAGCTAAAT
	Reverse: CTGTCCAACGCATCCTTTTT
TNF-α (mouse)	Forward: CAGGCGGTGCCTATGTCTC
	Reverse: CGATCACCCCGAAGTTCAGTAG
IL-6 (mouse)	Forward: TAGTCCTTCCTACCCCAATTTCC
	Reverse: TTGGTCCTTAGCCACTCCTTC
IL-1β (mouse)	Forward: GAAATGCCACCTTTTGACAGTG
	Reverse: TGGATGCTCTCATCAGGACAG
IL-8 (mouse)	Forward: TCGAGACCATTTACTGCAACAG
	Reverse: CATTGCCGGTGGAAATTCCTT
GAPDH (mouse)	Forward: AGGTCGGTGTGAACGGATTTG
	Reverse: GGGGTCGTTGATGGCAACA
CXCL12 (human)	Forward: ATTCTCAACACTCCAAACTGTGC
	Reverse: ACTTTAGCTTCGGGTCAATGC
CXCL13 (human)	Forward: GCTTGAGGTGTAGATGTGTCC
	Reverse: CCCACGGGGCAAGATTTGAA
Gp2(mouse)	Forward: GCTCAGTTGGCCTCTCAGAA
	Reverse: CTGCTACCTCGAAGGGGACT
SpiB(mouse)	Forward: CAGCTGTCCAGGTCGTAGAAG
	Reverse: AACCACCATGCTTGCTCTG
