# Supplementary material for: An HHEX/IKKα positive feedback loop promotes intestinal inflammation
Source: J Clin Invest. 2026 Mar 17;136(10):e192074. doi: 10.1172/JCI192074 (PMC13178647; doi:10.1172/JCI192074)

Figure 1A

HHEX

β-actin

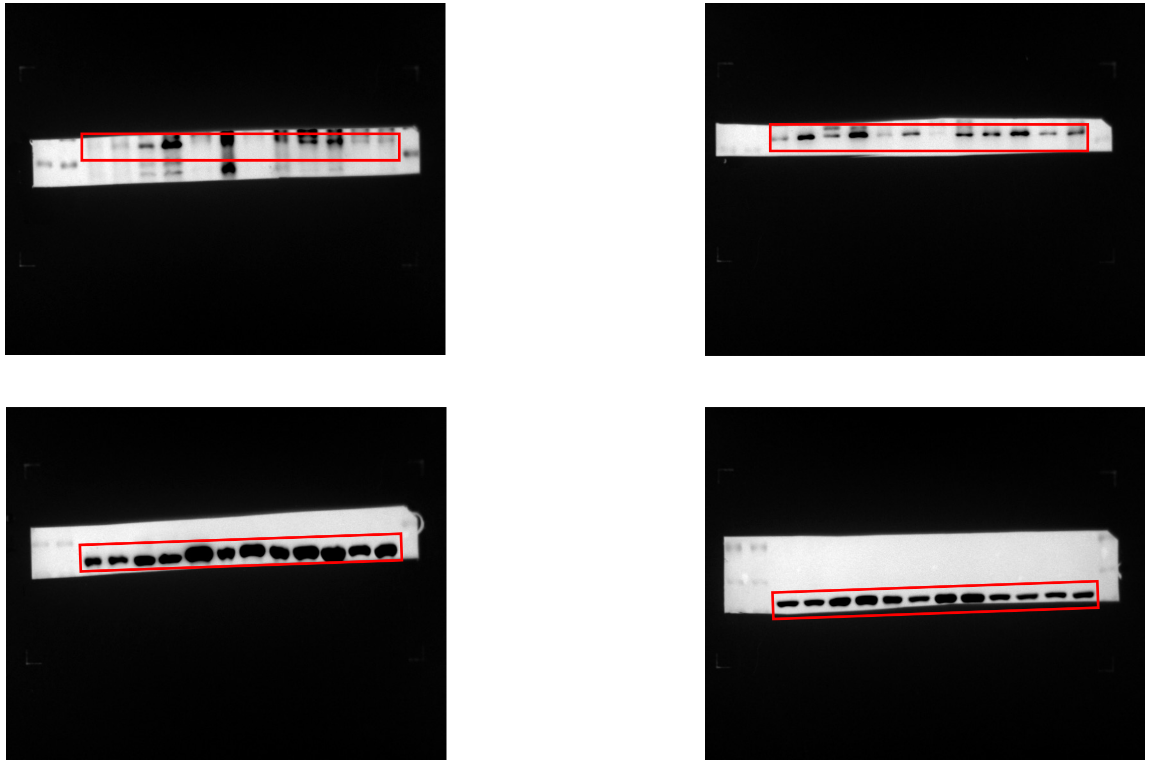

Figure 2C

FLAG-IKKβ

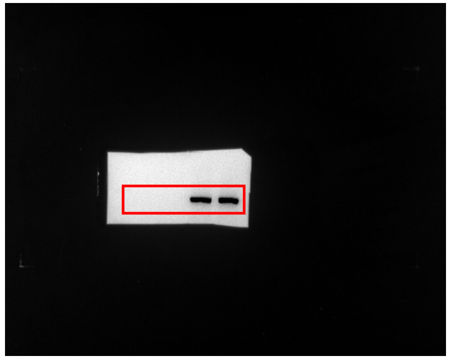

p-IKKα/β

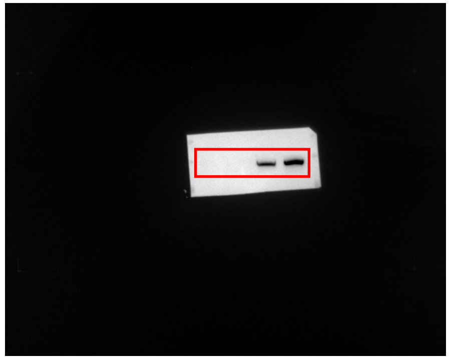

IκBα

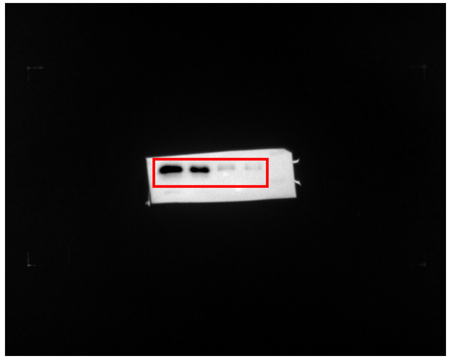

HA

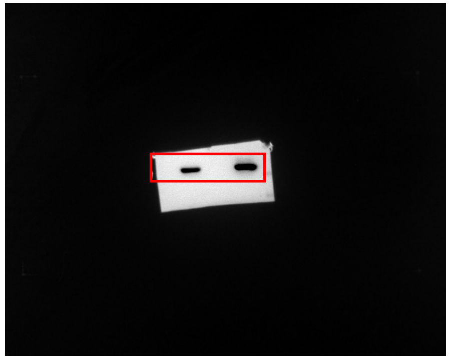

p65

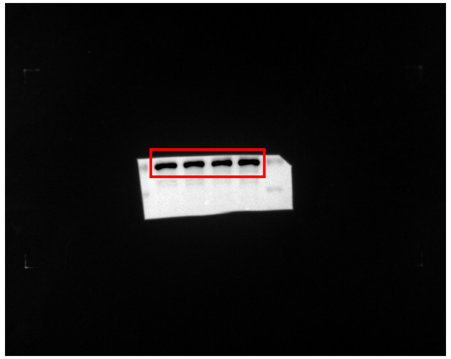

p-IκBα

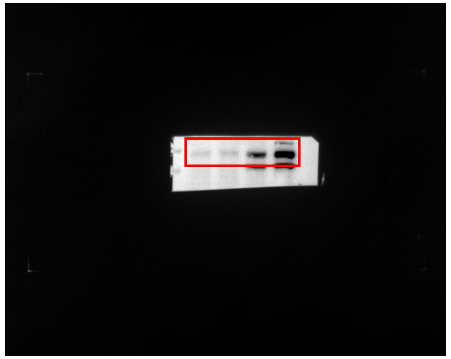

β-actin

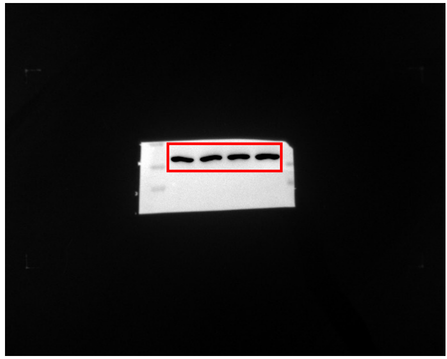

IKKα

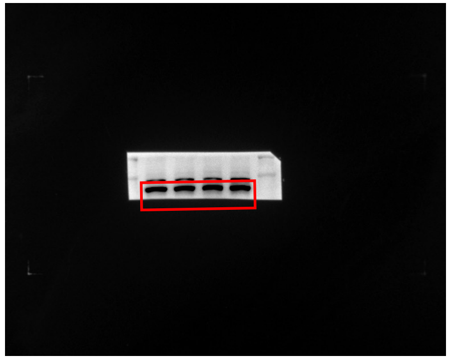

p-p65

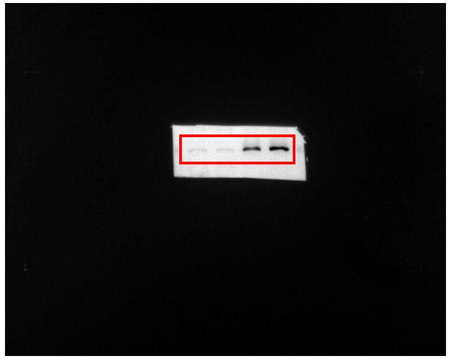

FLAG-GAPDH

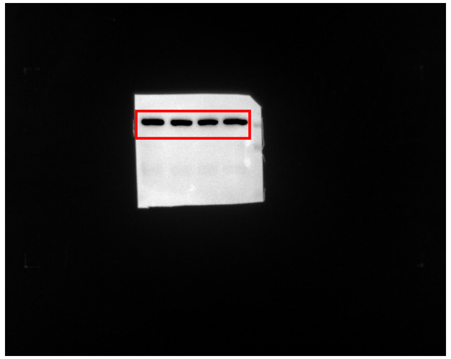

Figure 2D

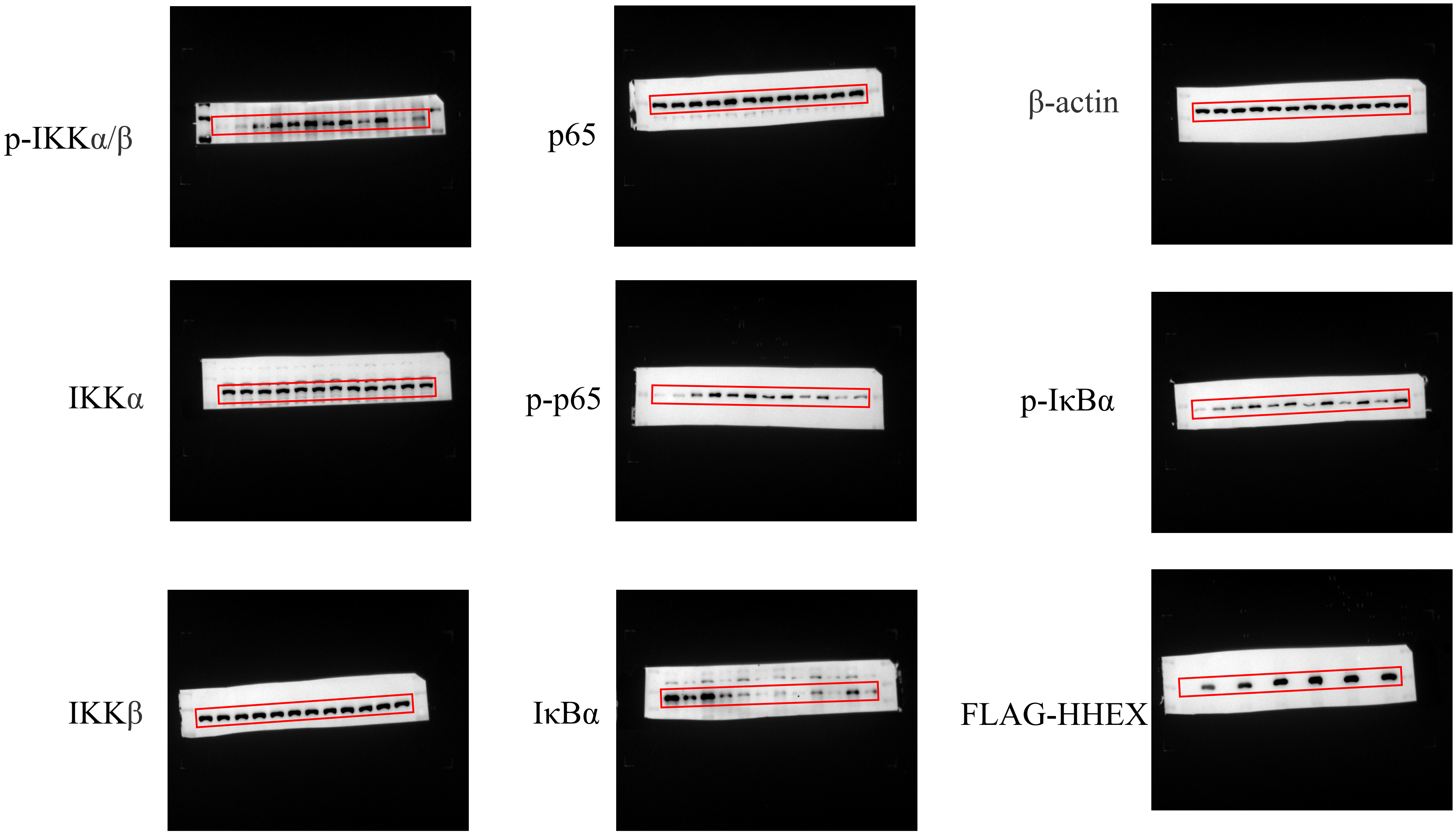

Figure 2E

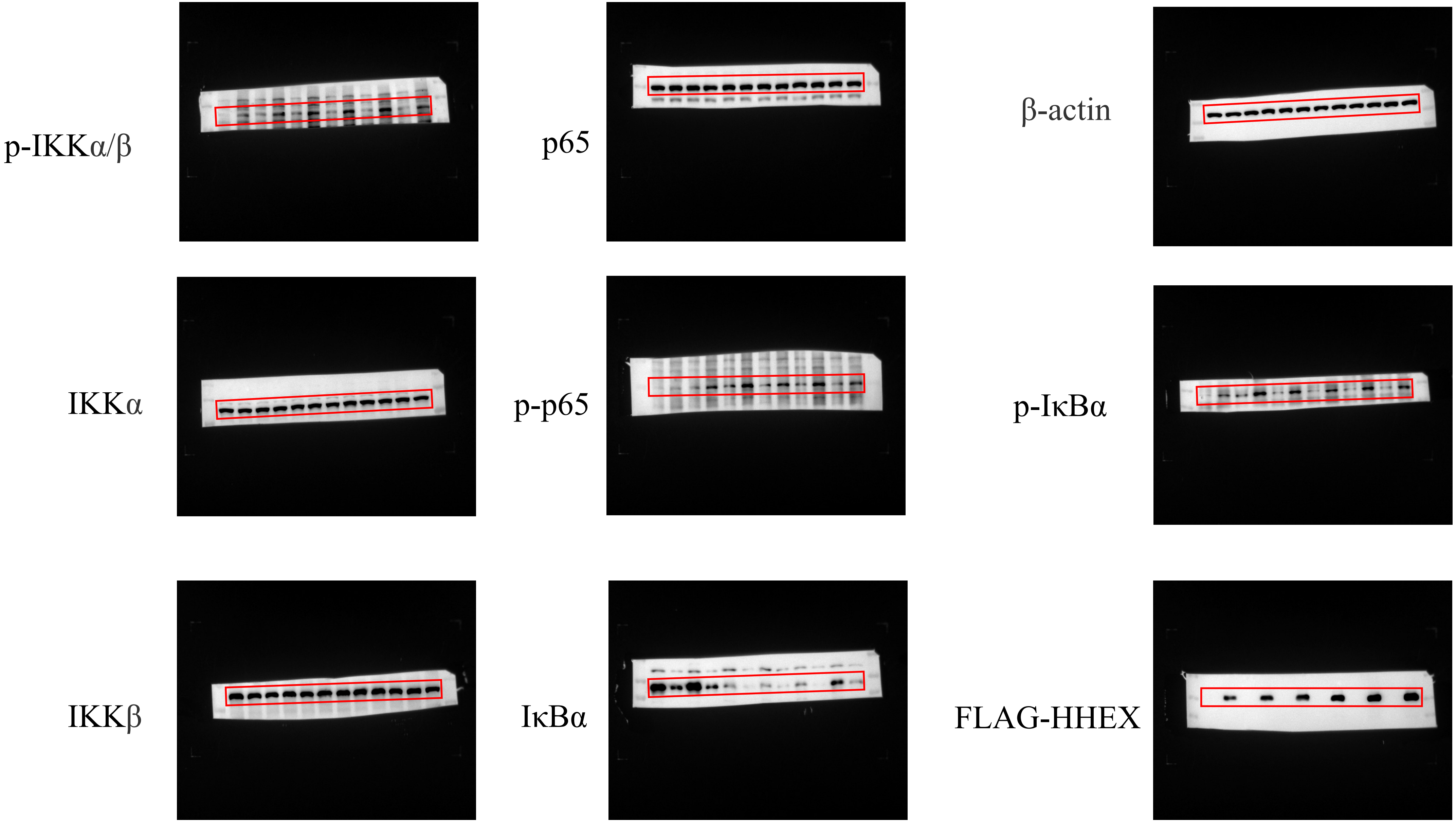

Figure 2G

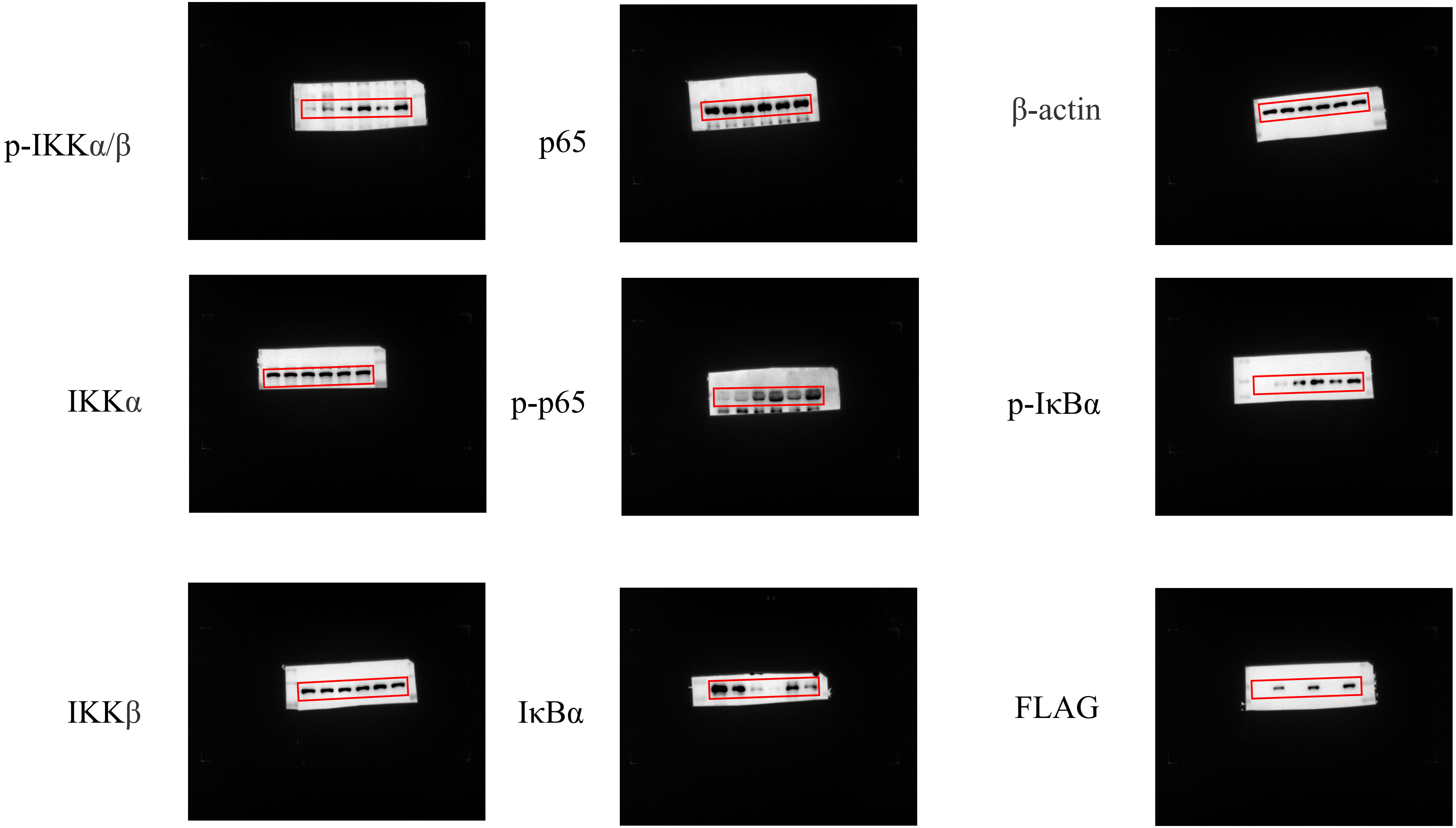

Figure 3A

HHEX

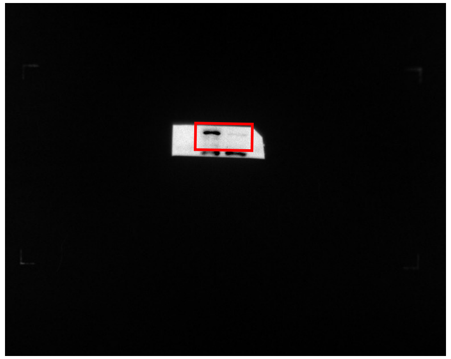

β-actin

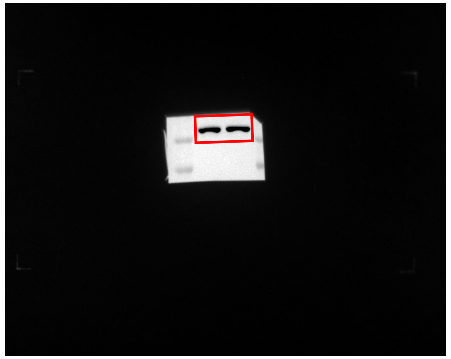

Figure 3G

p-IKKα/β

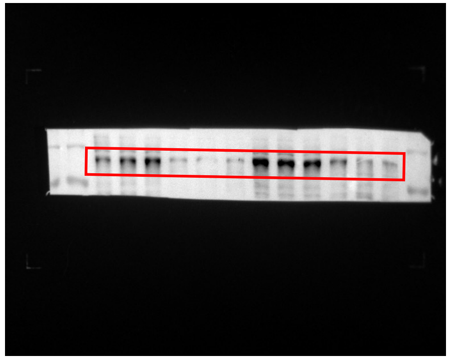

p65

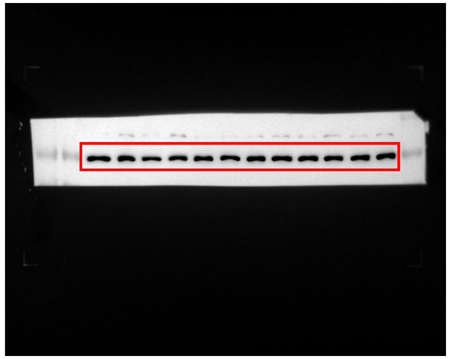

IKKα

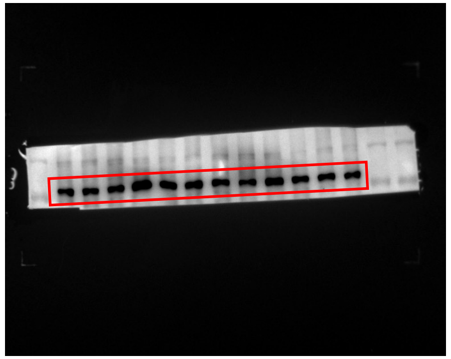

p-p65

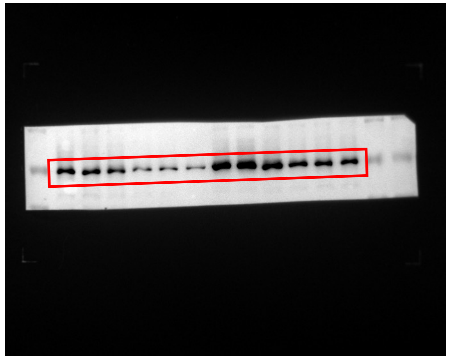

p-IκBα

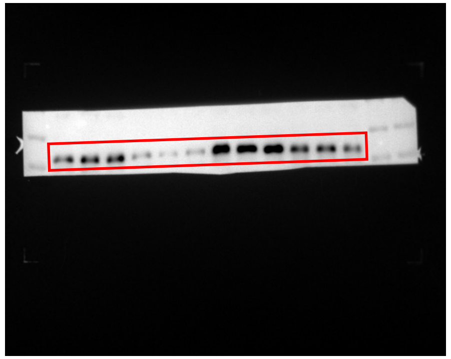

IKKβ

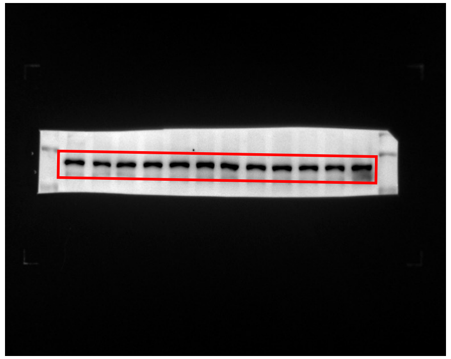

IκBα

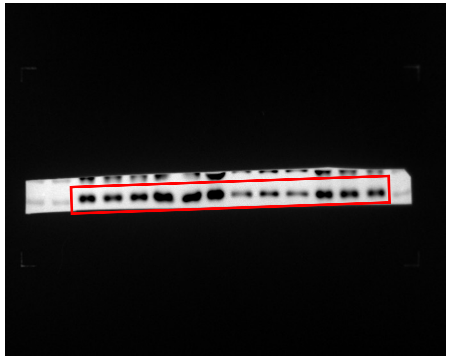

β-actin

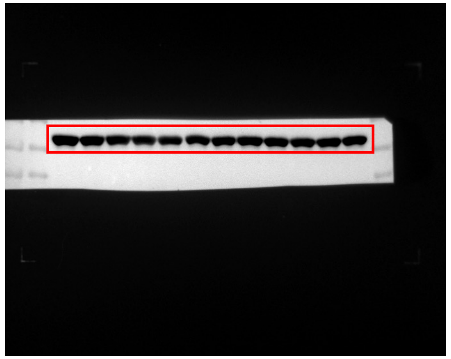

Figure 4 A

HT29 HIEC-6

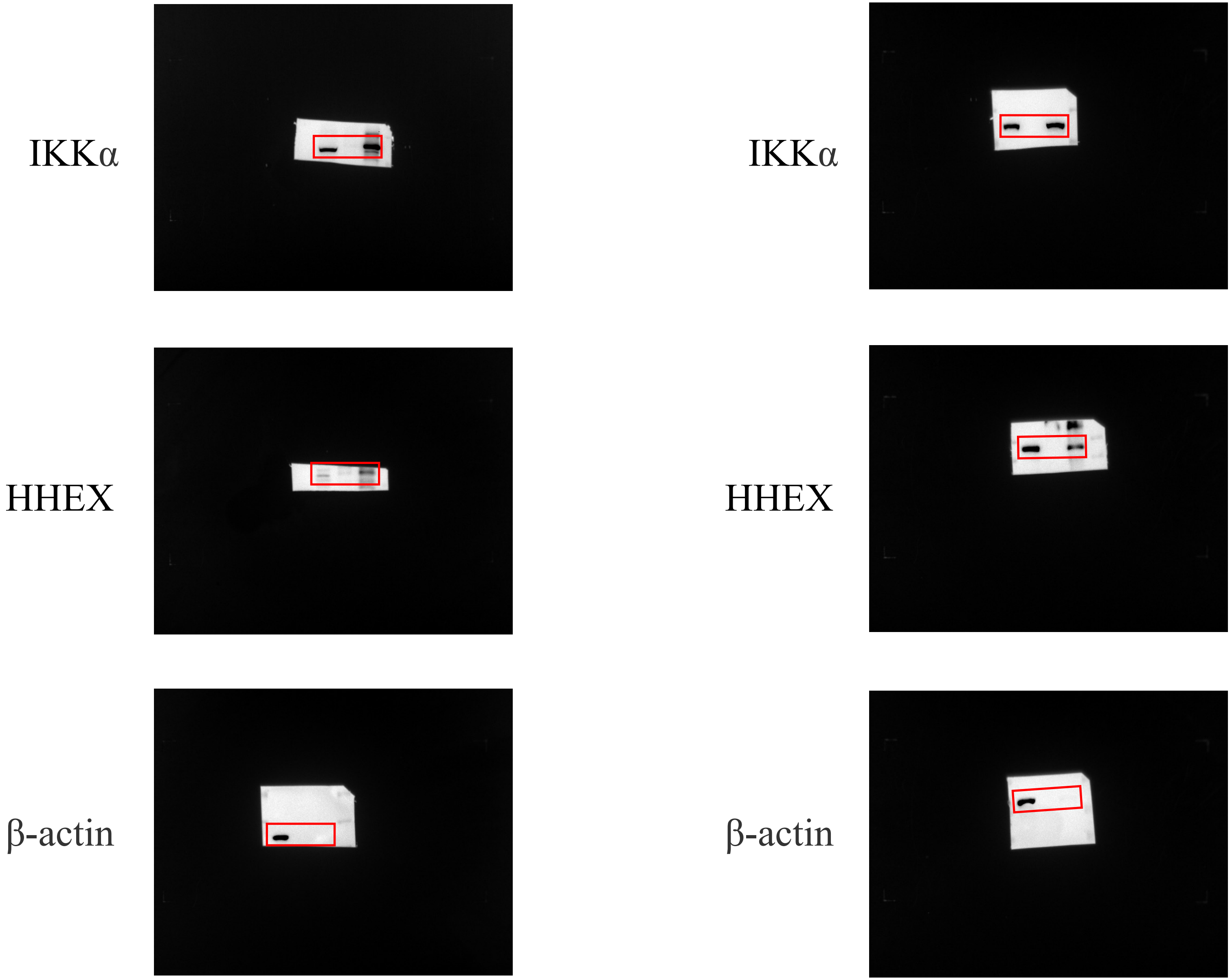

Figure 4B

IKKβ

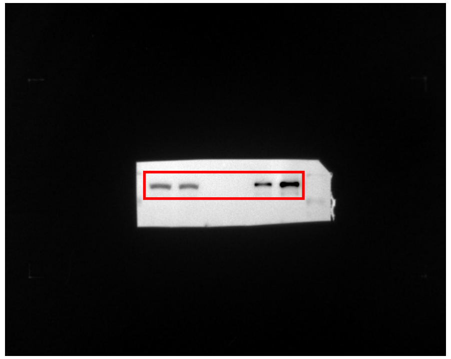

β-actin

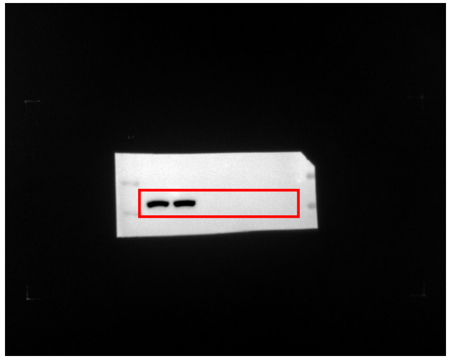

IKKα

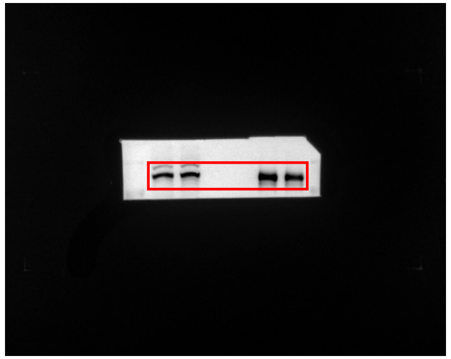

HHEX

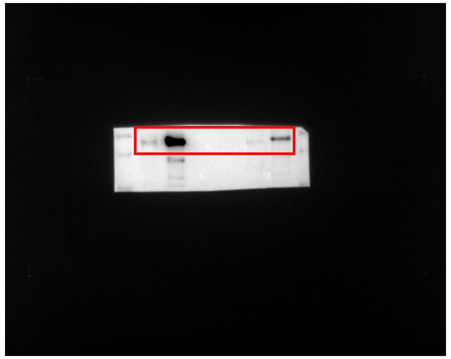

Figure 4C

IKKβ

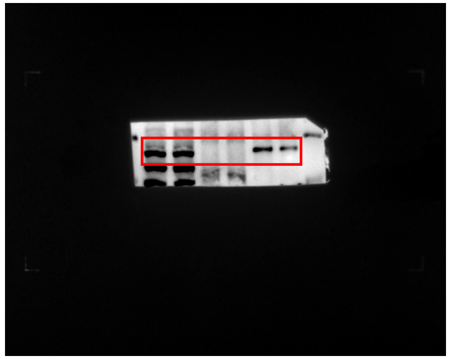

β-actin

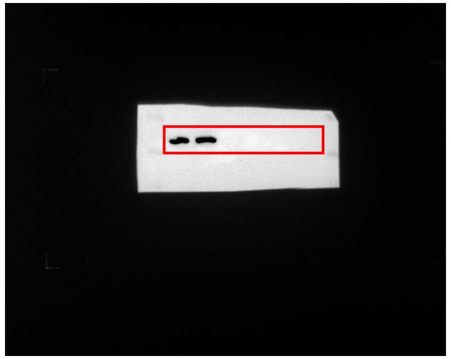

IKKα

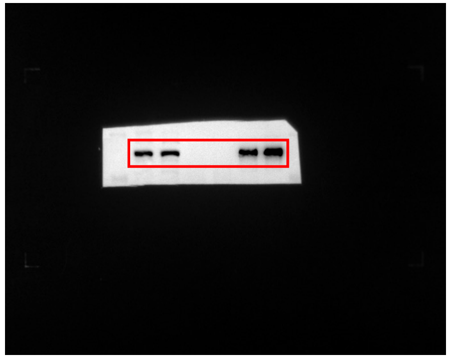

HHEX

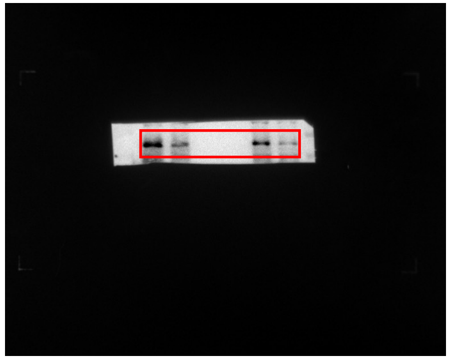

Figure 4D

IKKβ

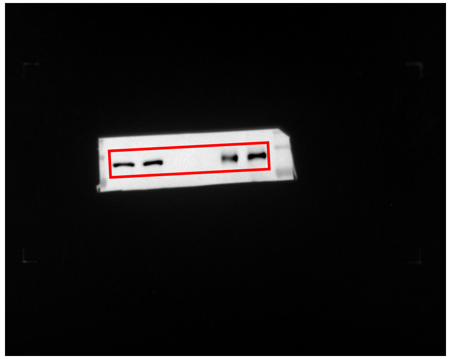

β-actin

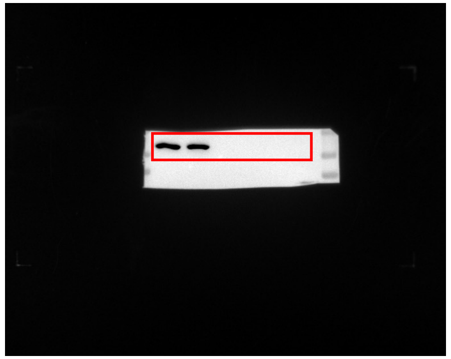

IKKα

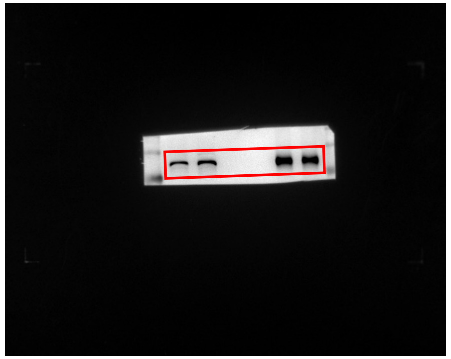

FLAG

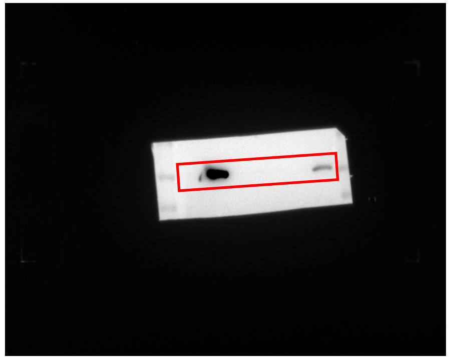

Figure 4F

FLAG

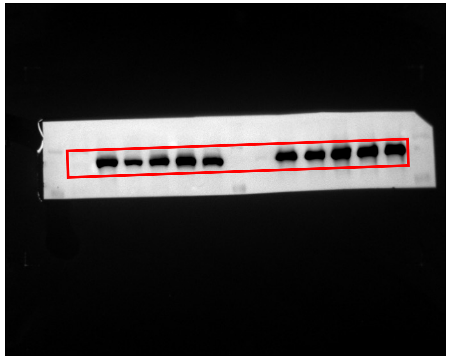

HA

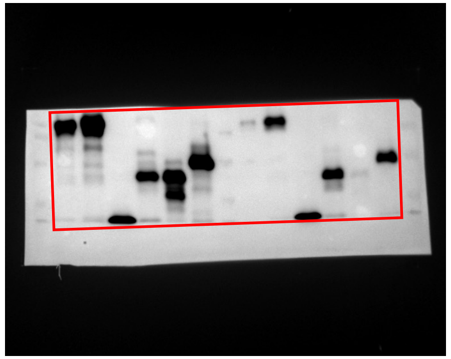

Figure 4H

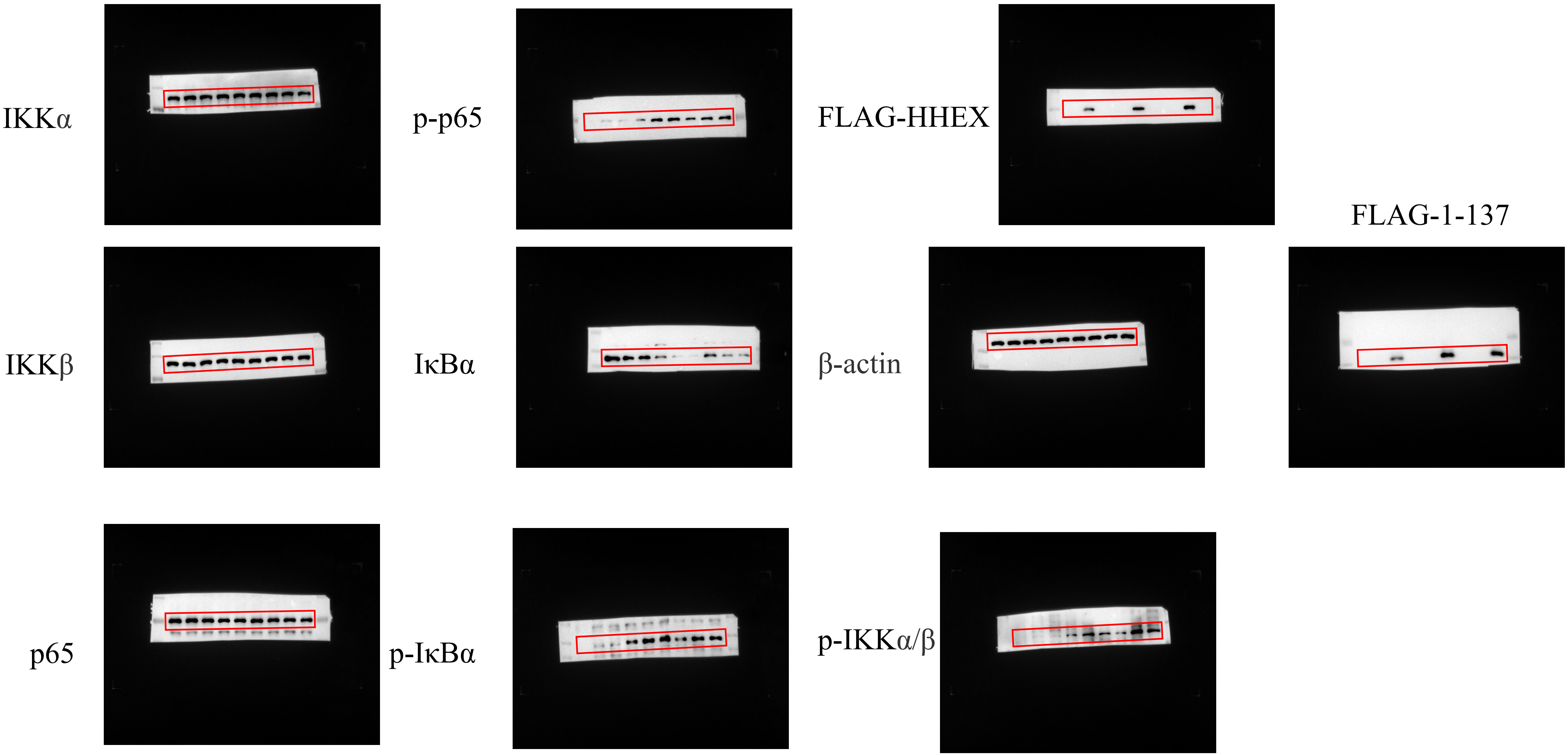

IKKα
p-p65
FLAG-HHEX
FLAG-1-137
IKKβ
IκBα
β-actin
p65
p-IκBα
p-IKKα/β

Figure 4I

IKKβ

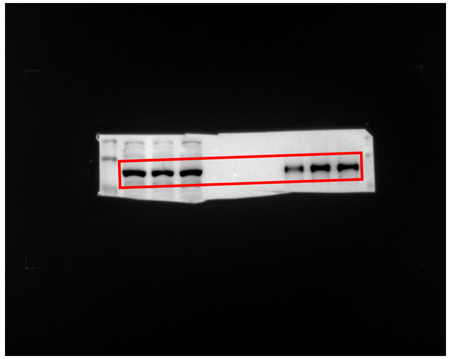

FLAG

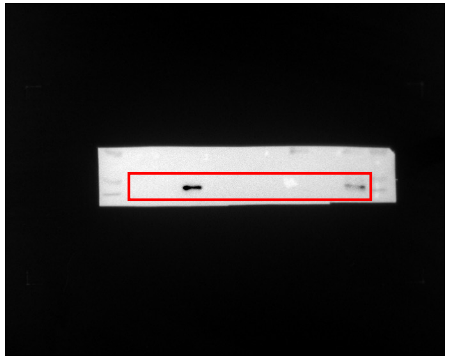

IKKα

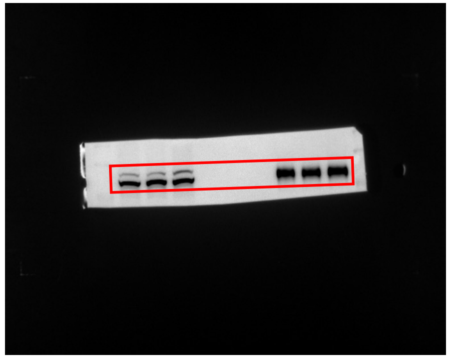

β-actin

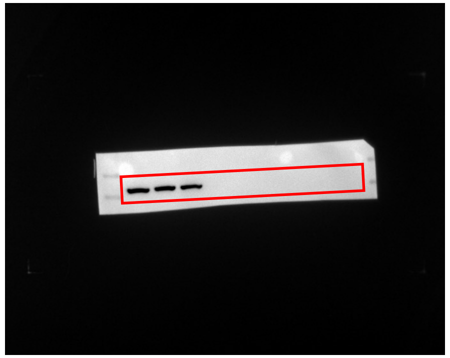

HHEX

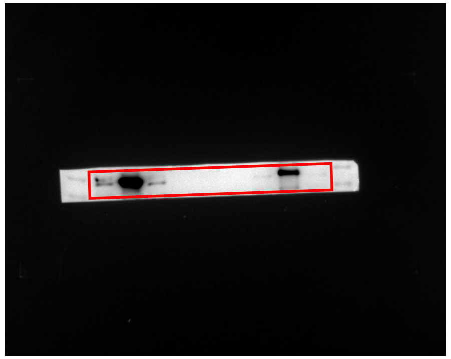

Figure 5A

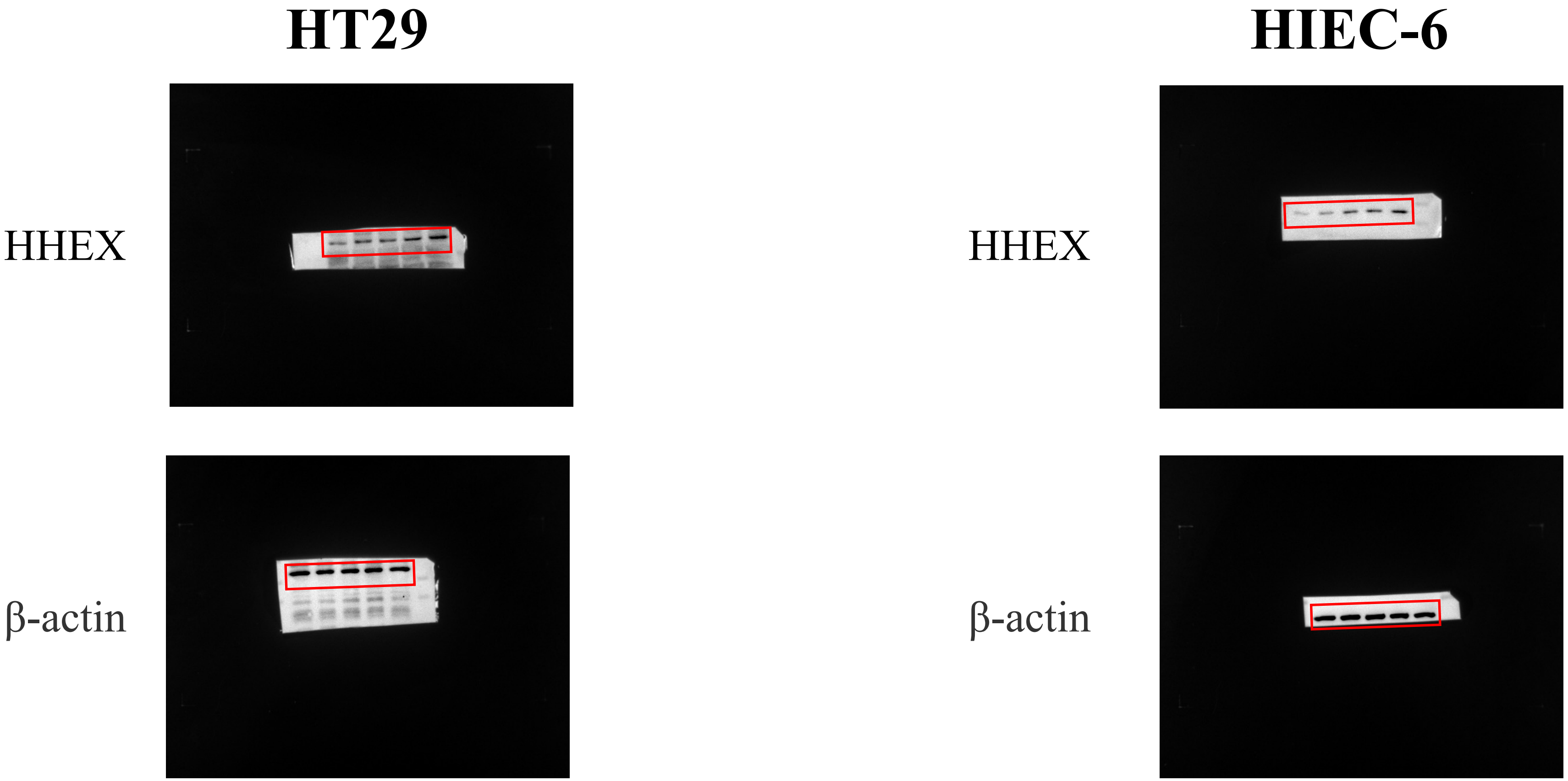

Figure 5B

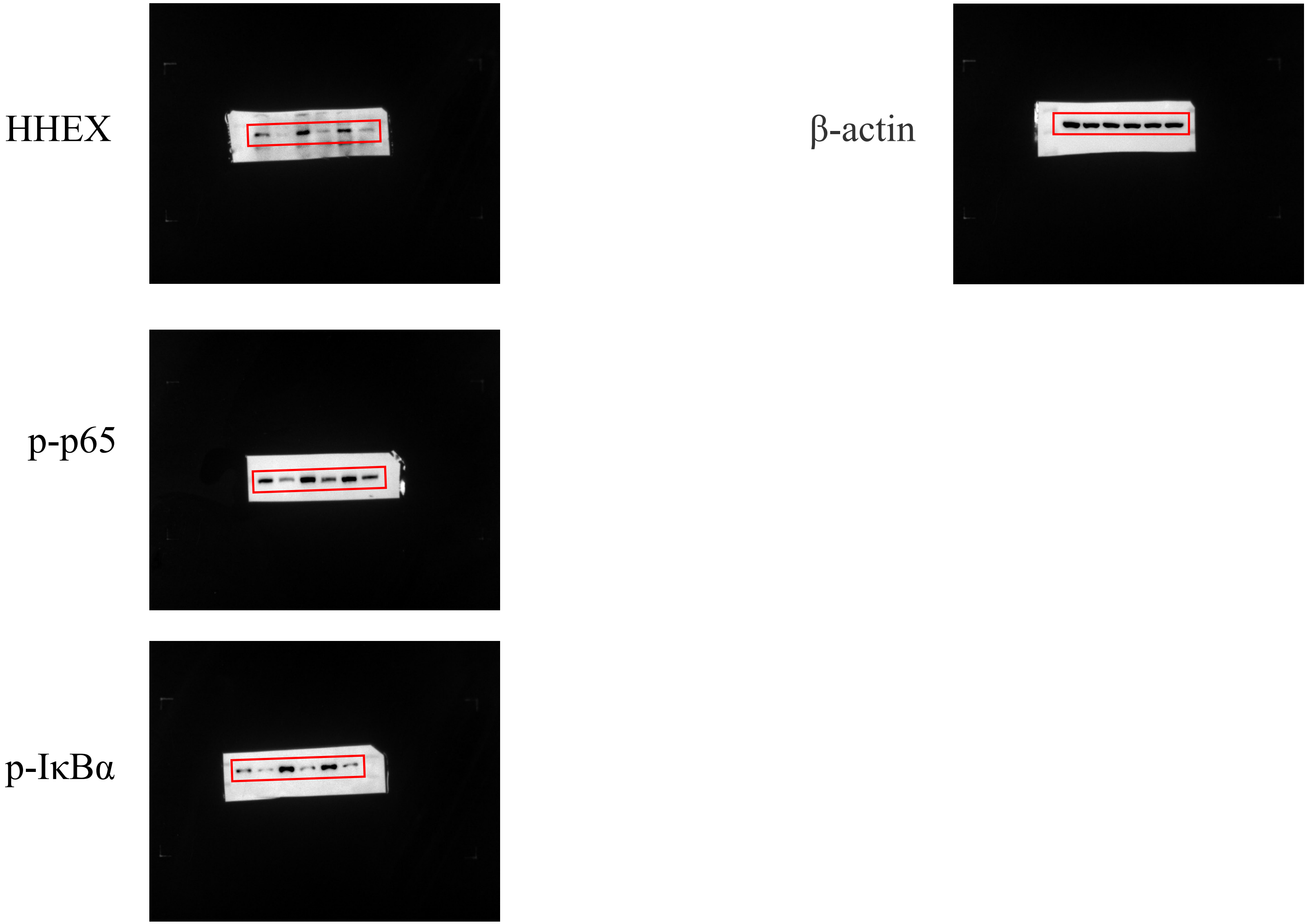

Figure 5C

HHEX

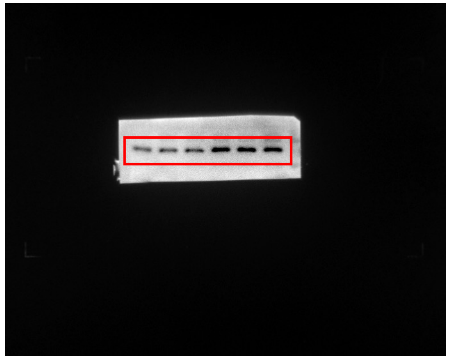

β-actin

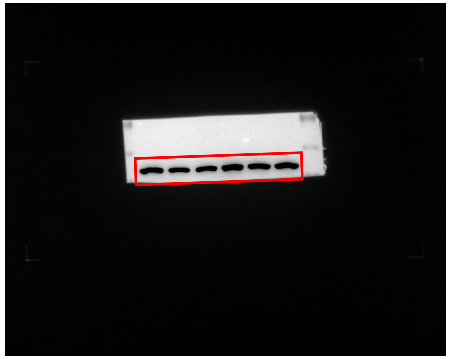

Figure 5F

HHEX

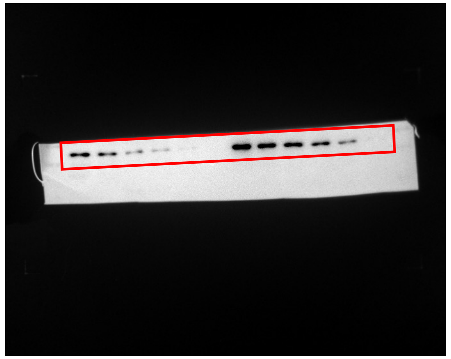

β-actin

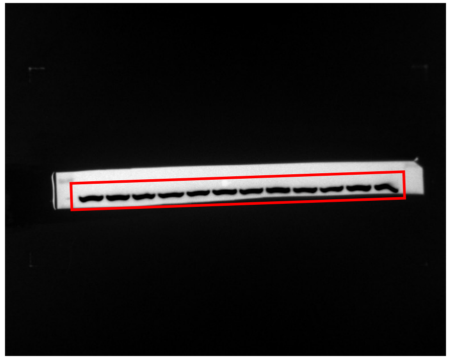

Figure 5G

HHEX

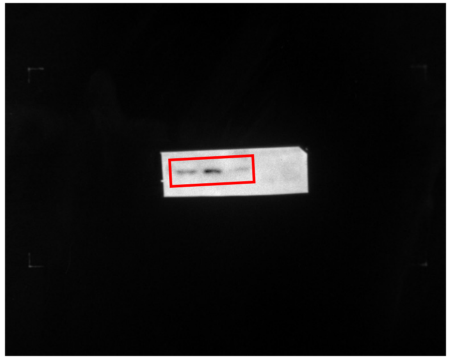

β-actin

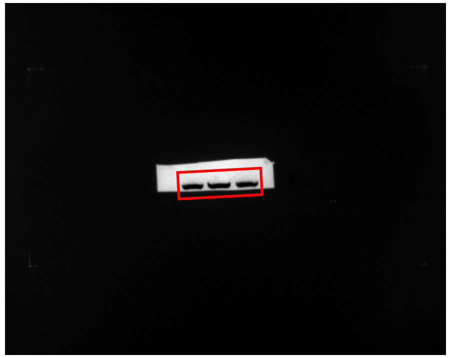

Figure 5H

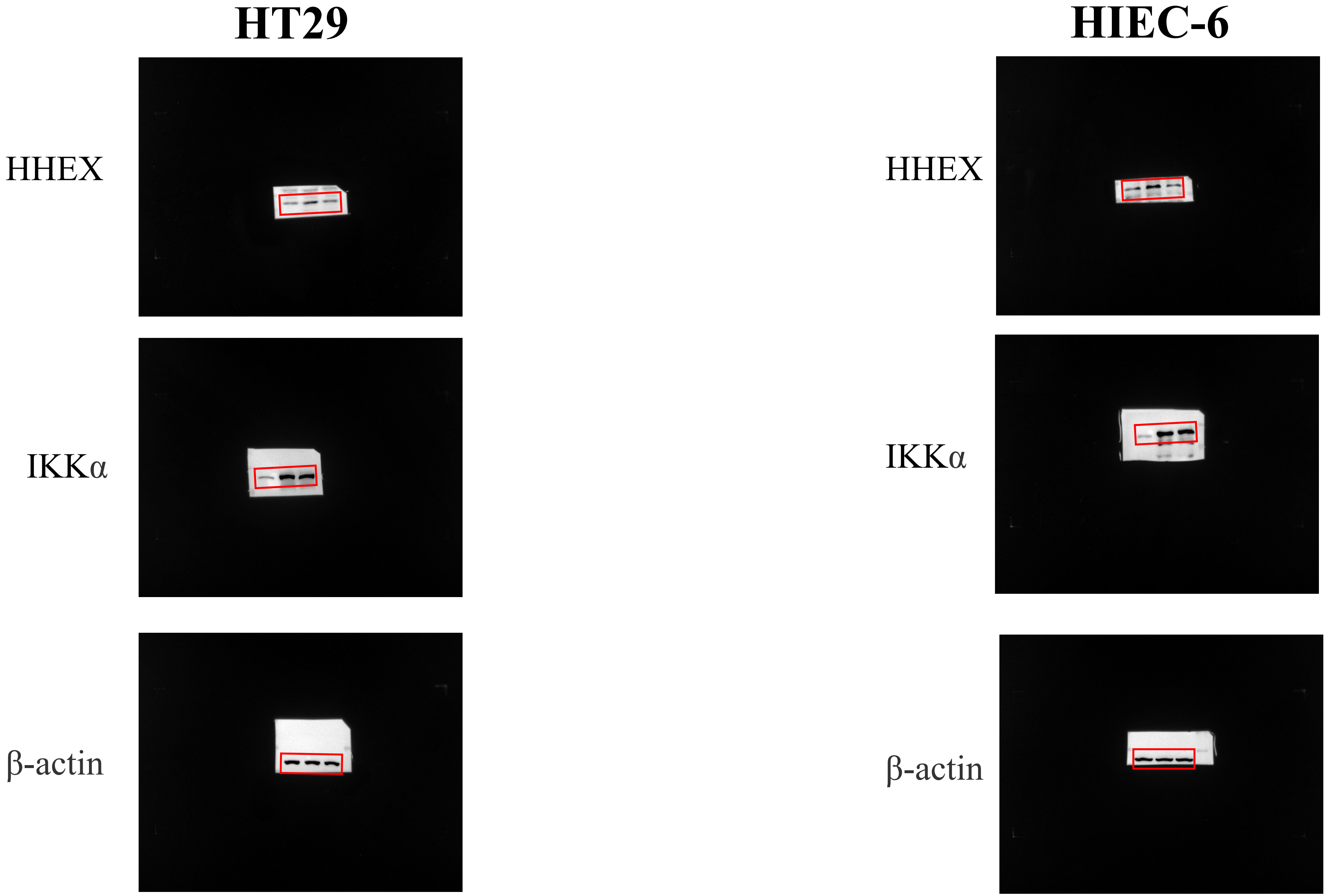

Figure 5I

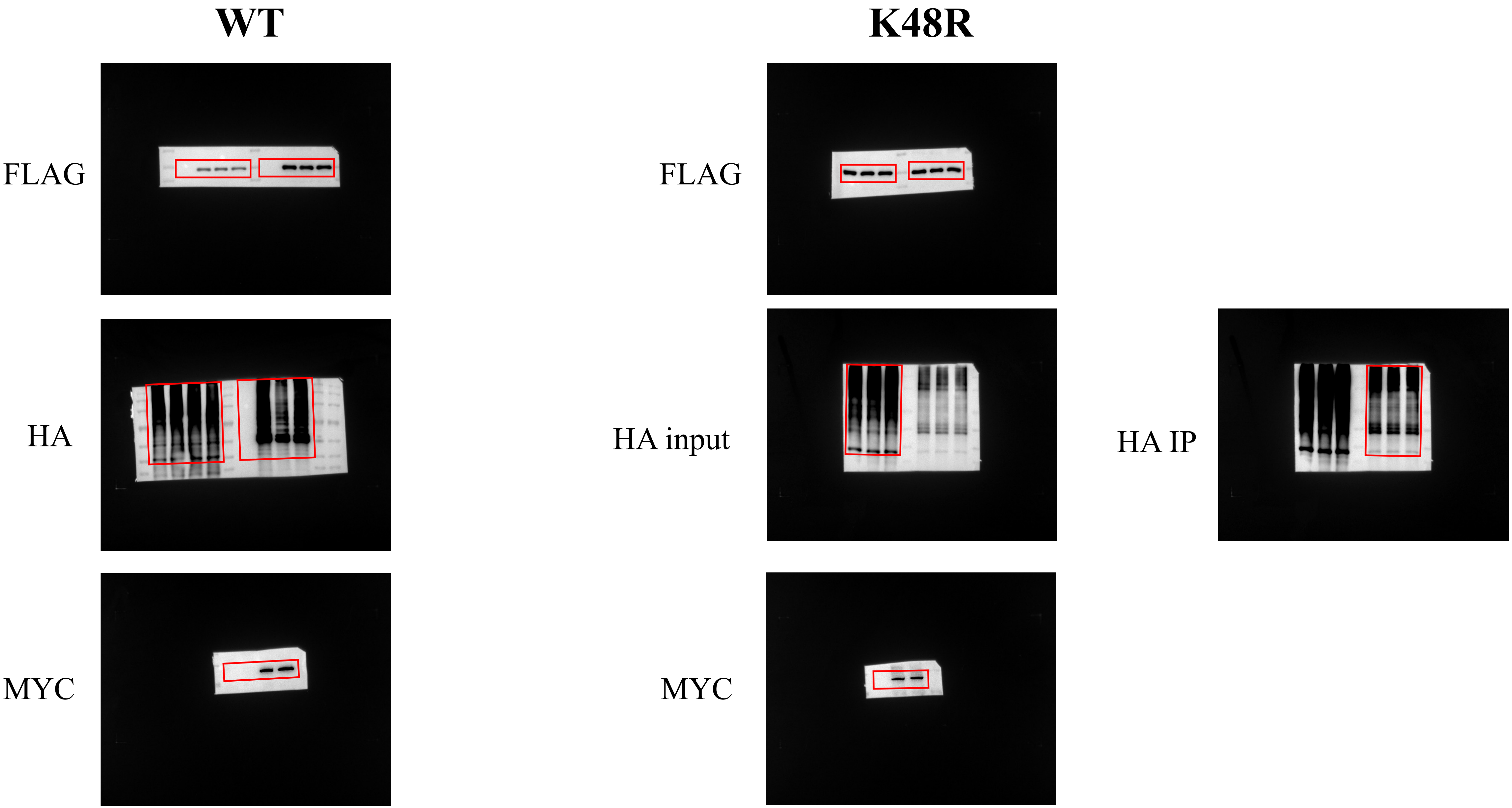

Figure 6A

HA

FLAG

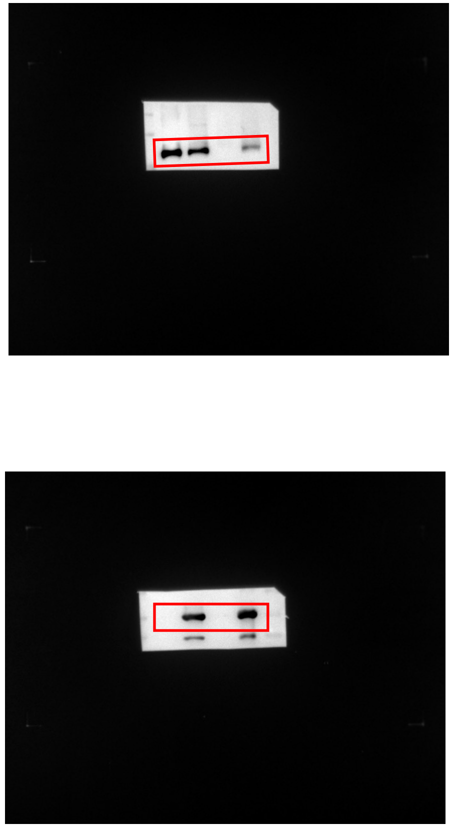

Figure 6B

HA

FLAG

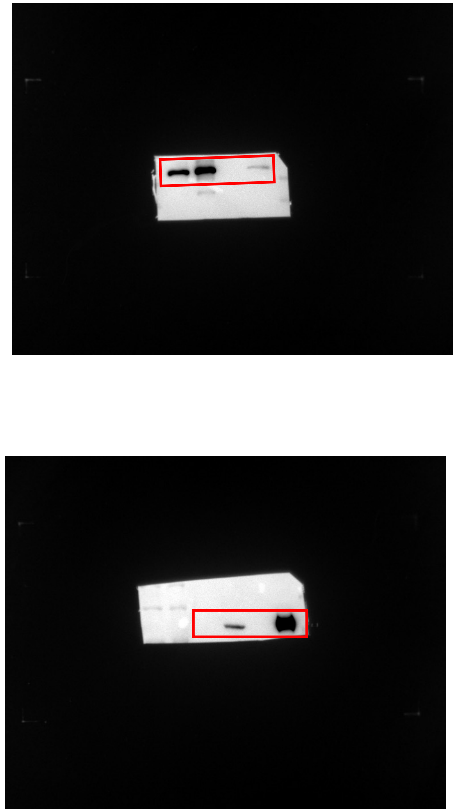

Figure 6C

MID2

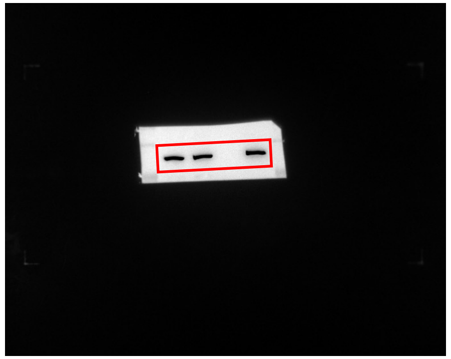

FLAG

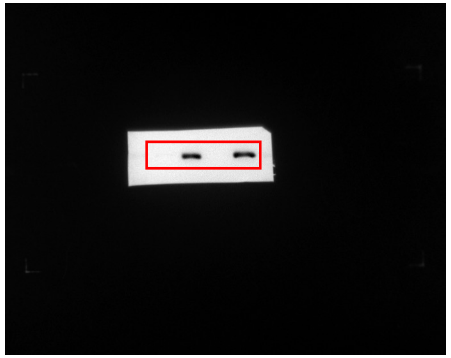

β-actin

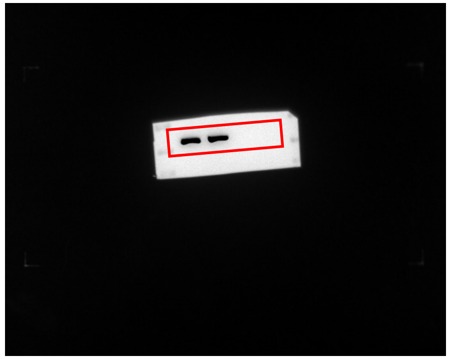

Figure 6D

MID2

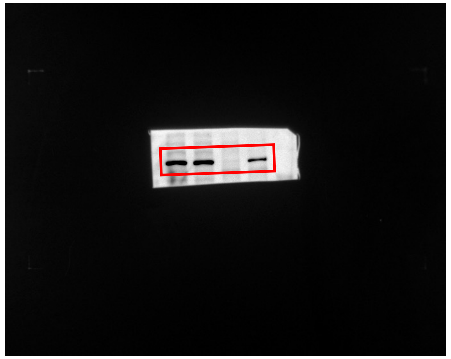

FLAG

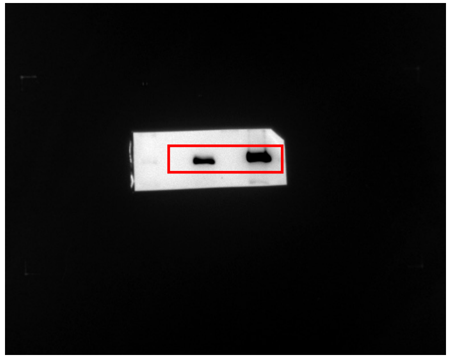

β-actin

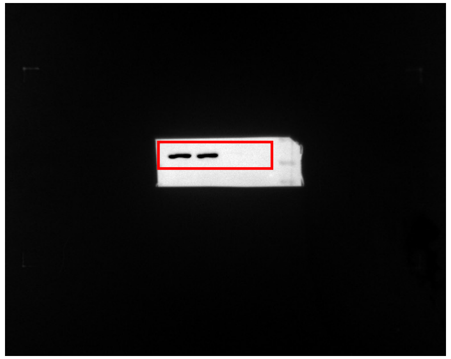

Figure 6E

HHEX

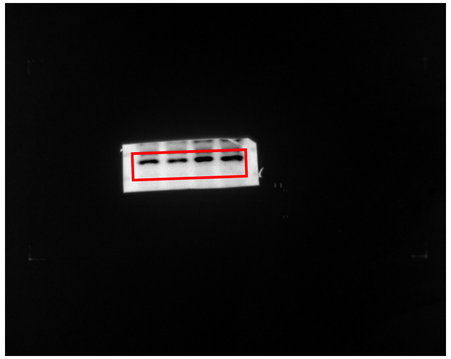

FLAG

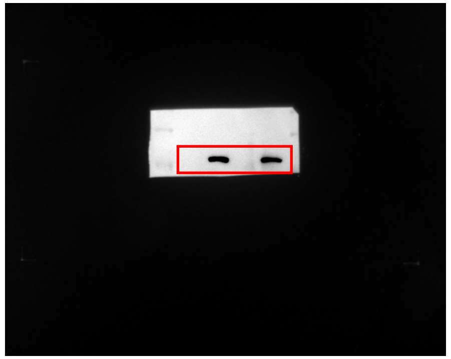

β-actin

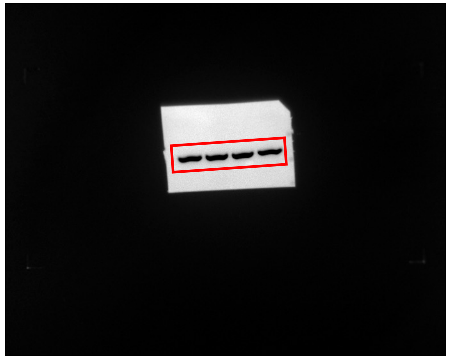

Figure 6F HIEC-6

HHEX

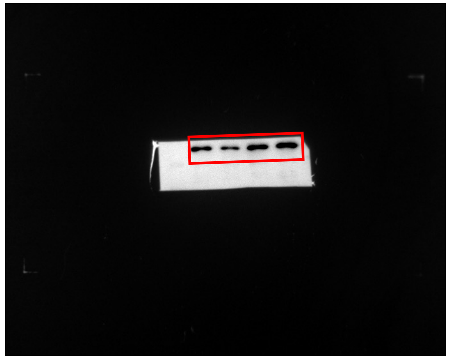

FLAG

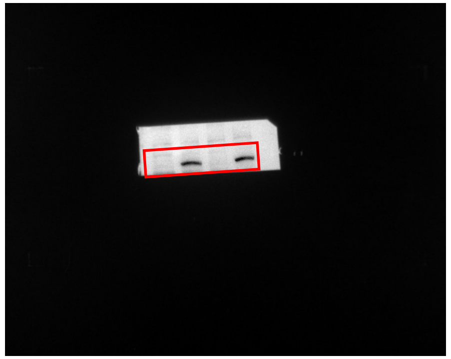

β-actin

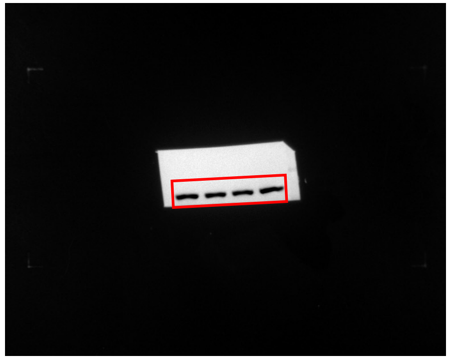

Figure 6F HT29

HHEX

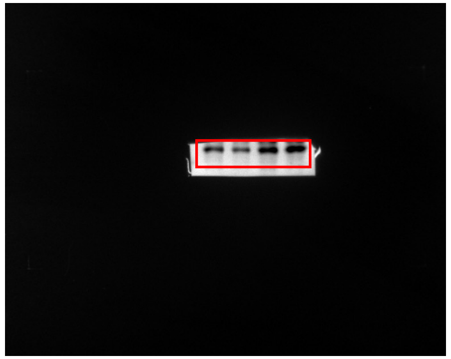

FLAG

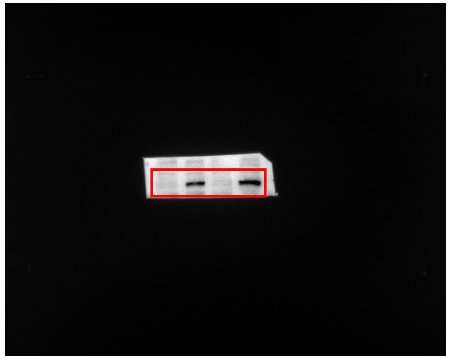

β-actin

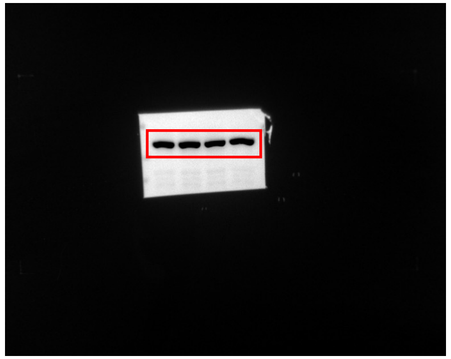

Figure 6G

HHEX

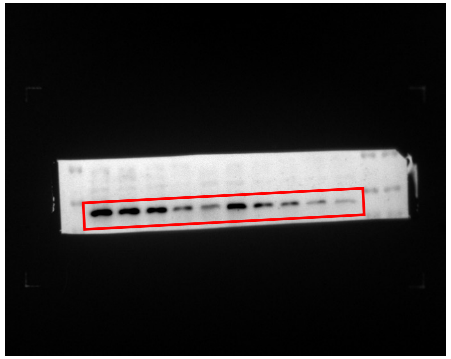

FLAG

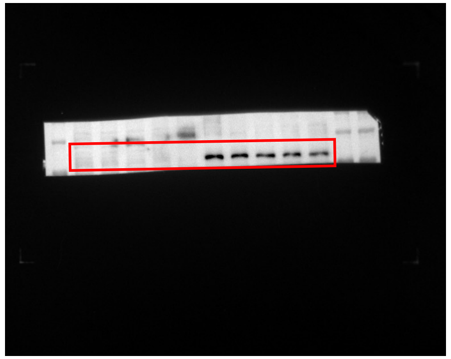

β-actin

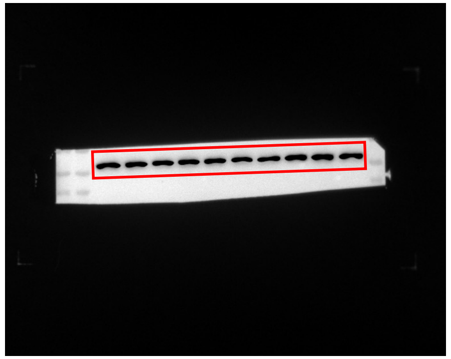

Figure 6H

HHEX

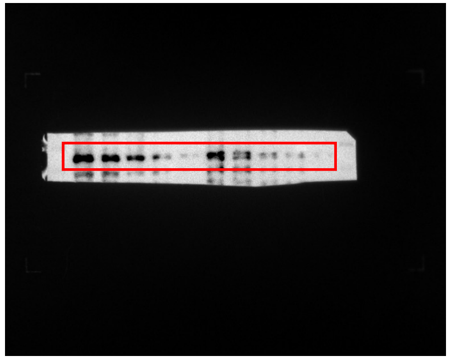

FLAG

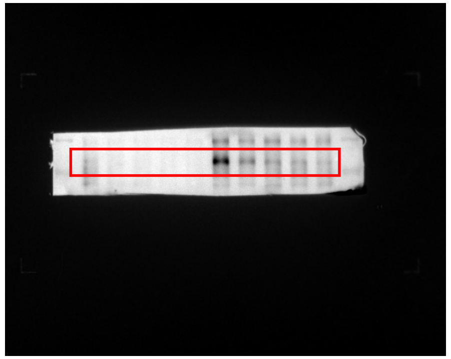

β-actin

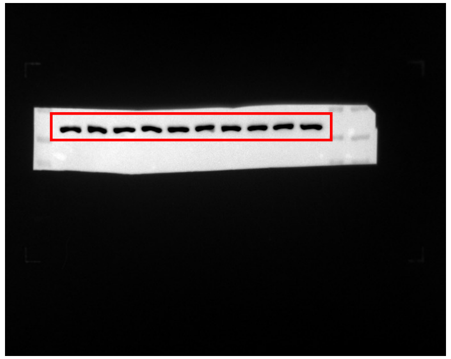

Figure 6I

FLAG

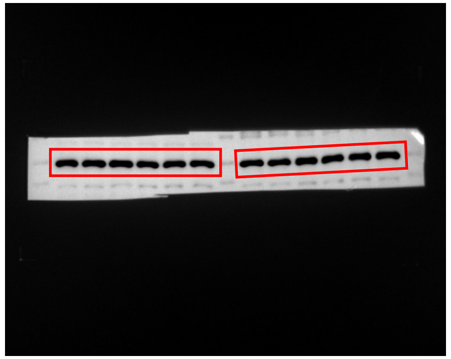

HA input

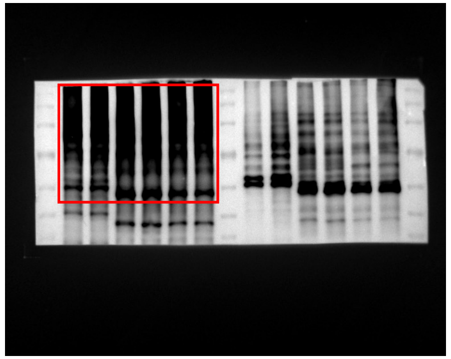

HA IP

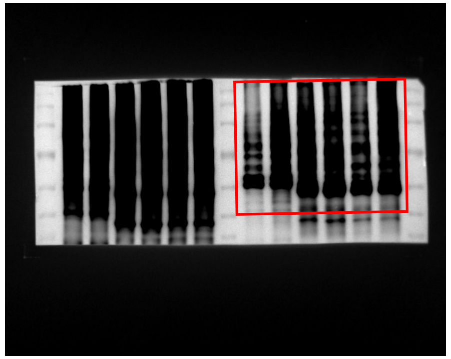

MYC

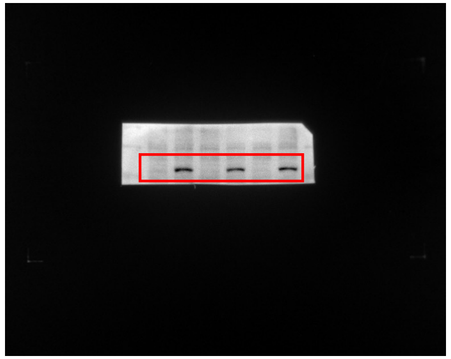

Figure 7A

MID2

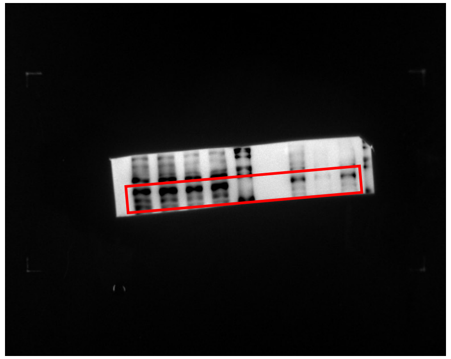

β-actin

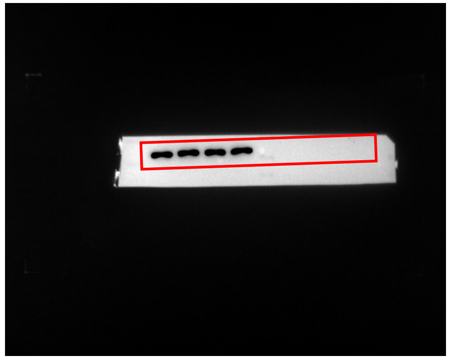

FLAG

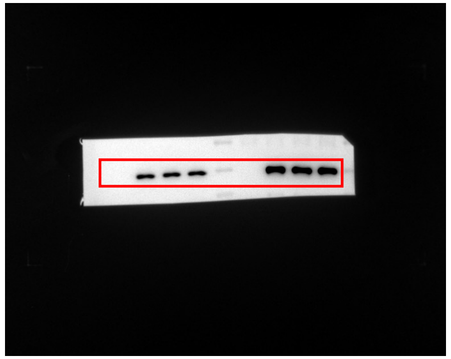

MYC

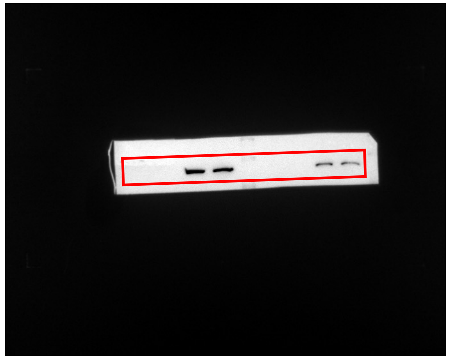

Figure 7B

MID2

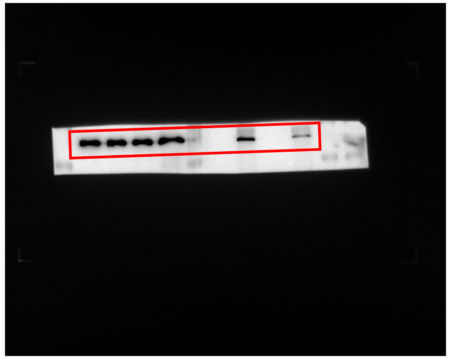

p-p65

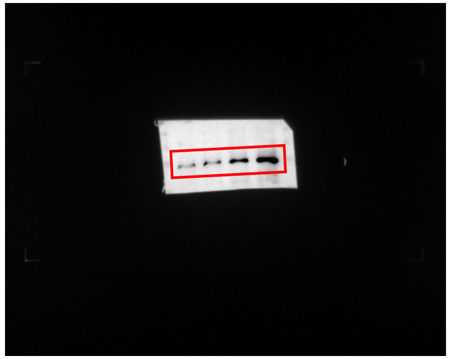

FLAG

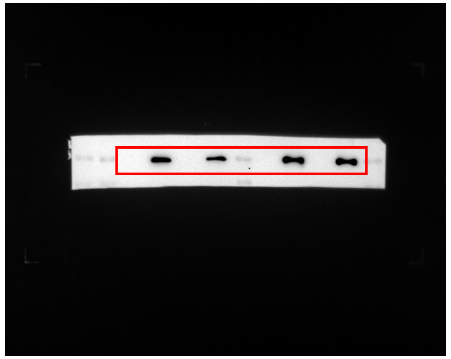

β-actin

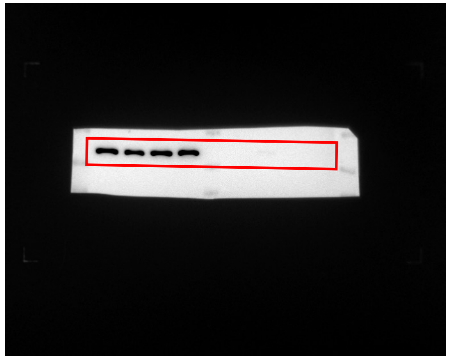

Figure 7C

pan-Ser

HA

MYC

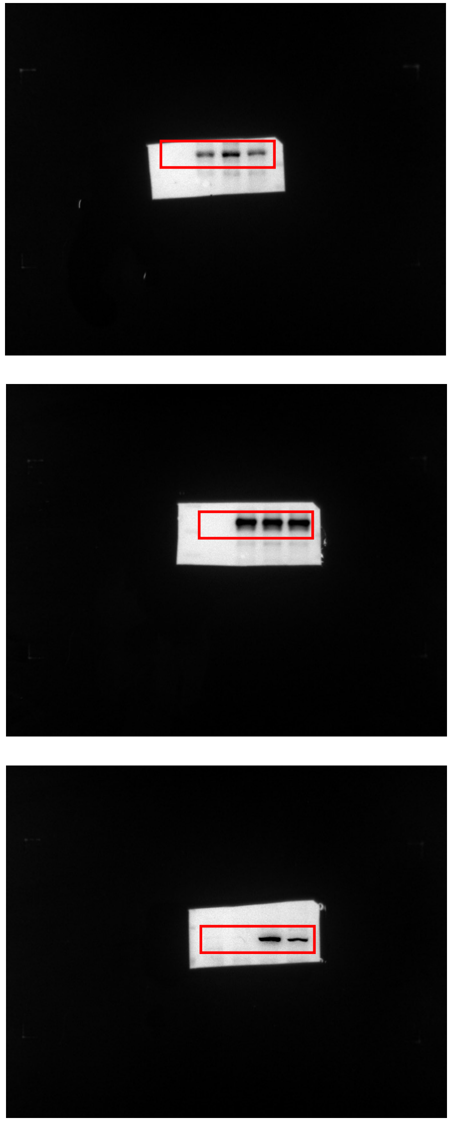

Figure 7D

pan-Ser

HA

MYC

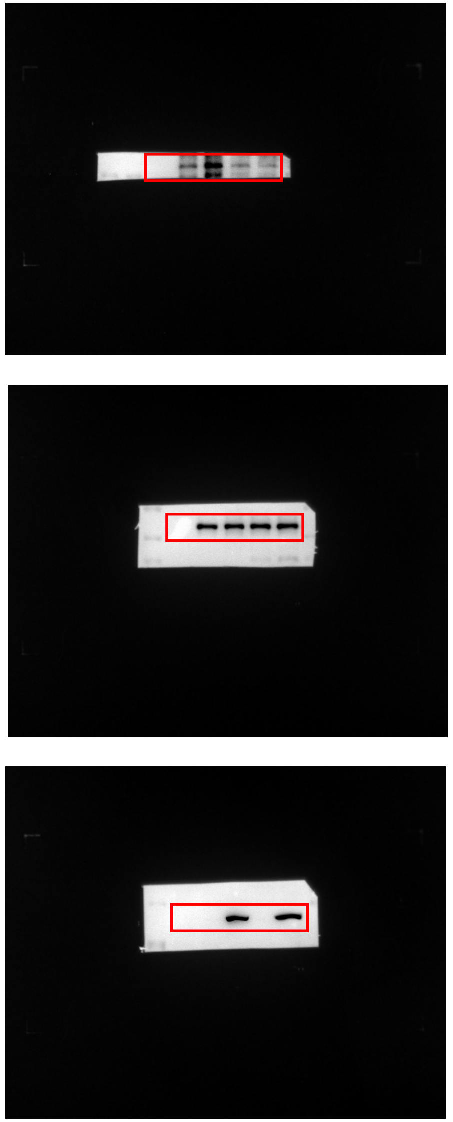

Figure 7E

p-S213

FLAG

MYC

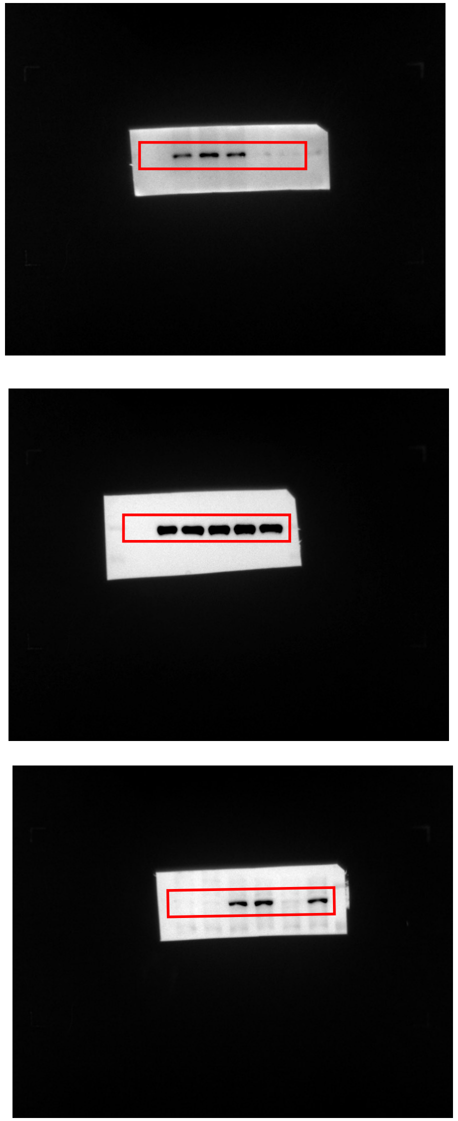

Figure 7F

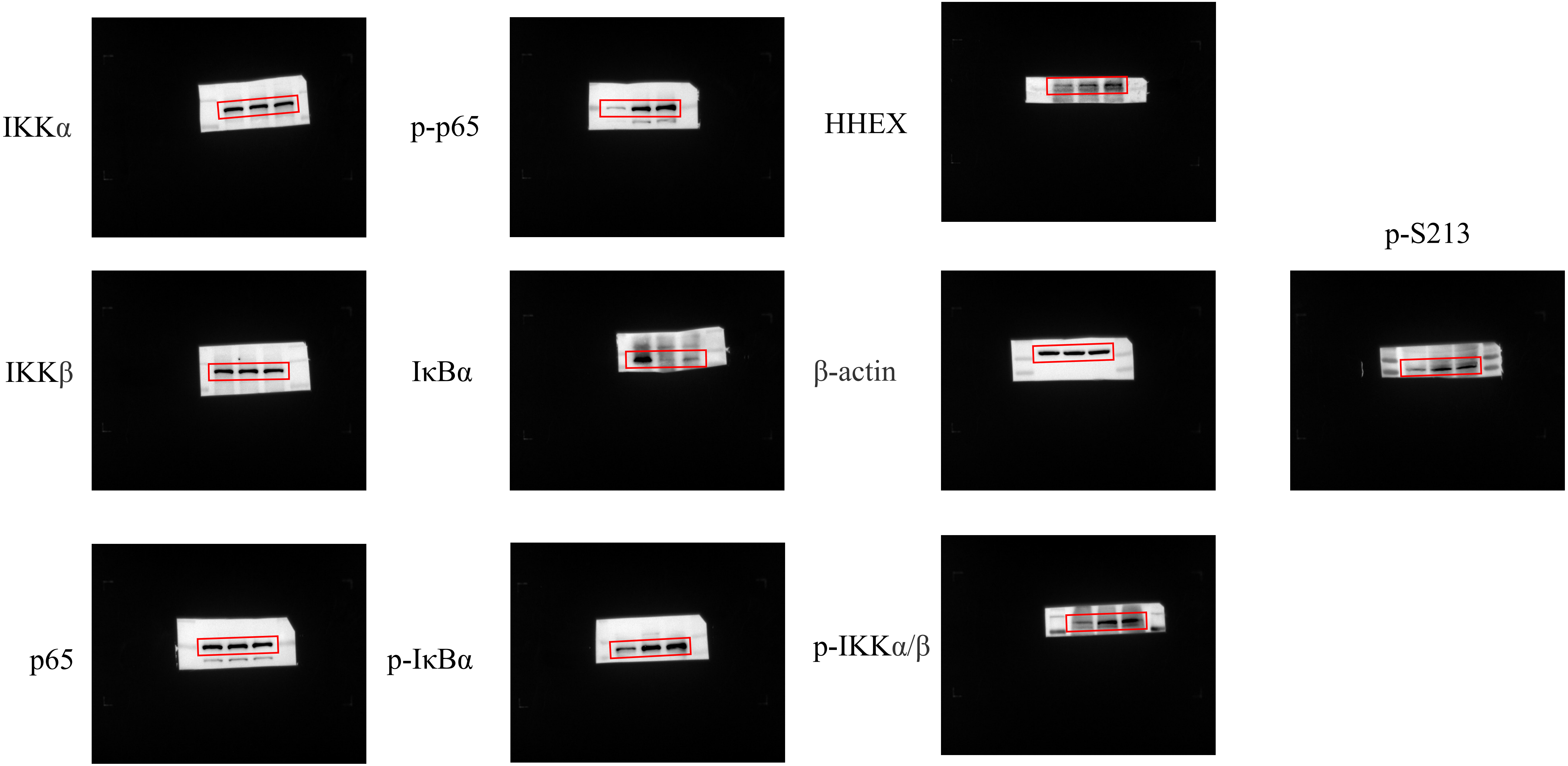

Figure 7G

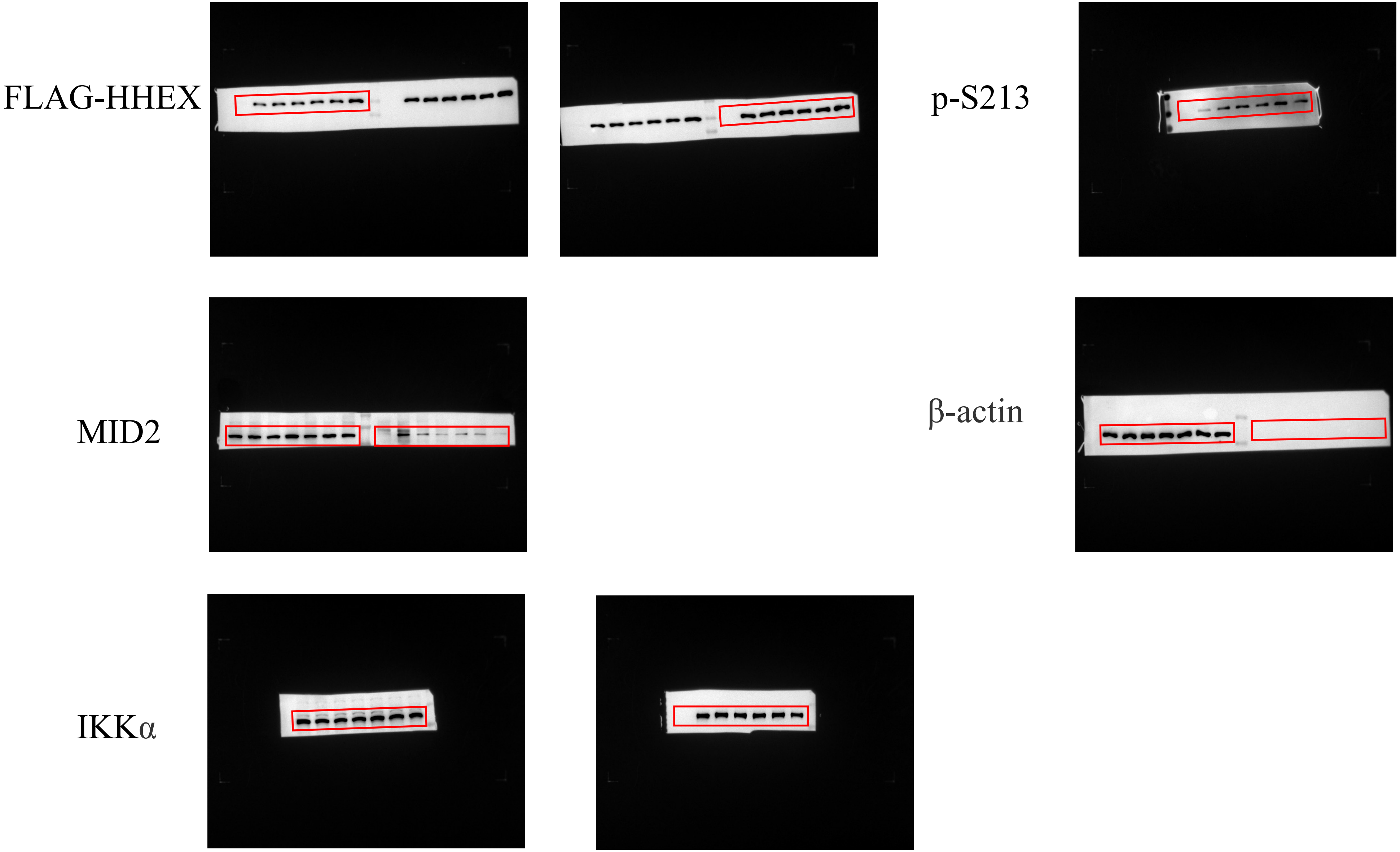

Figure 8B

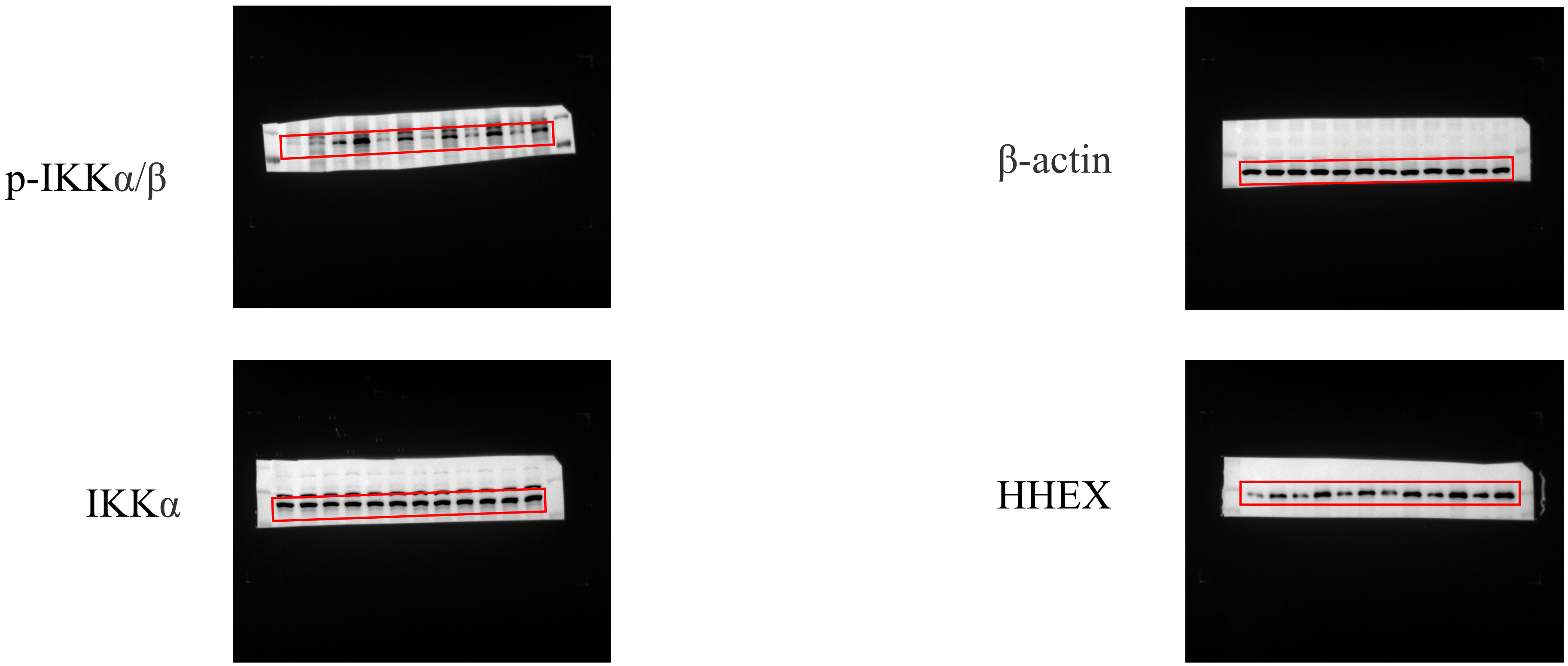

Figure 8C

HHEX

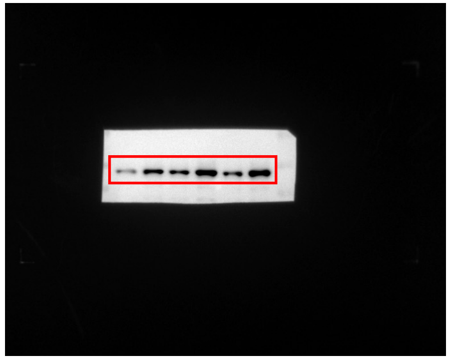

β-actin

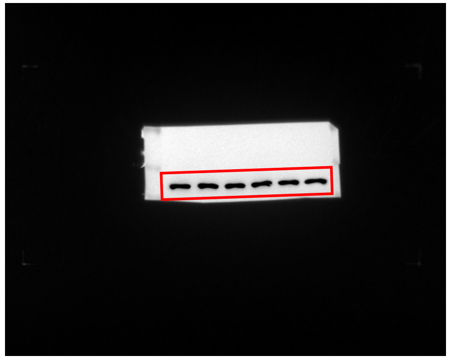

p52

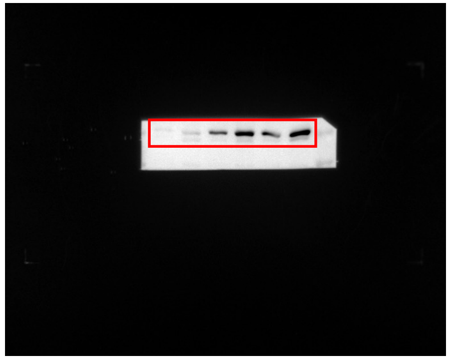

p100

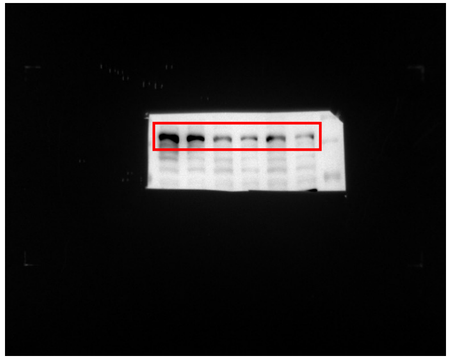

Figure 8D

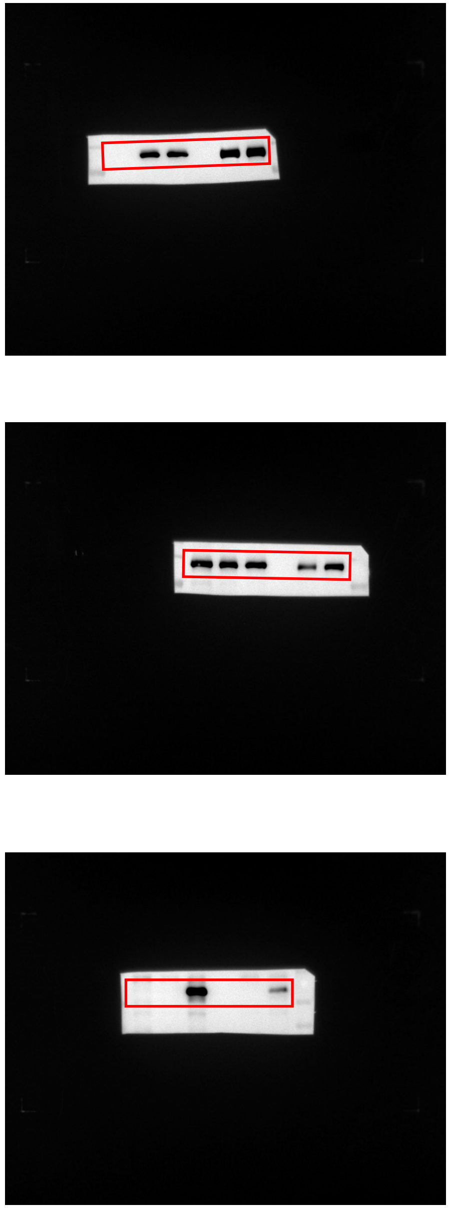

FLAG

HA-IKKα

HA-HHEX

Figure 8E

FLAG-HHEX

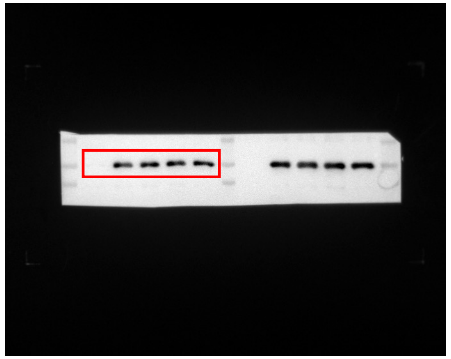

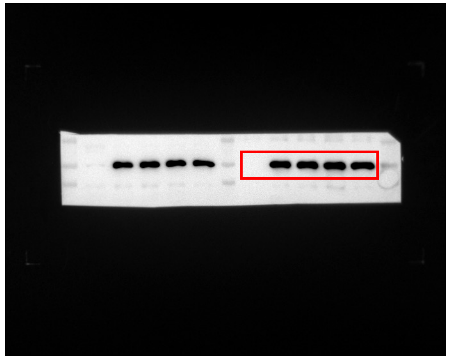

p-S213

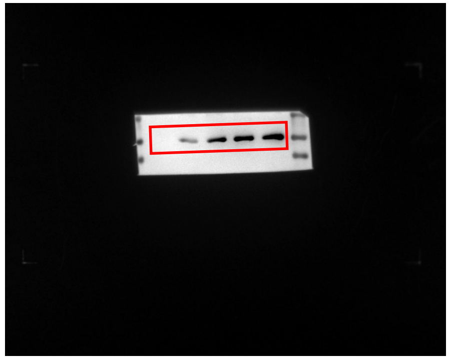

MID2

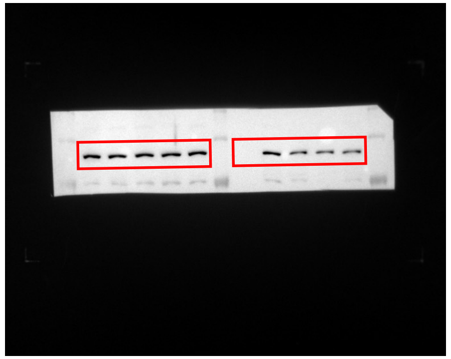

β-actin

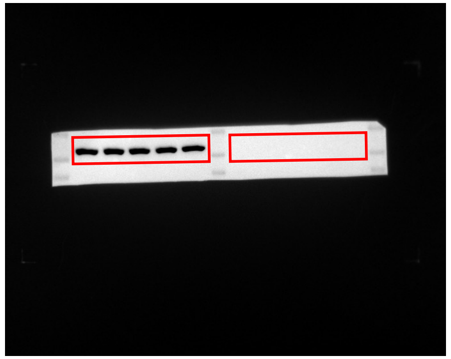

IKKα

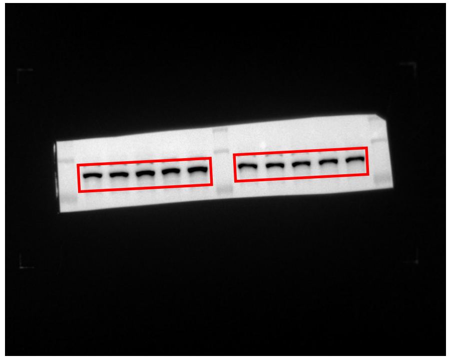

Supplementary Figure 1C

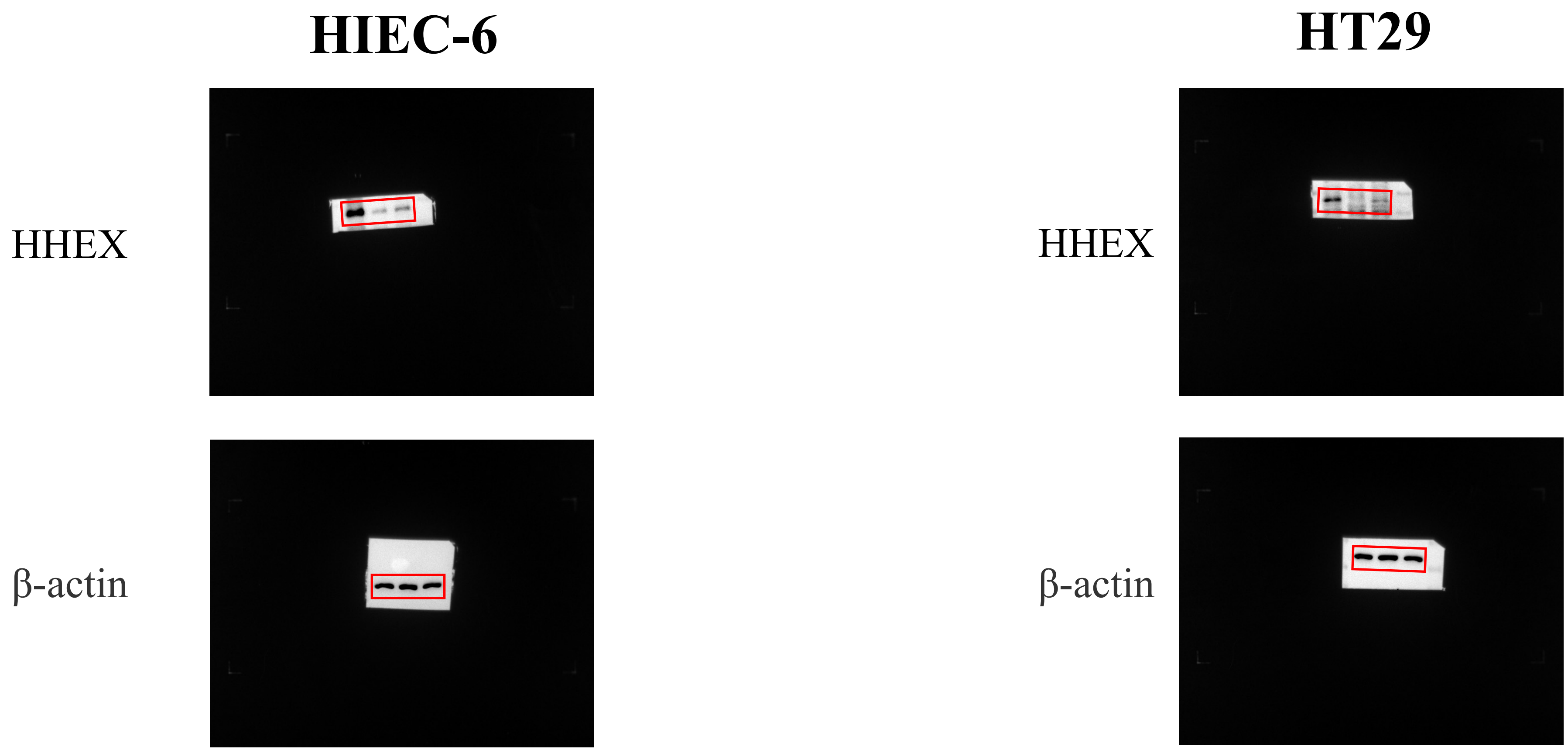

Supplementary Figure 1D

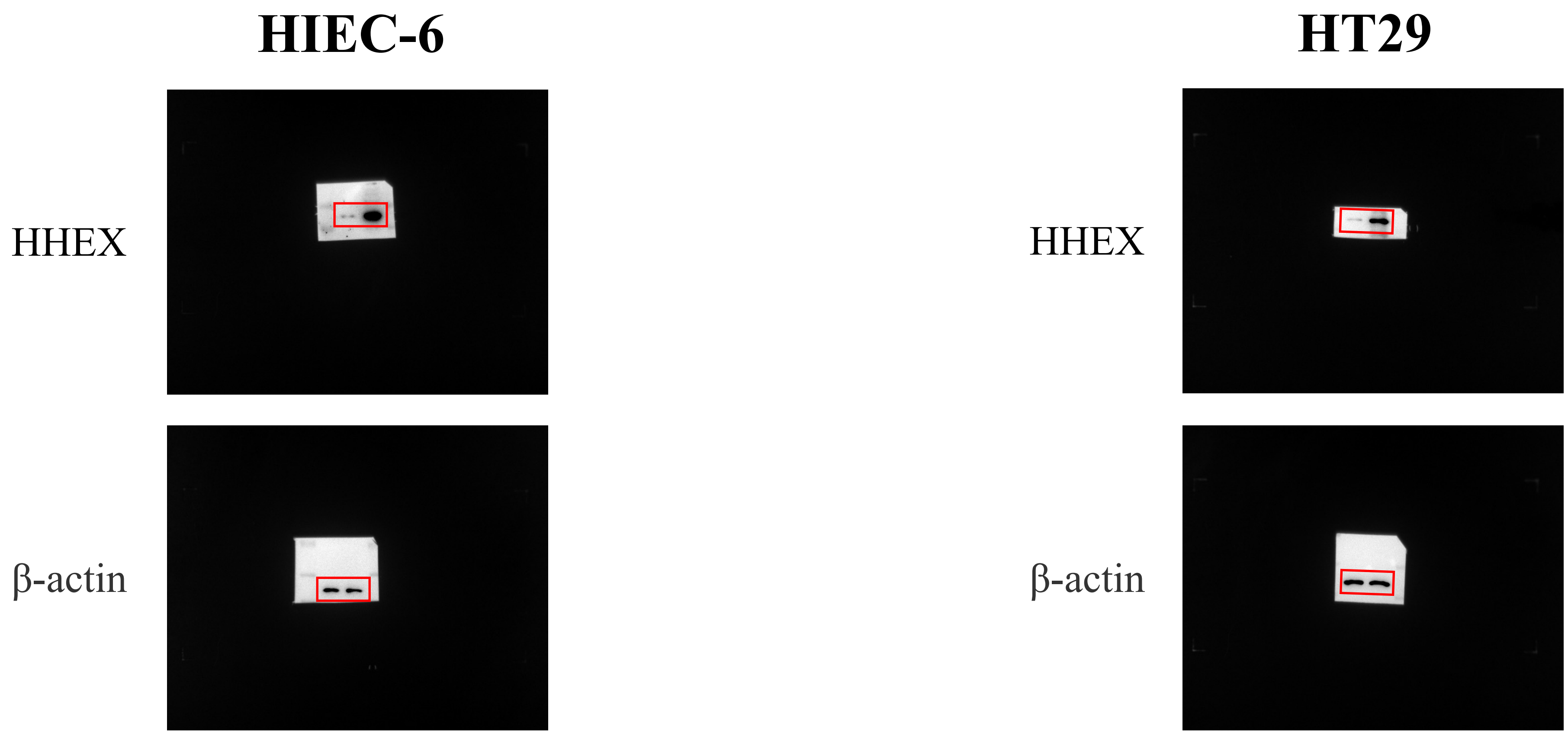

Supplementary Figure 1G HIEC-6

IKKα

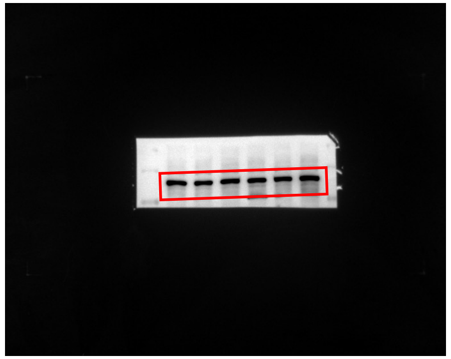

p-p65

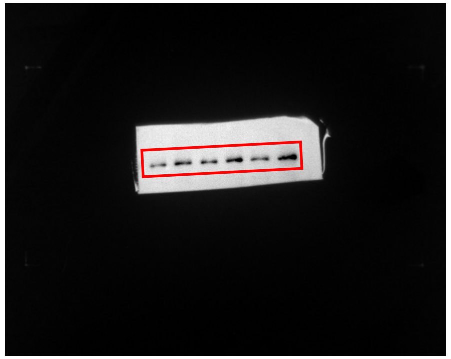

FLAG-HHEX

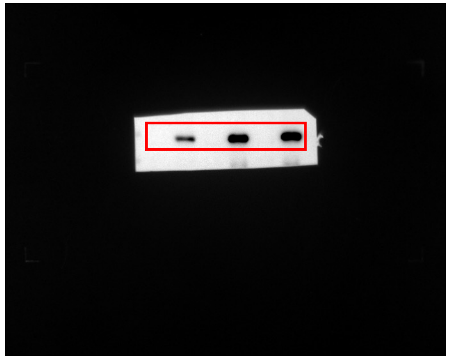

IKKβ

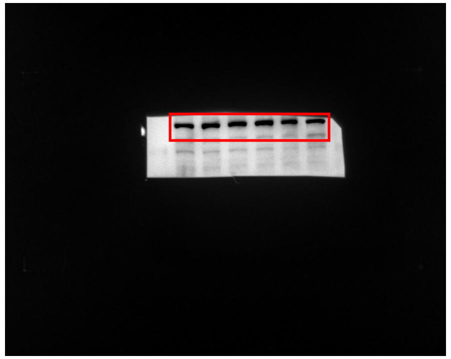

IκBα

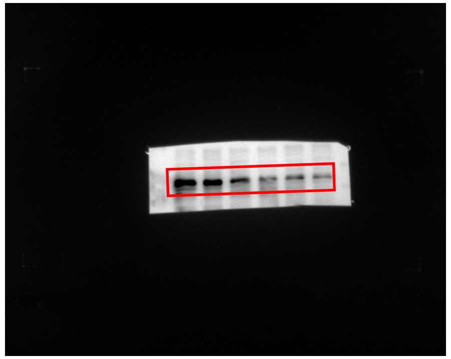

β-actin

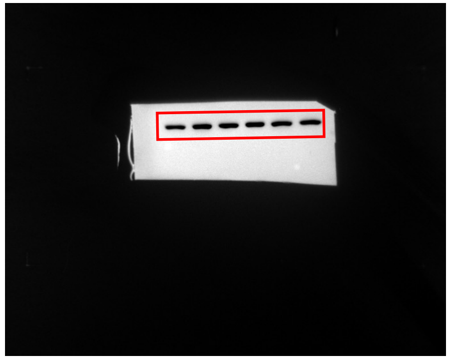

p65

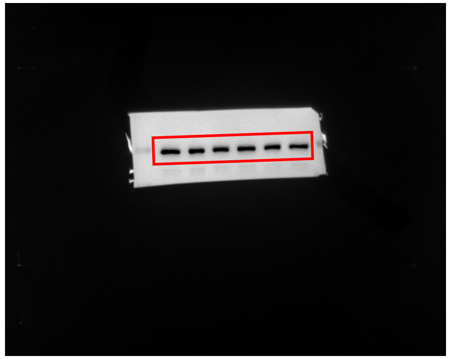

p-IκBα

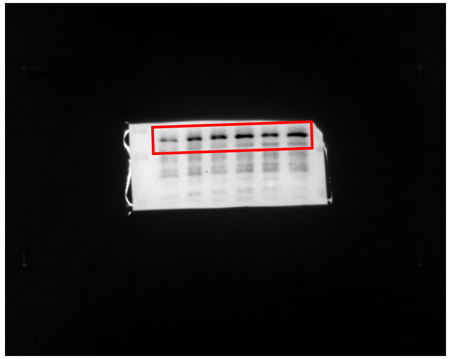

Supplementary Figure 1G HT29

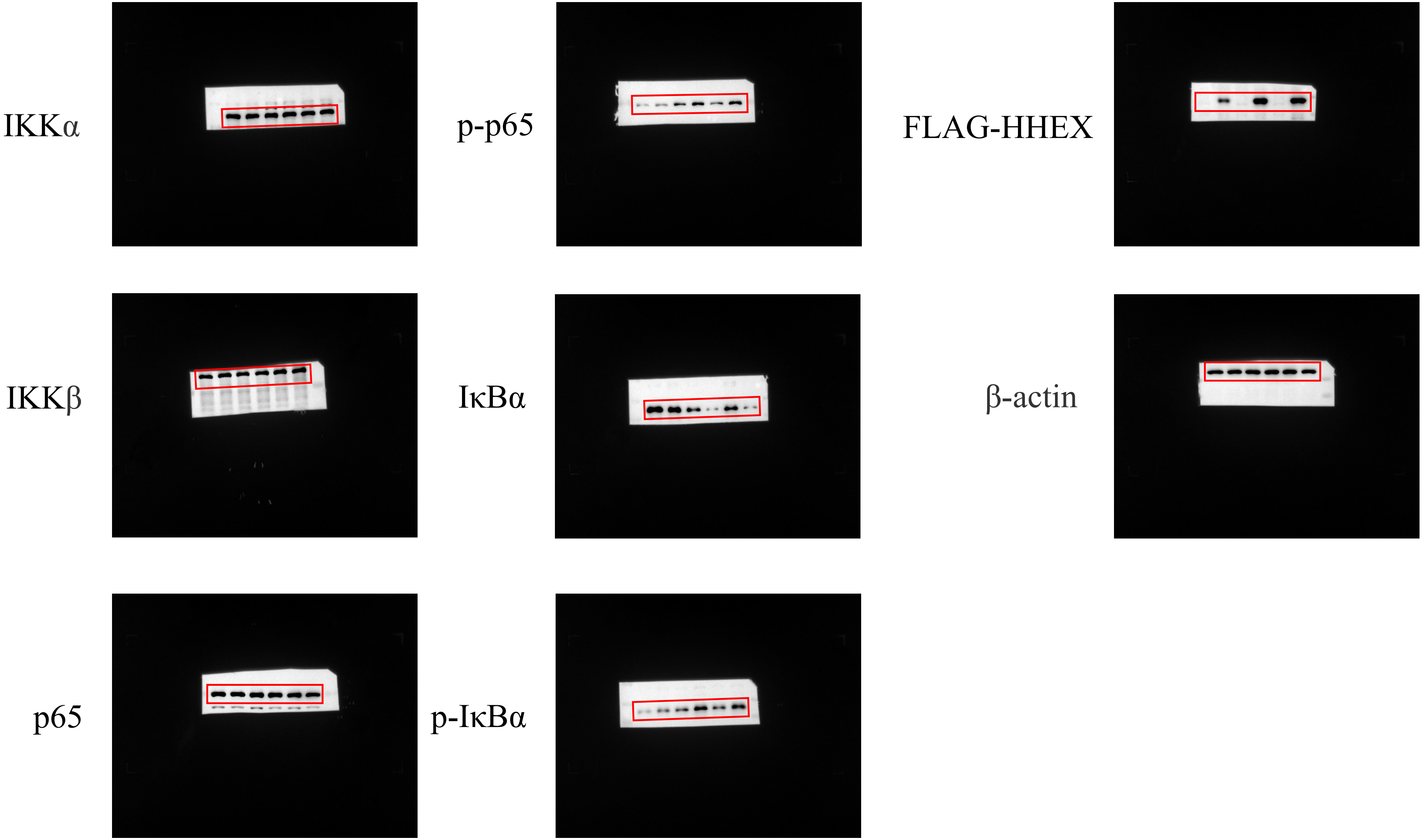

Supplementary Figure 1H HIEC-6

IKKα

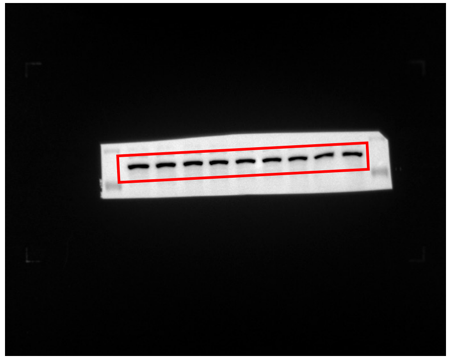

p-p65

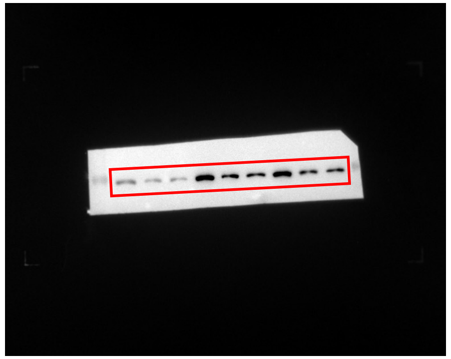

HHEX

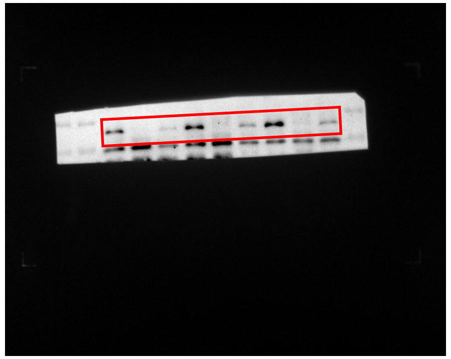

IKKβ

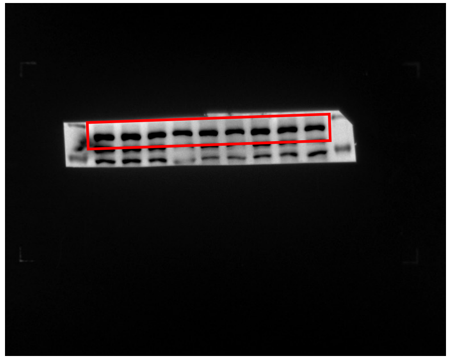

IκBα

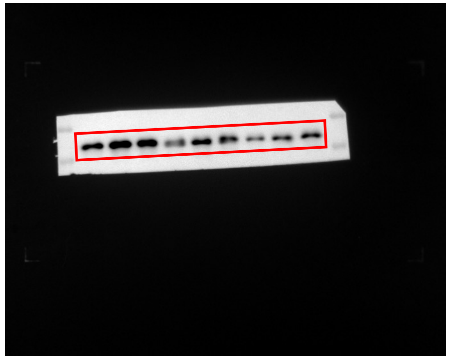

β-actin

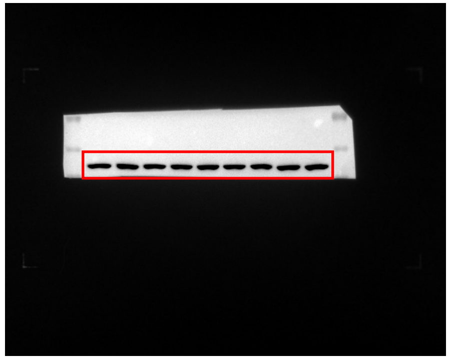

p65

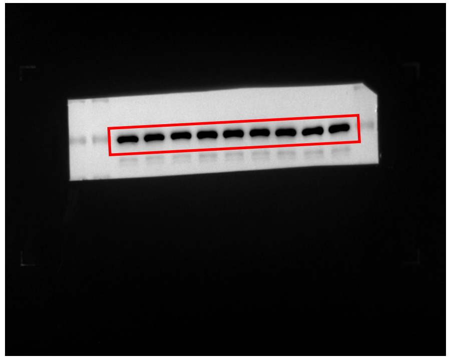

p-IκBα

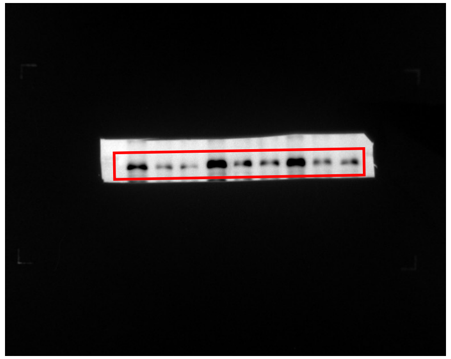

Supplementary Figure 1H HT29

IKKα

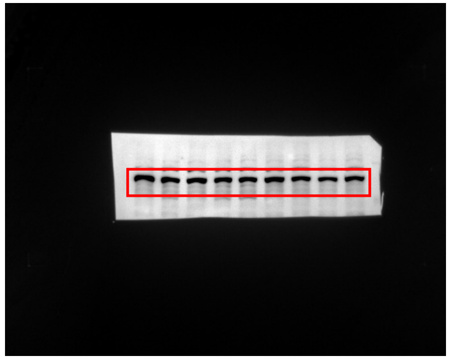

p-p65

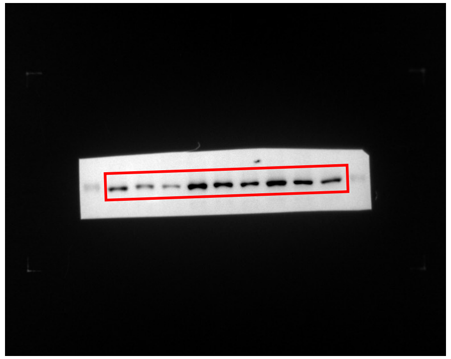

HHEX

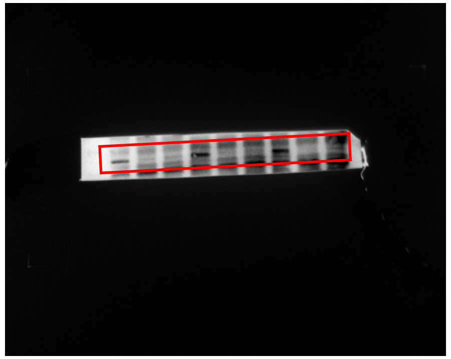

IKKβ

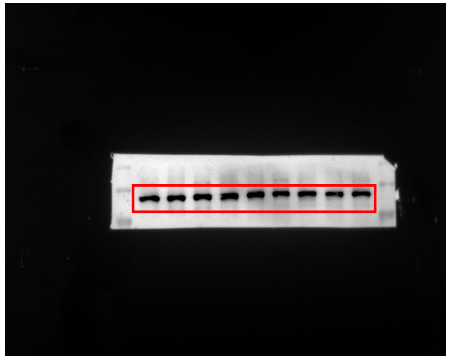

IκBα

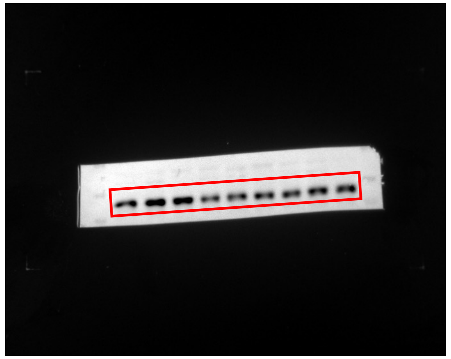

β-actin

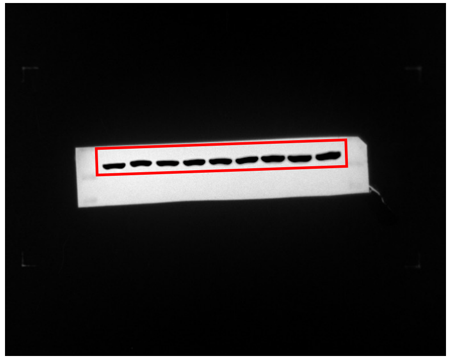

p65

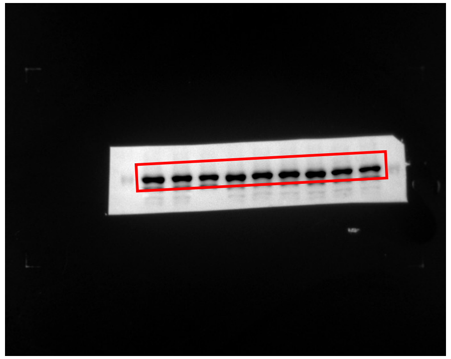

p-IκBα

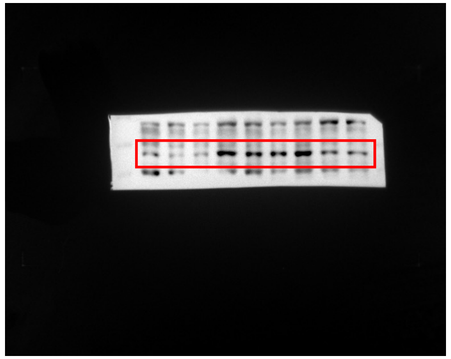

Supplementary Figure 2C

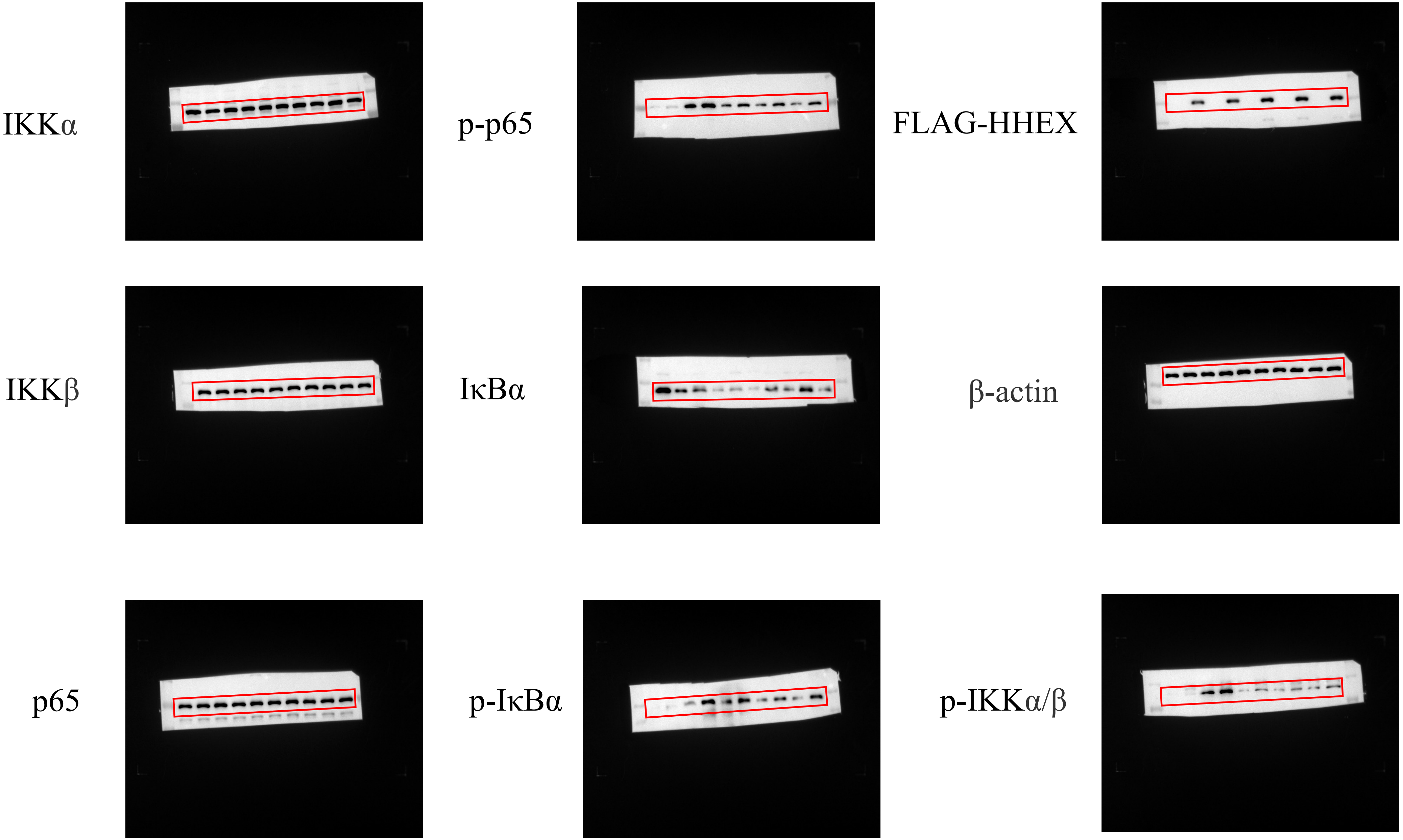

Supplementary Figure 2D

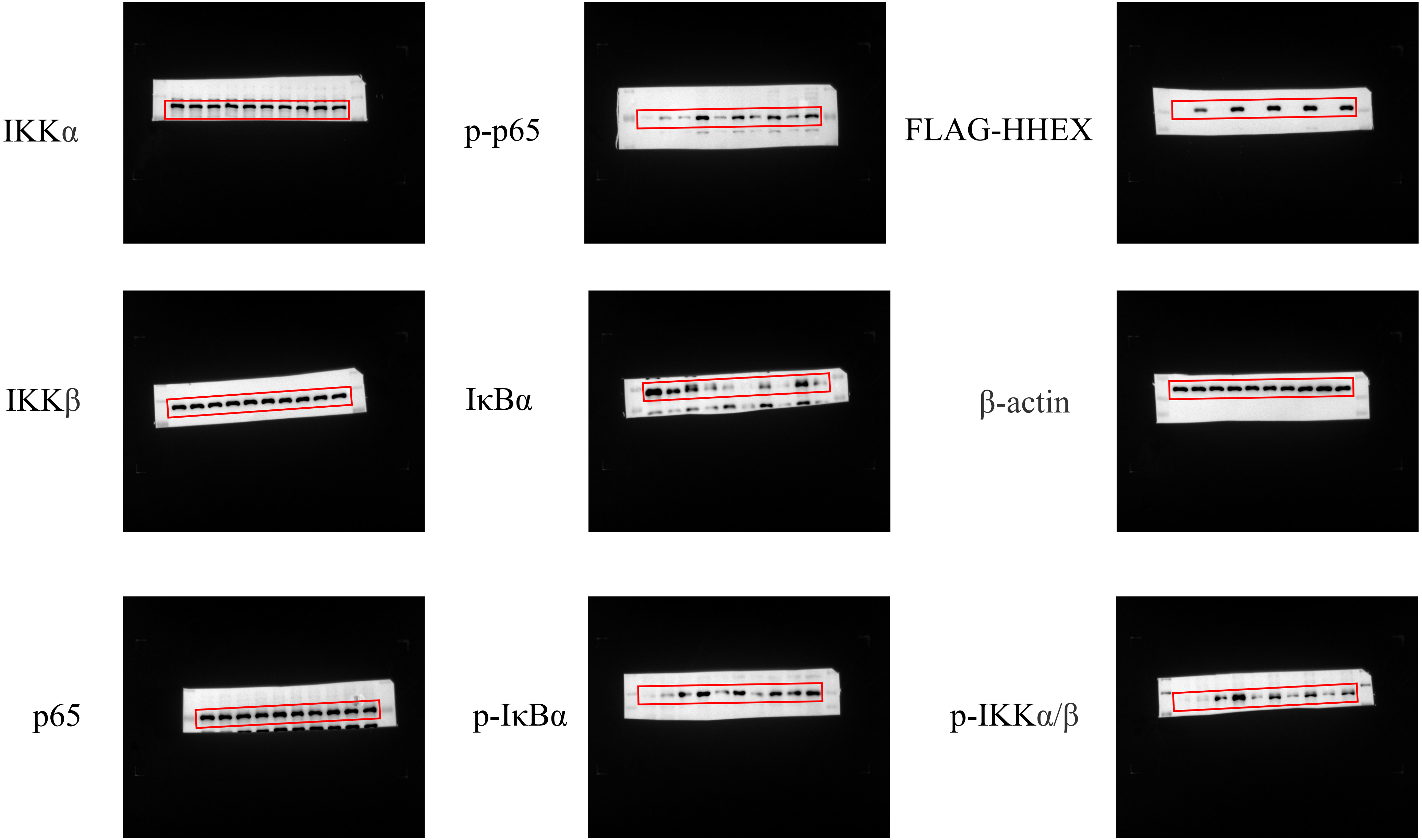

Supplementary Figure 2E

IKKβ

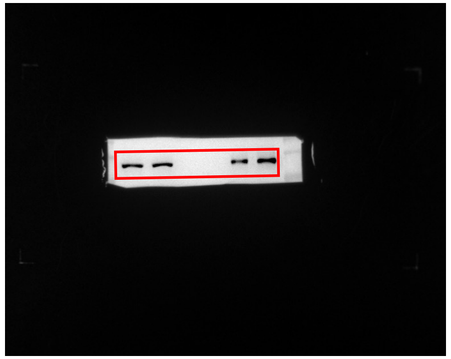

β-actin

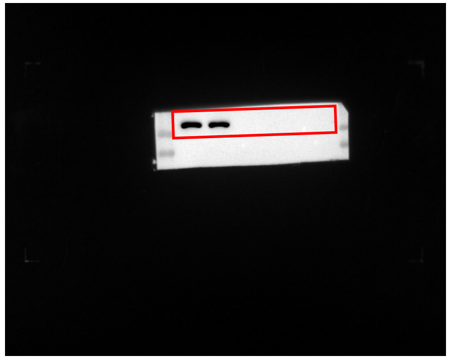

IKKα

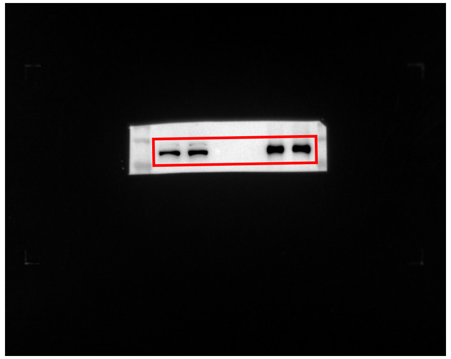

FLAG

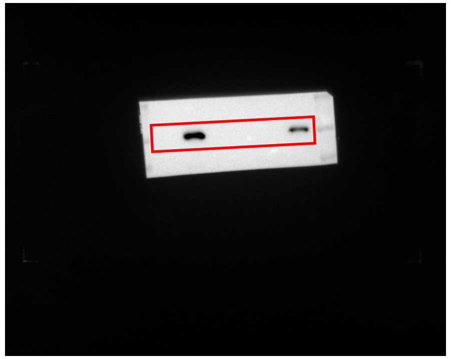

Supplementary Figure 2F

IKKβ

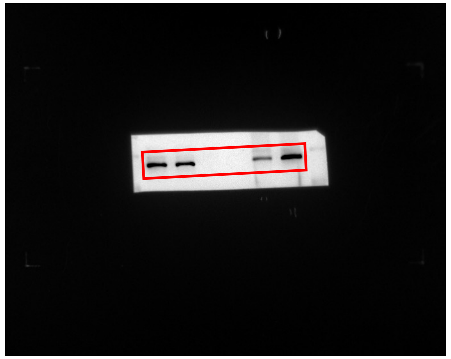

β-actin

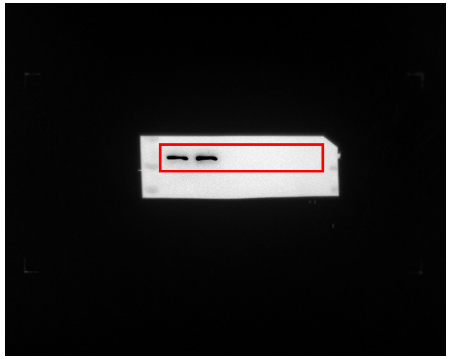

IKKα

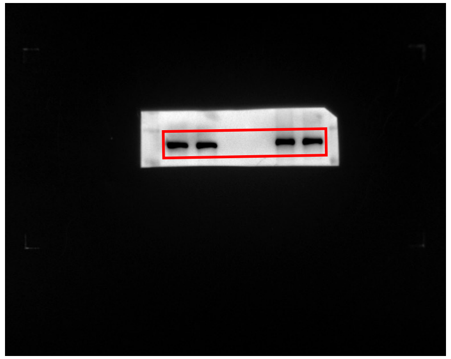

FLAG

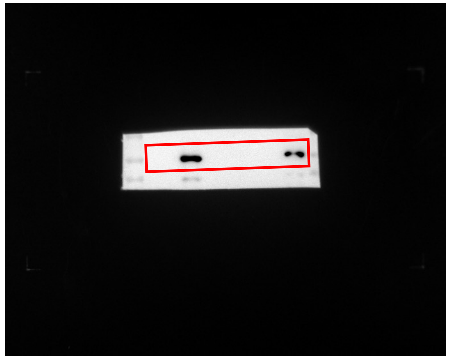

Supplementary Figure 3A

LEFT RIGHT

HA HA

FLAG FLAG

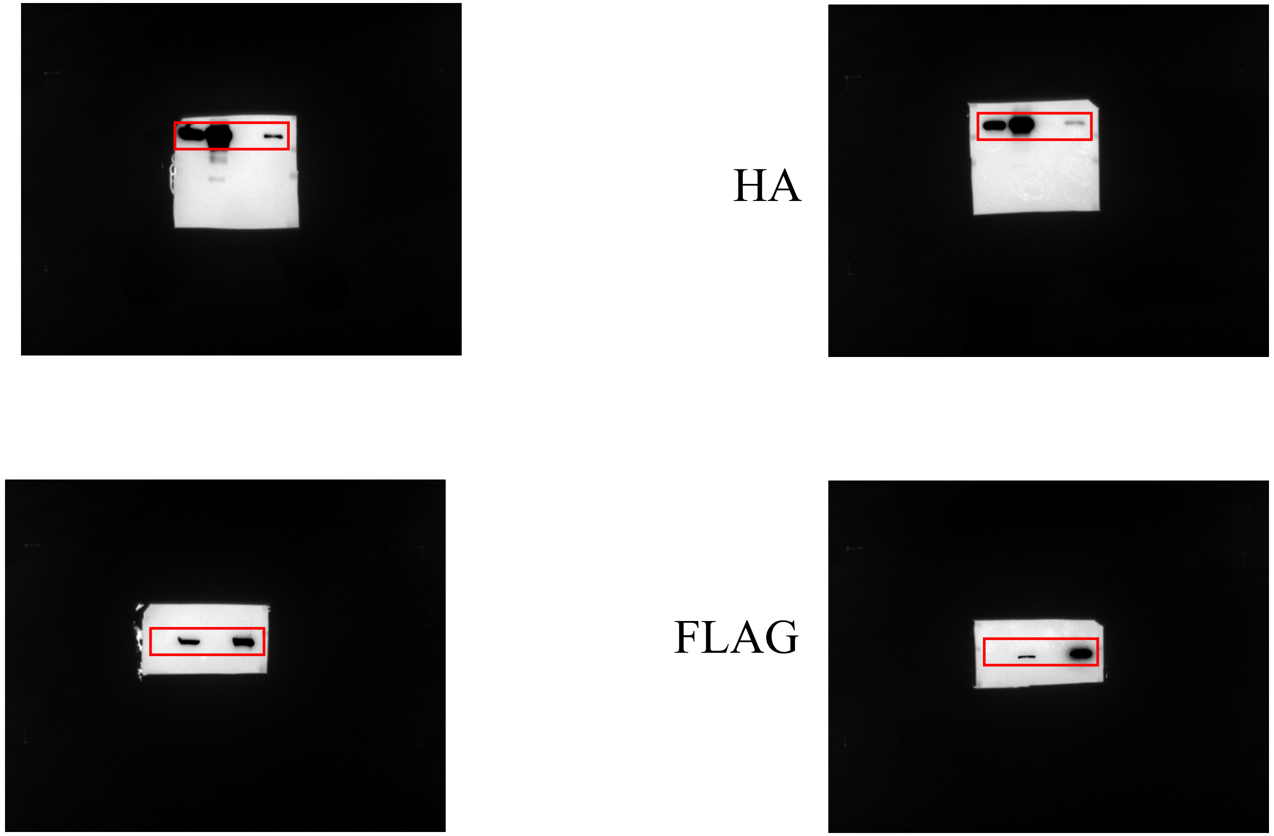

Supplementary Figure 3B LEFT

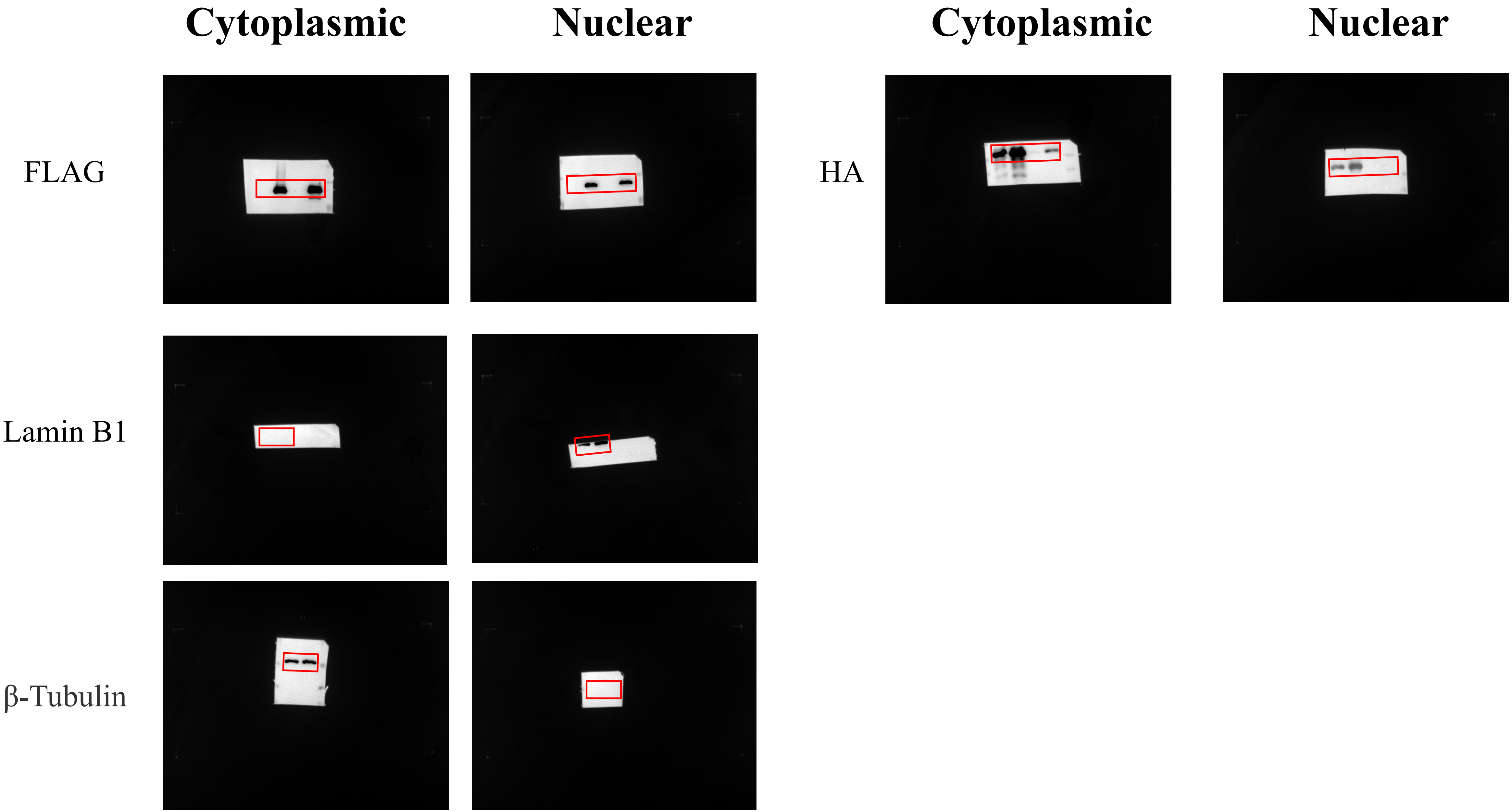

Supplementary Figure 3B RIGHT

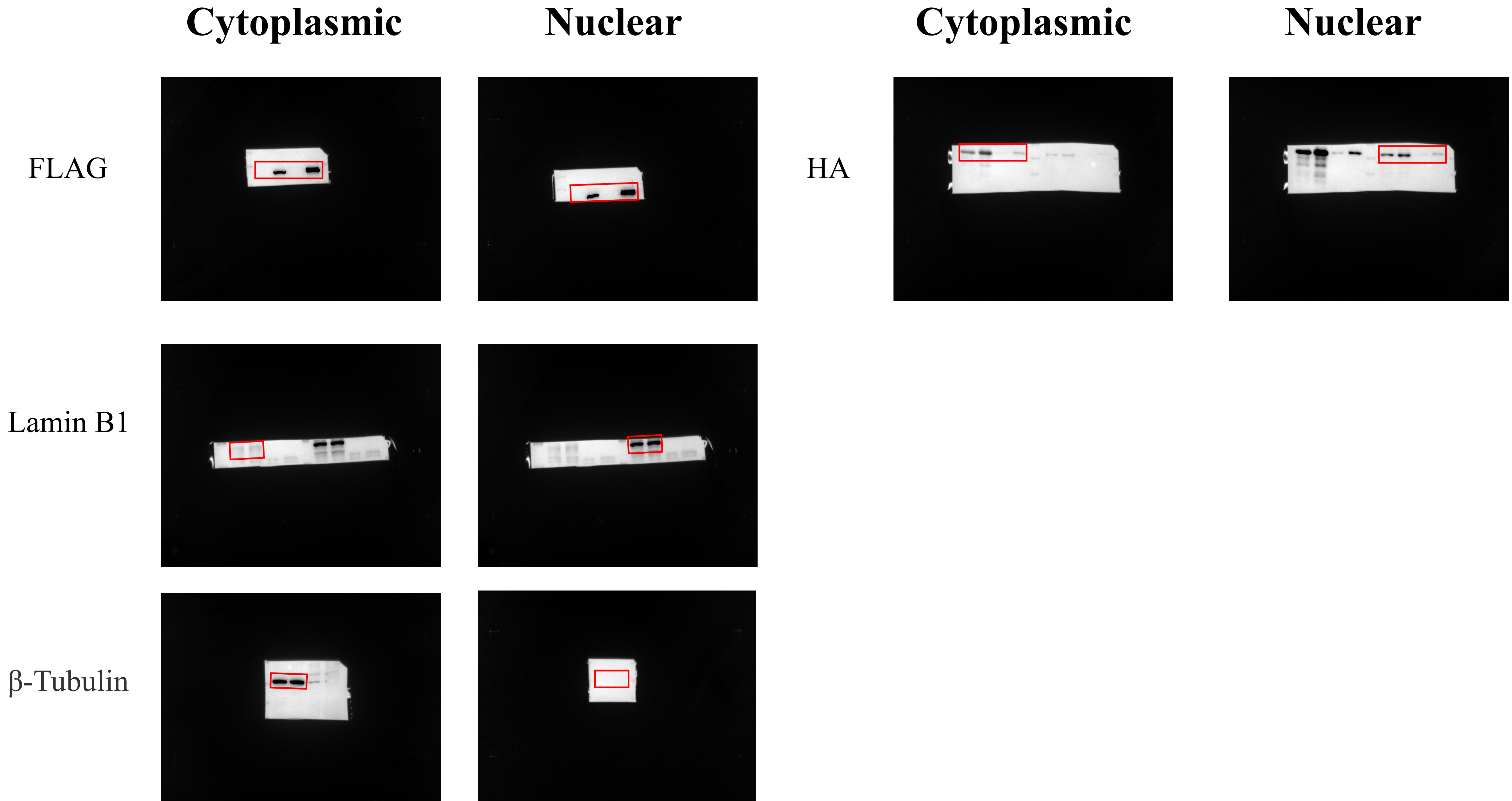

Supplementary Figure 3C LEFT

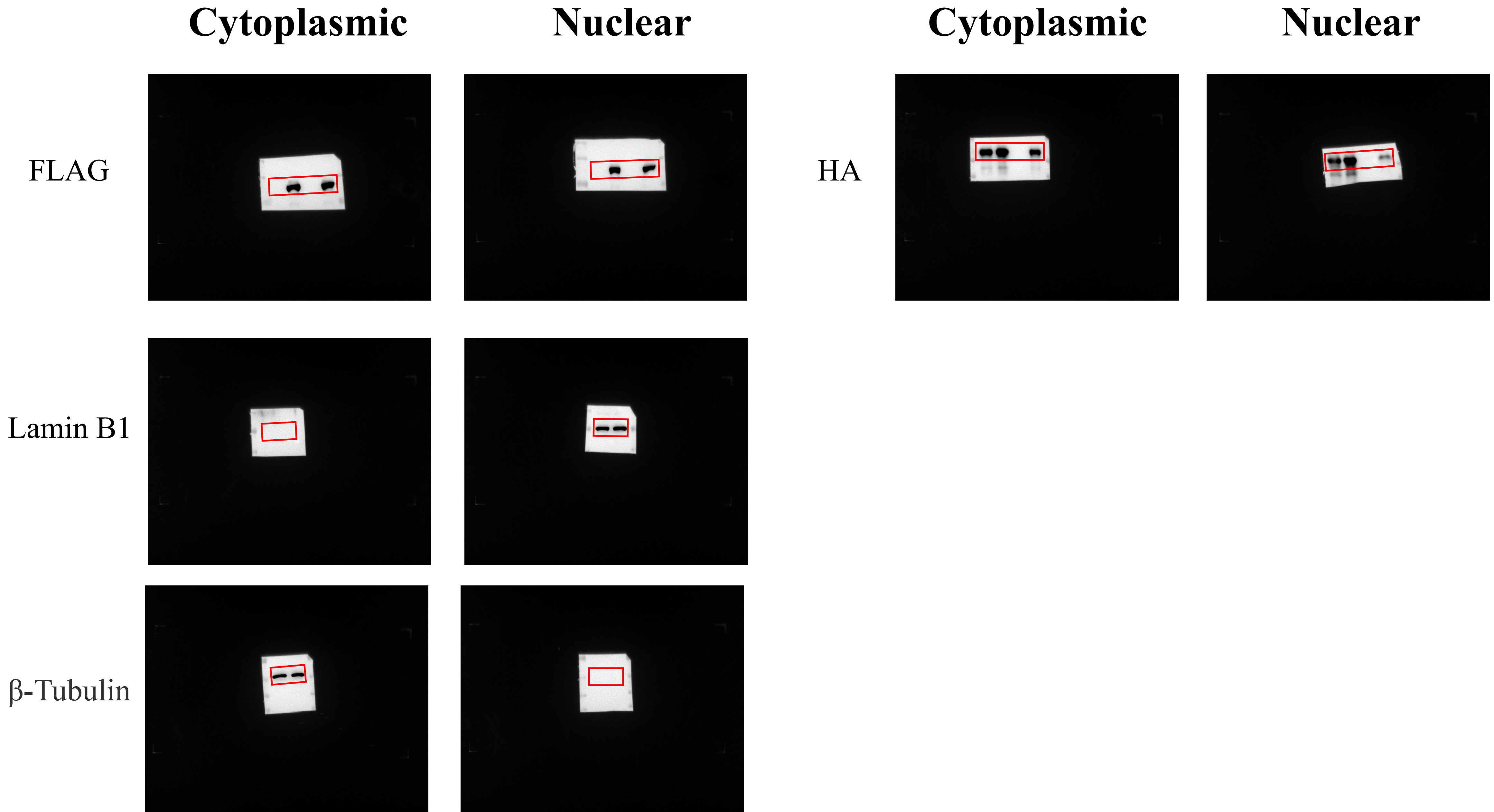

Supplementary Figure 3C RIGHT

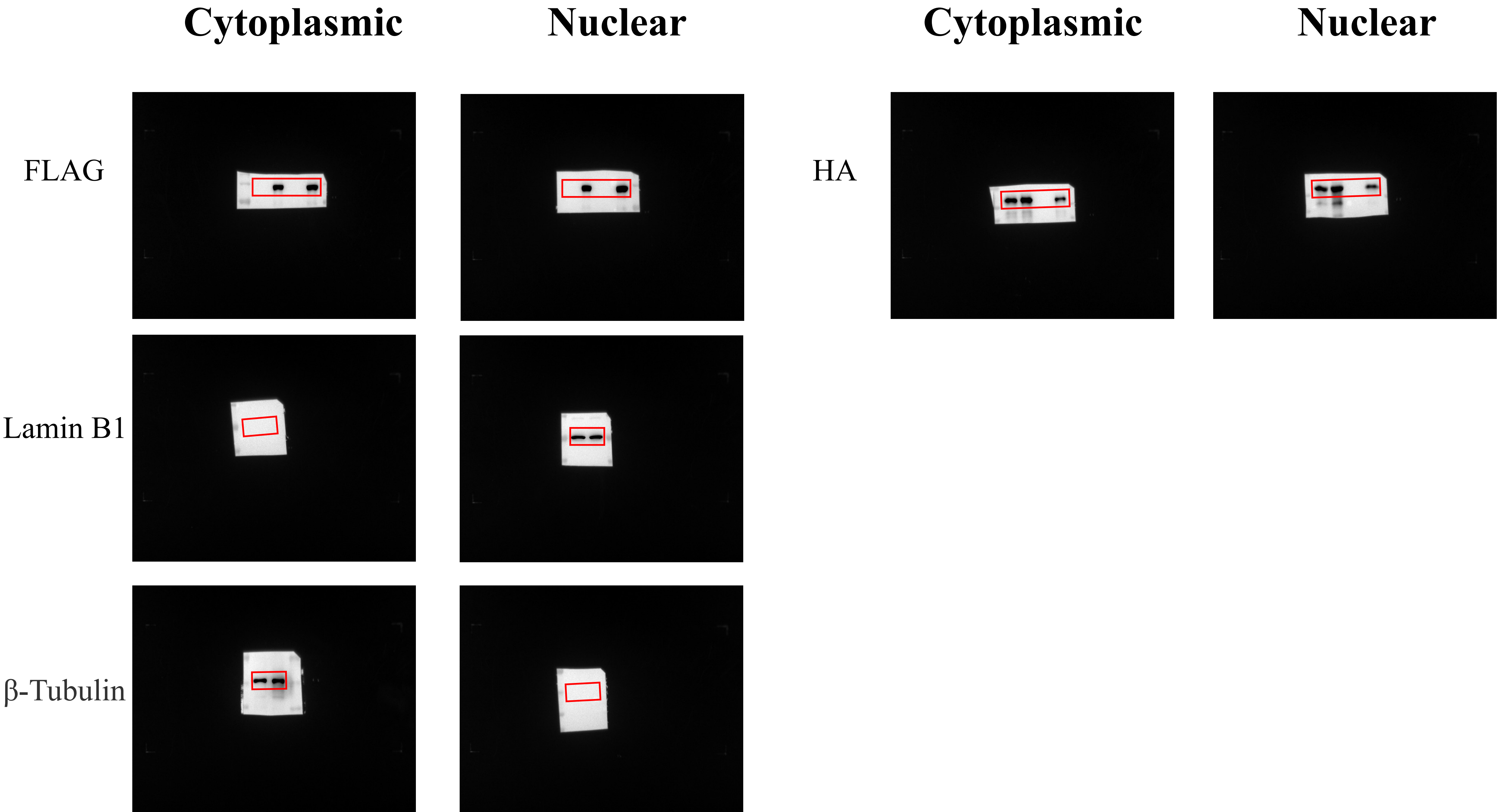

Supplementary Figure 3D

IKKβ

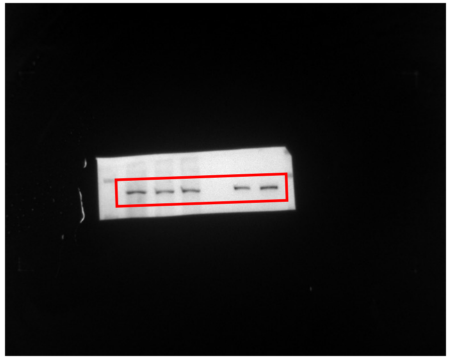

β-actin

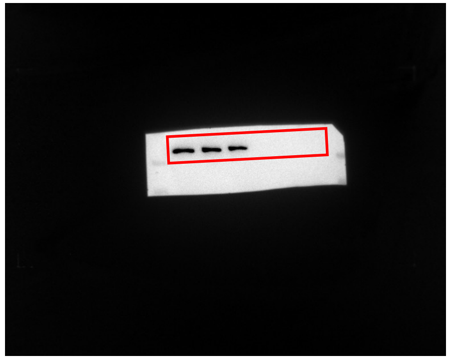

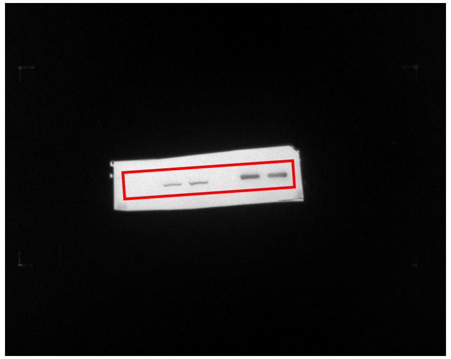

HA

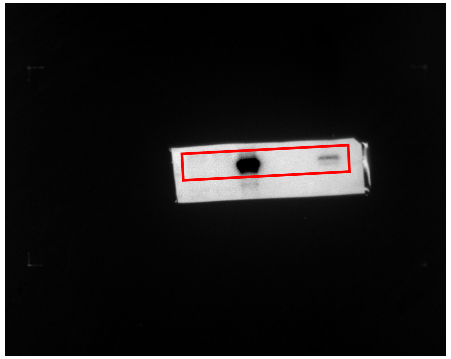

Supplementary Figure 3E

IKKα

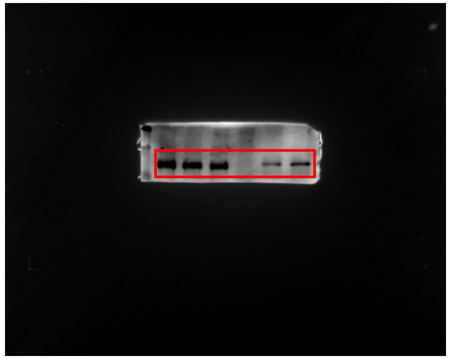

β-actin

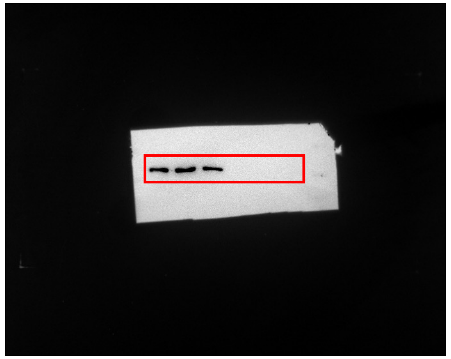

FLAG

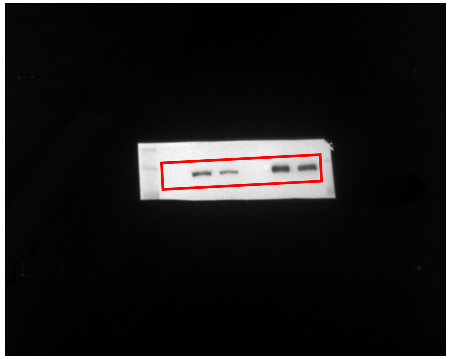

HA

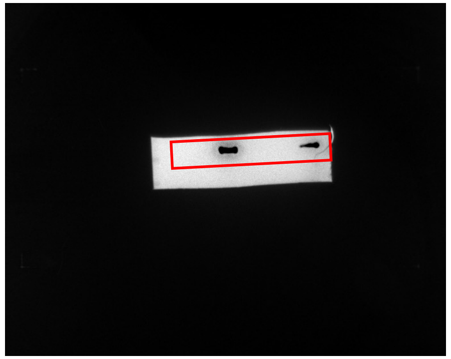

Supplementary Figure 3F

IKKβ

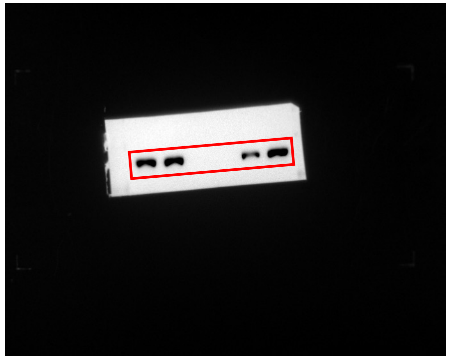

β-actin

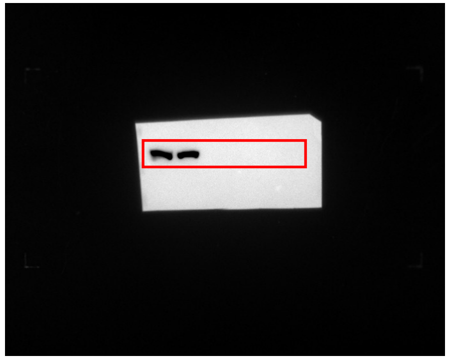

IKKα

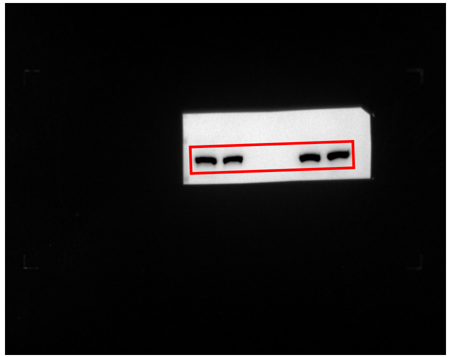

HHEX

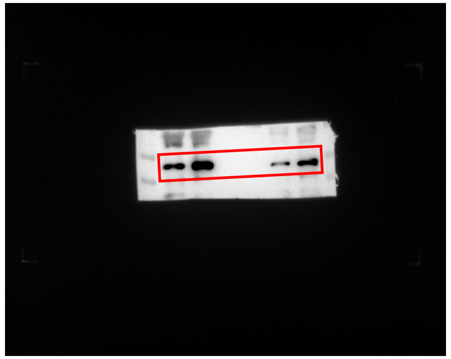

Supplementary Figure 3G

IKKβ

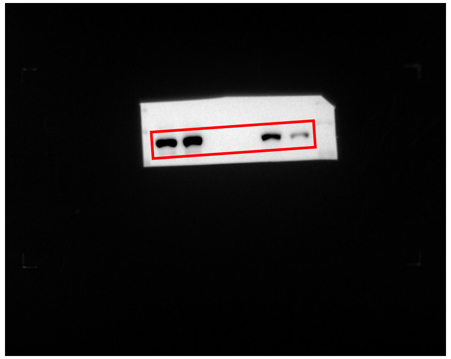

β-actin

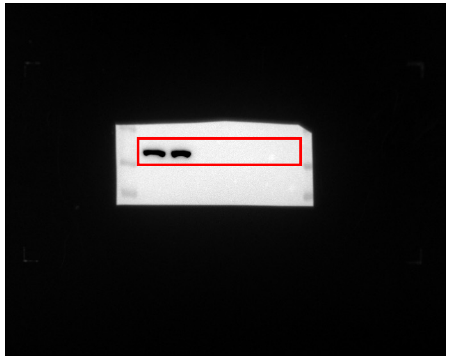

IKKα

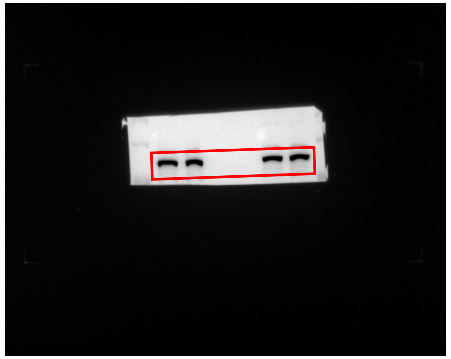

HHEX

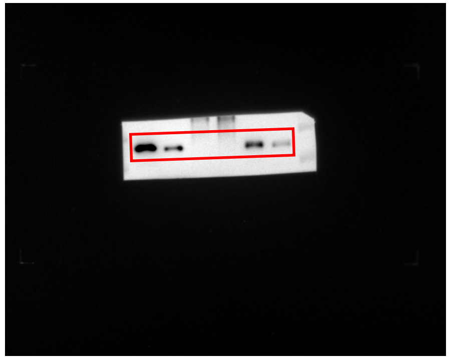

Supplementary Figure 4C

FLAG

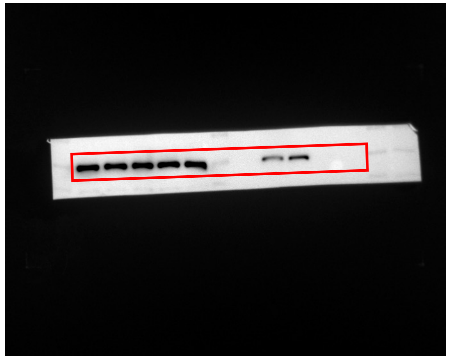

GFP

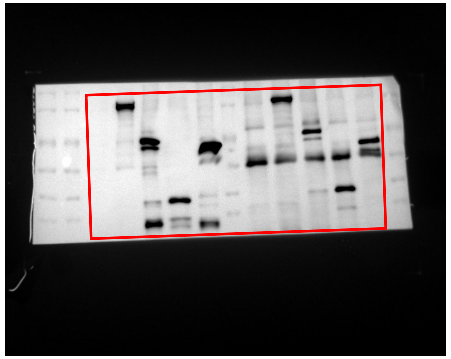

Supplementary Figure 4D

HA

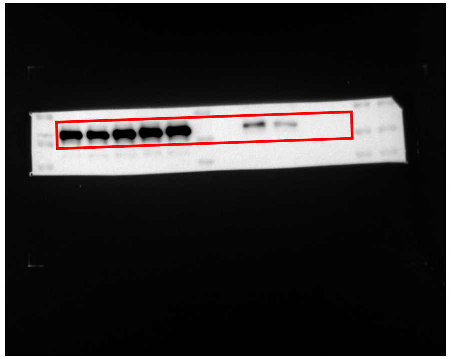

FLAG

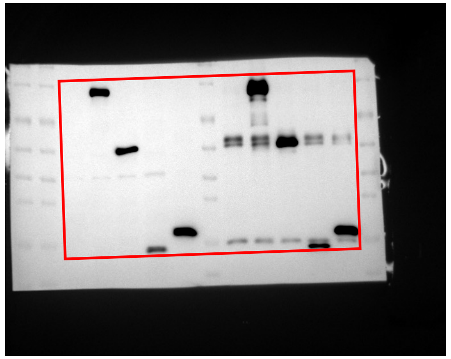

Supplementary Figure 4E

p-IKKα/β

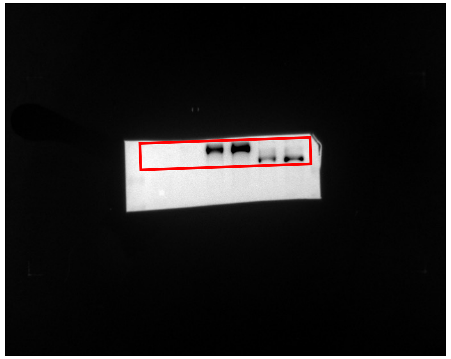

p-IκBα

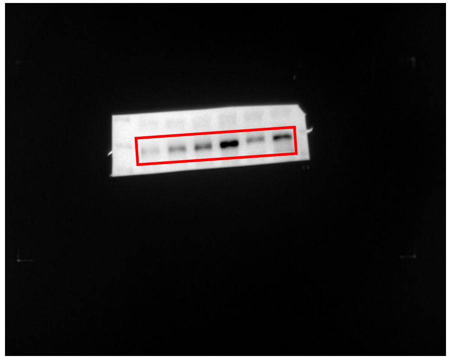

β-actin

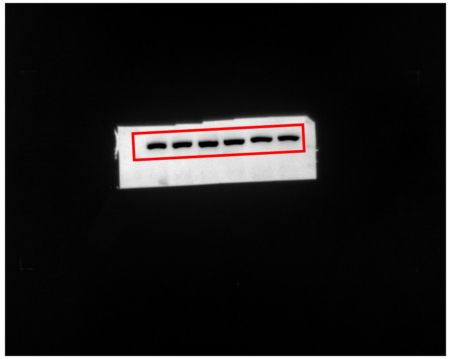

p-p65

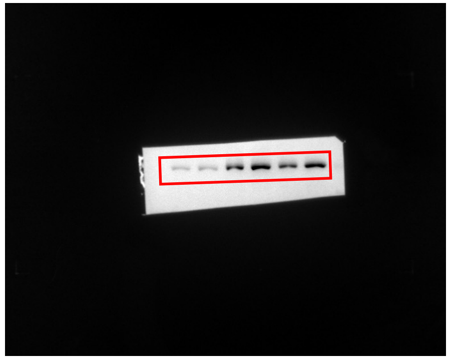

IκBα

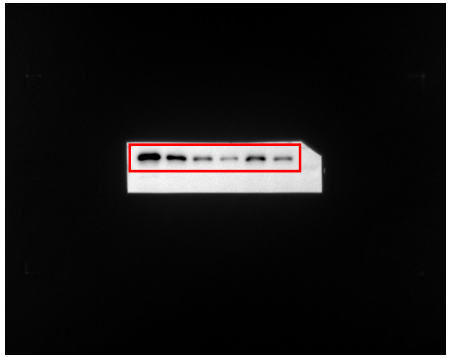

p65

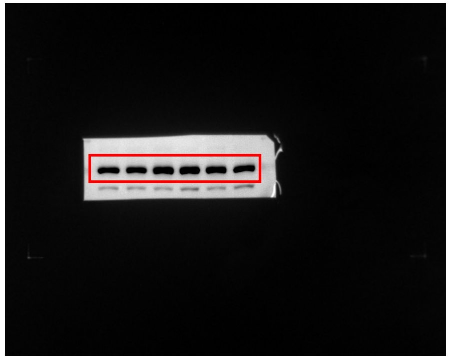

FLAG

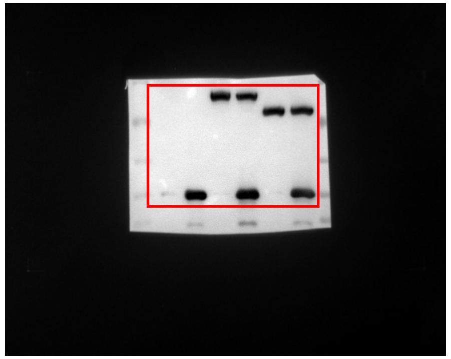

Supplementary Figure 4G

HIEC-6

IKKγ

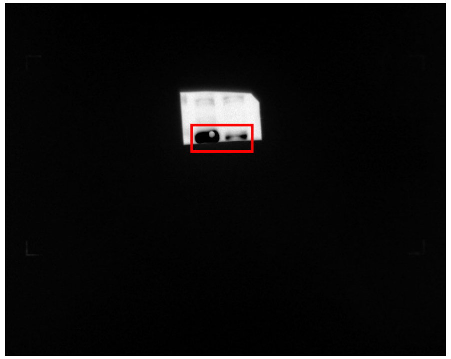

β-actin

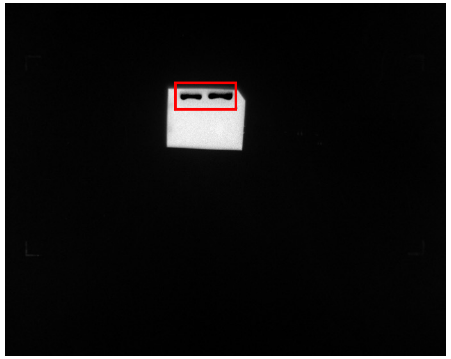

HEK293T

IKKγ

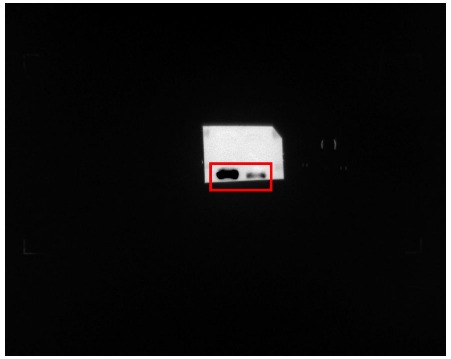

β-actin

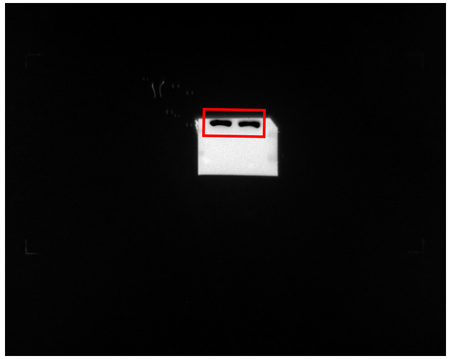

Supplementary Figure 4H

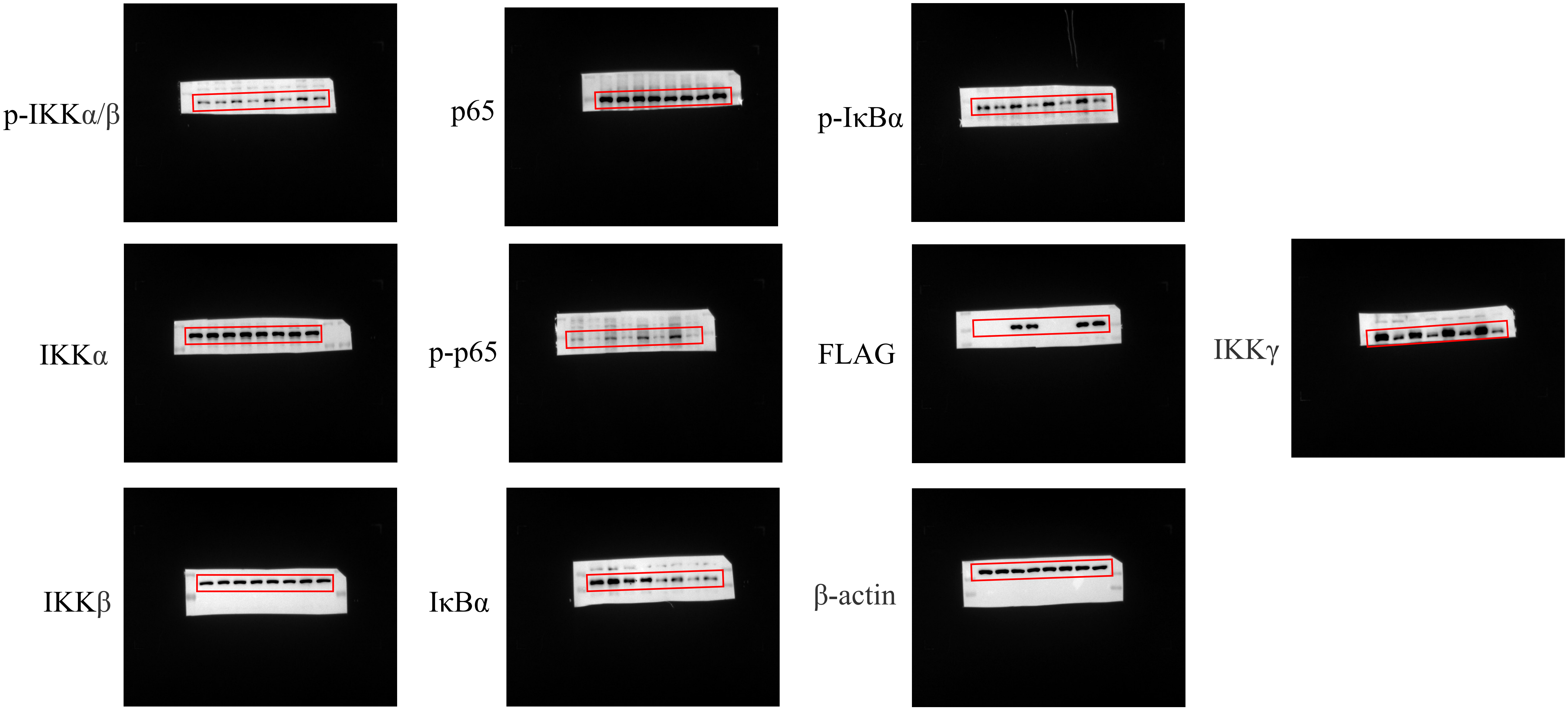

Supplementary Figure 4I

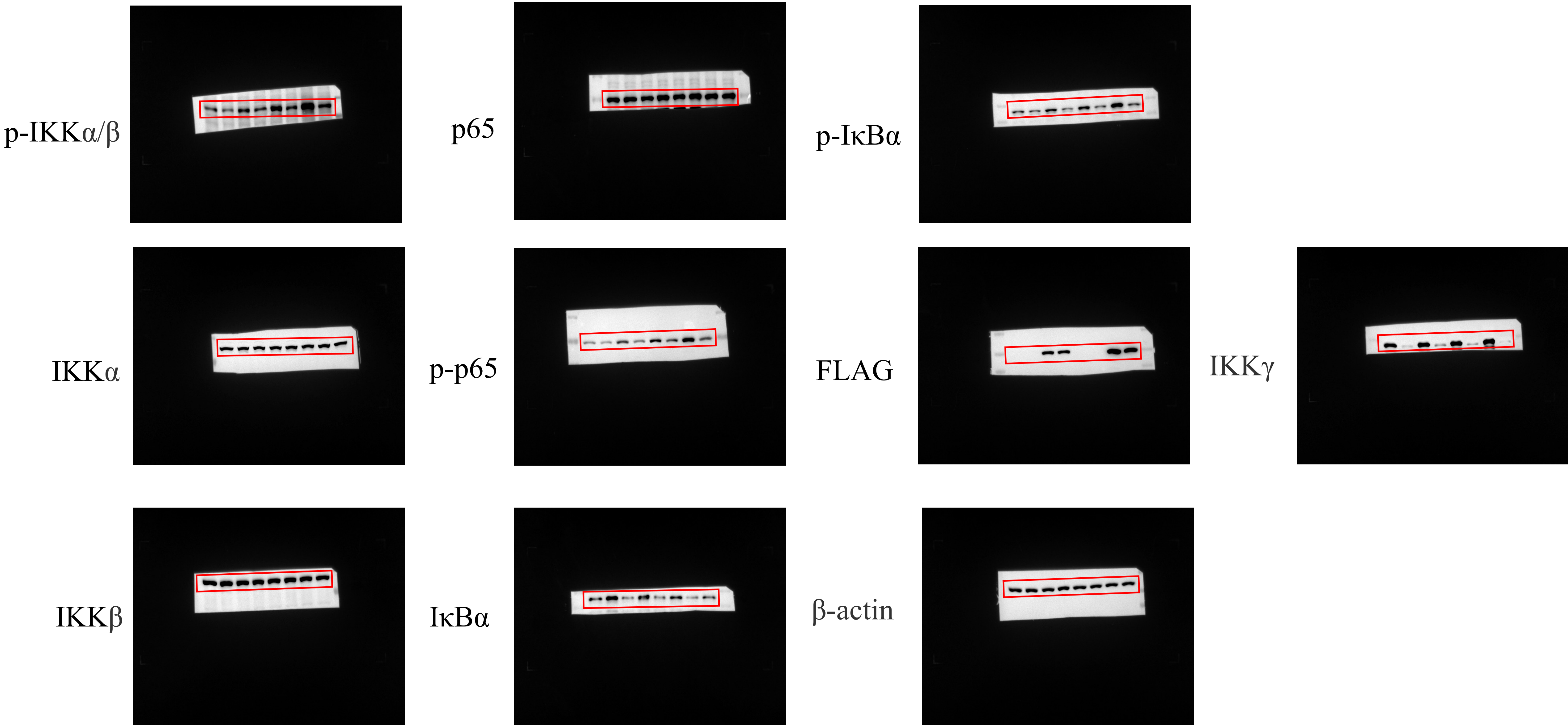

Supplementary Figure 4J

IKKγ

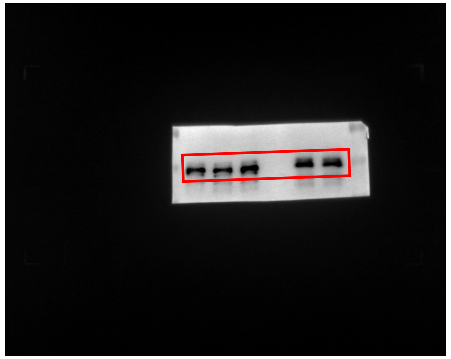

β-actin

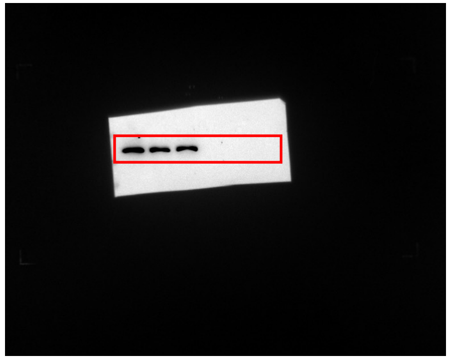

FLAG

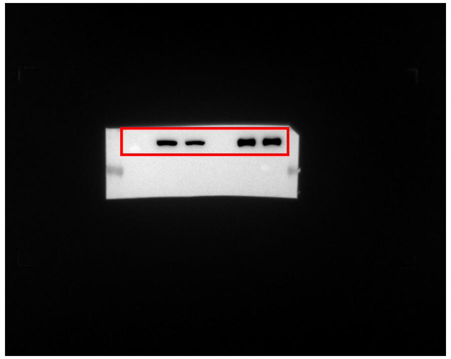

HA

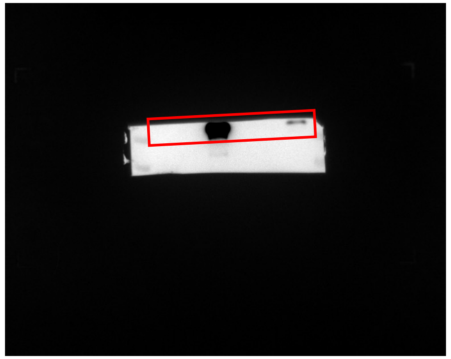

Supplementary Figure 4K

IKKγ

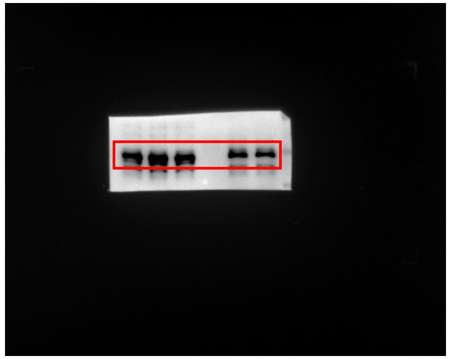

β-actin

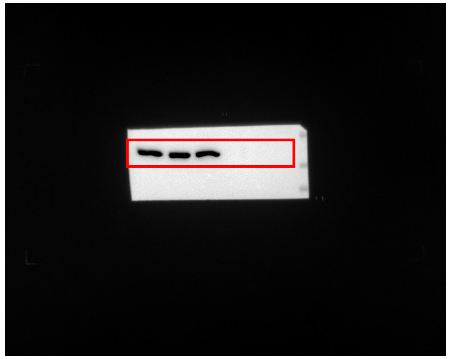

FLAG

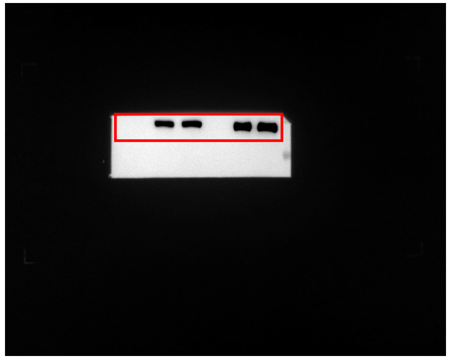

HA

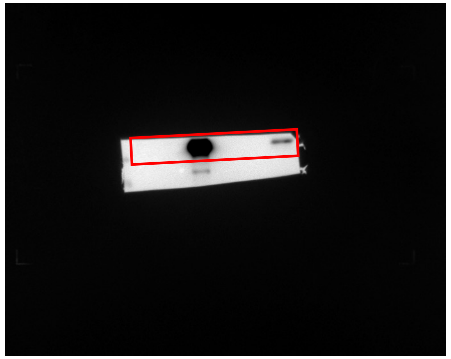

Supplementary Figure 4L

IKKγ

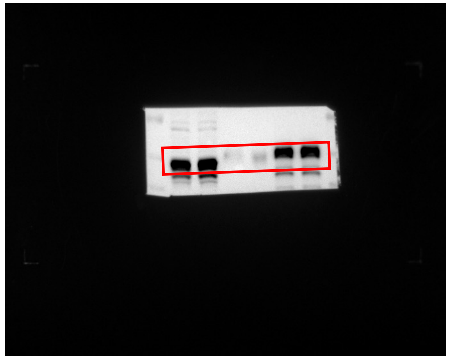

IKKα

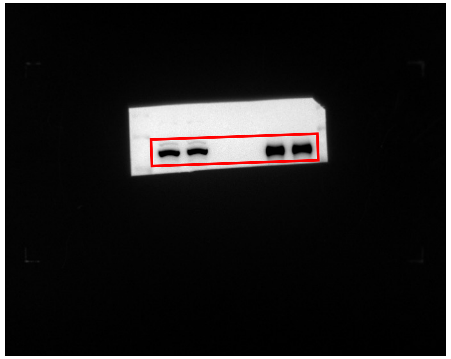

HHEX

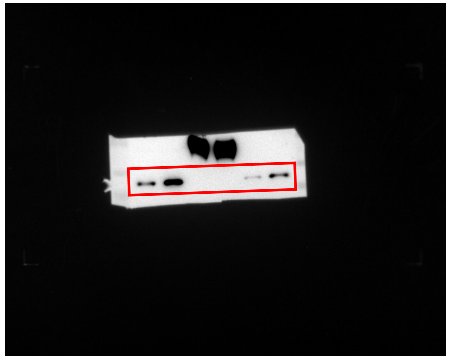

β-actin

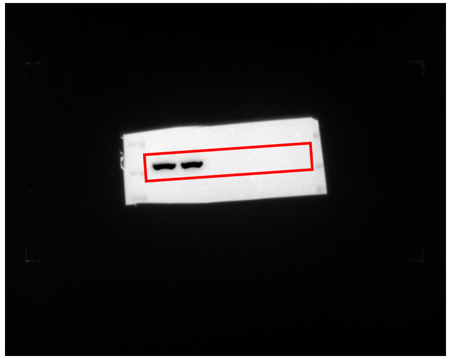

Supplementary Figure 4M

IKKγ

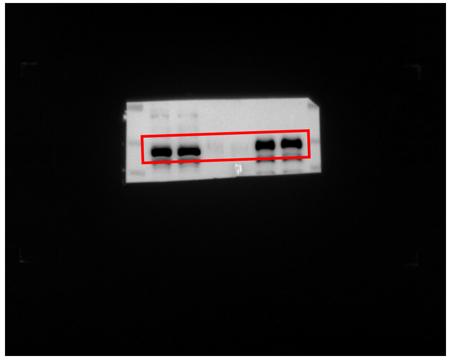

β-actin

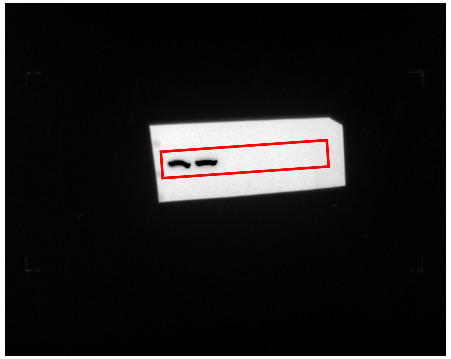

IKKα

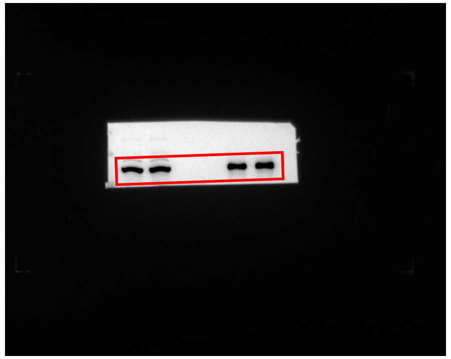

HHEX

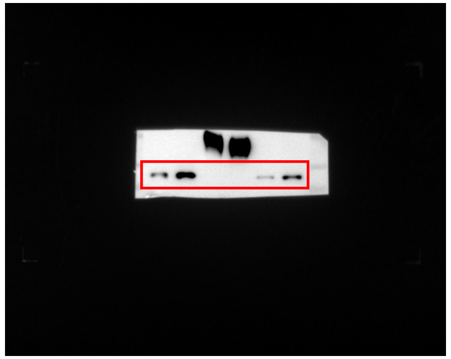

Supplementary Figure 5A

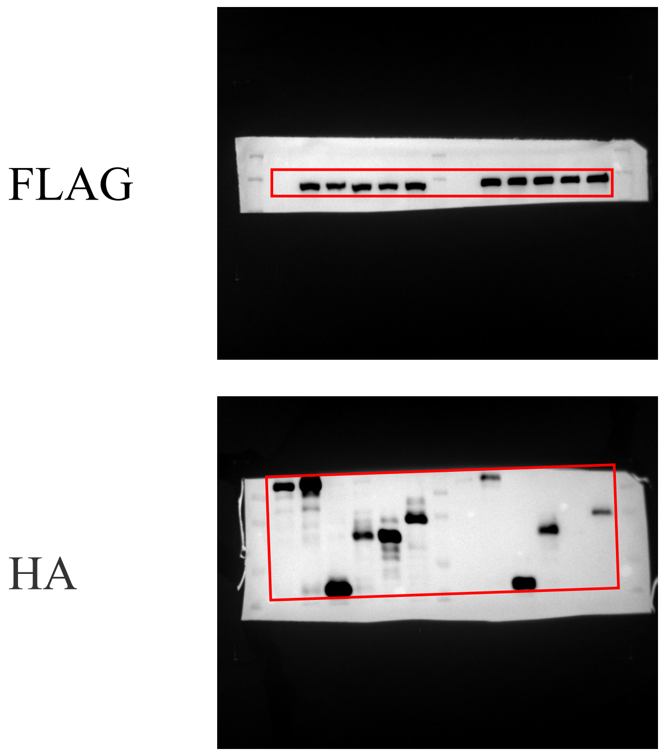

Supplementary Figure 5C

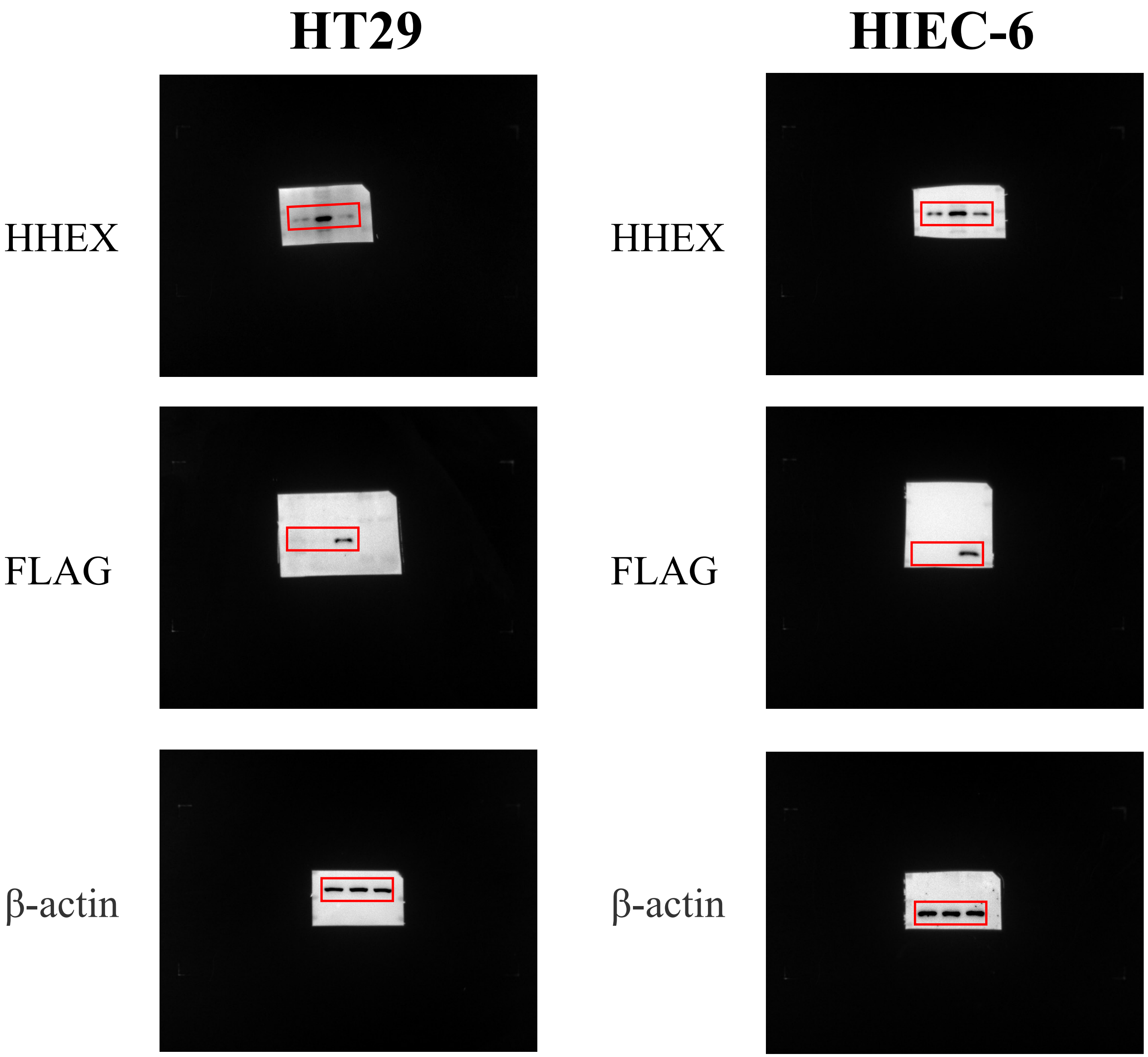

Supplementary Figure 5E HT29

IKKα

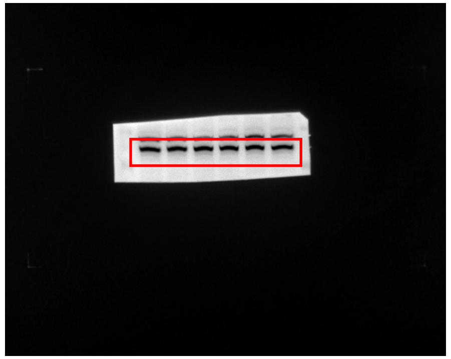

p-p65

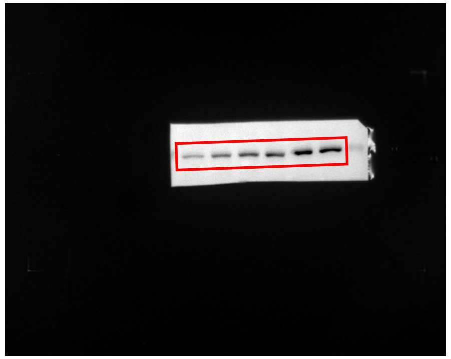

β-actin

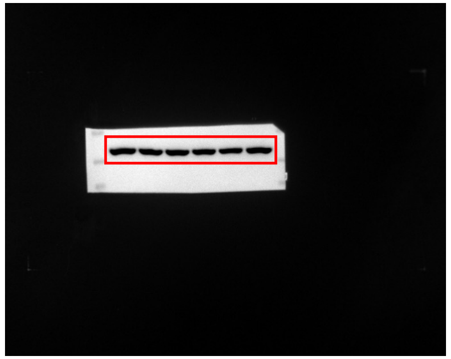

IKKβ

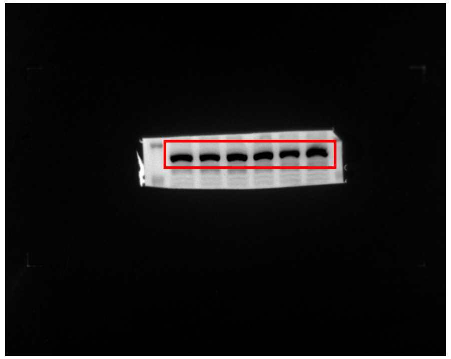

IκBα

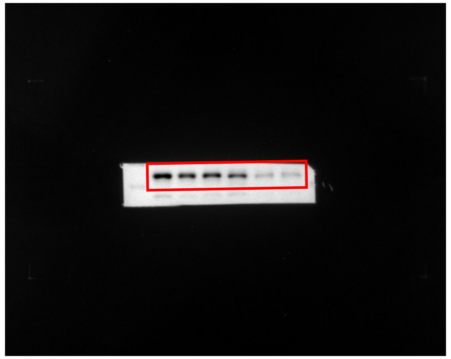

p65

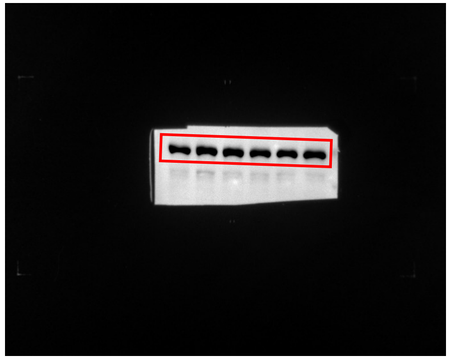

p-IκBα

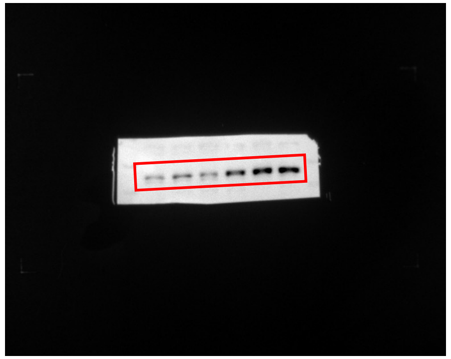

Supplementary Figure 5E HIEC-6

IKKα

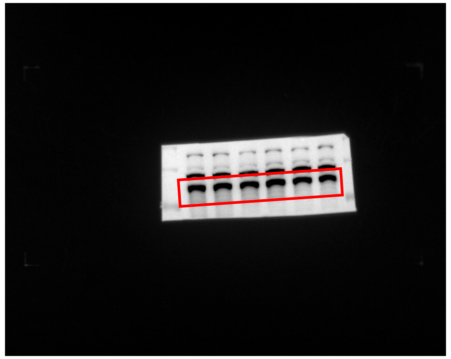

p-p65

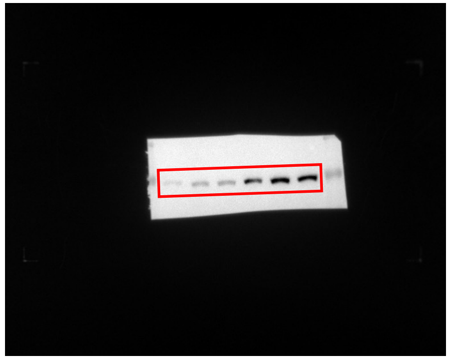

β-actin

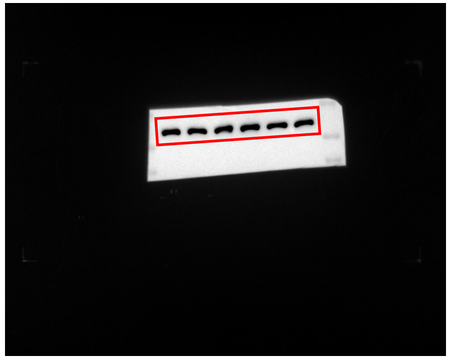

IKKβ

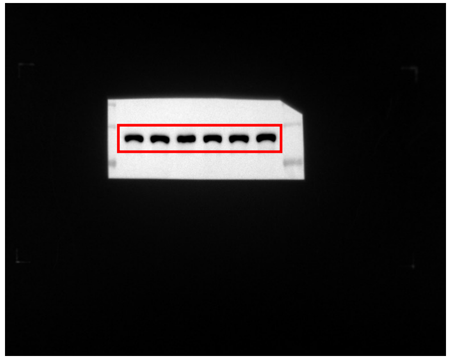

IκBα

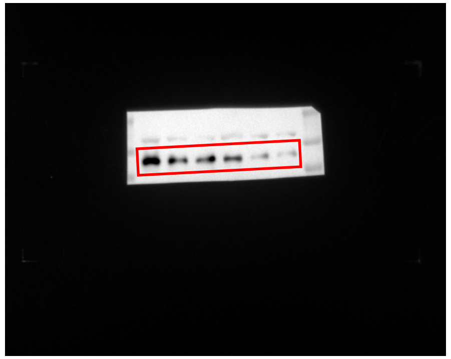

p65

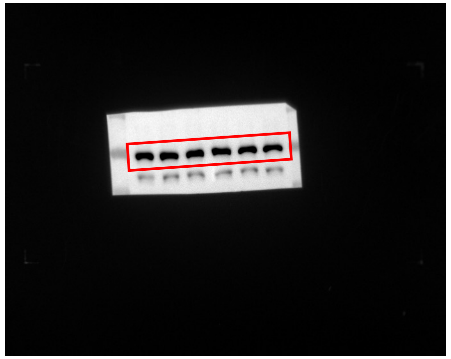

p-IκBα

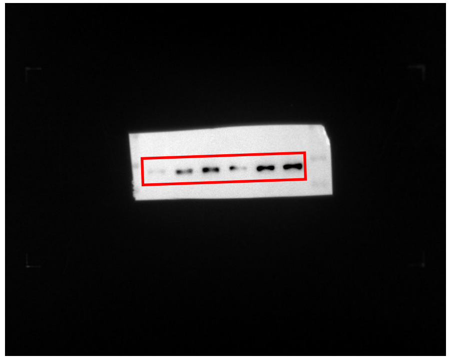

Supplementary Figure 5F

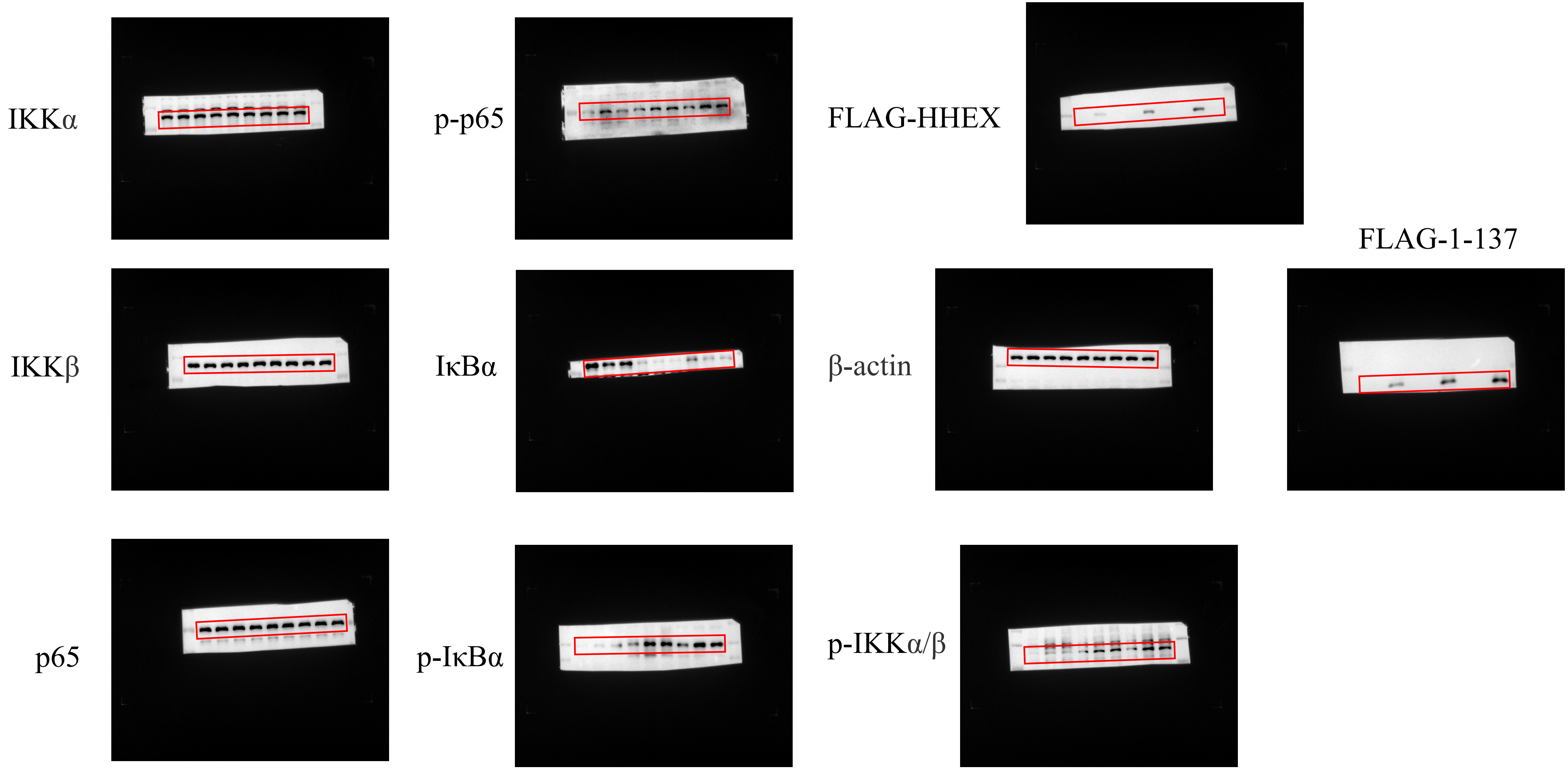

Supplementary Figure 5G LEFT

IKKβ

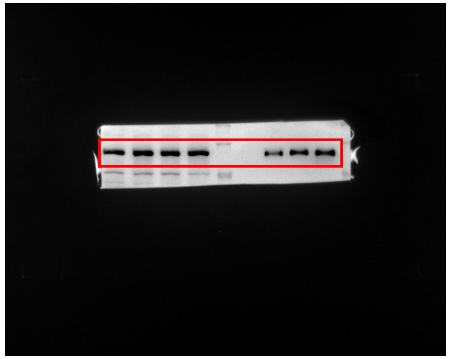

β-actin

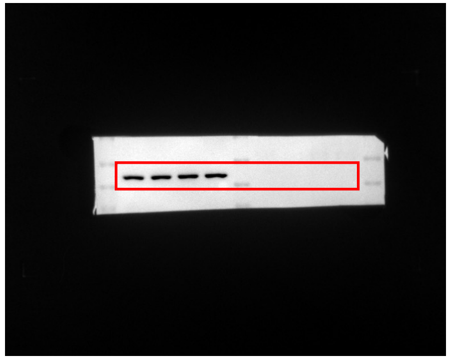

FLAG

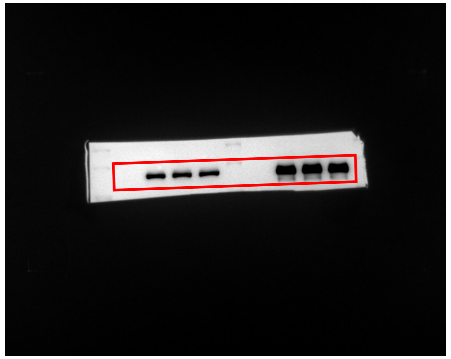

HA

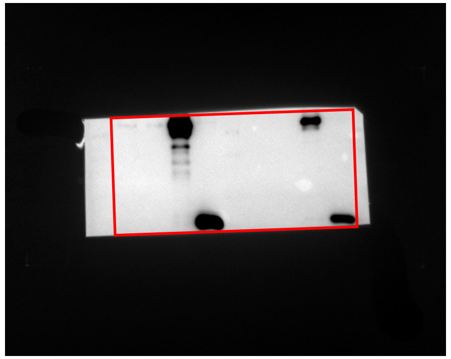

Supplementary Figure 5G RIGHT

IKKα

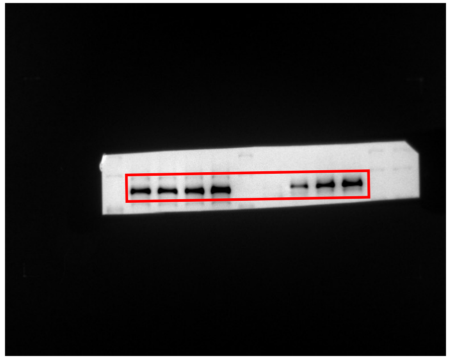

β-actin

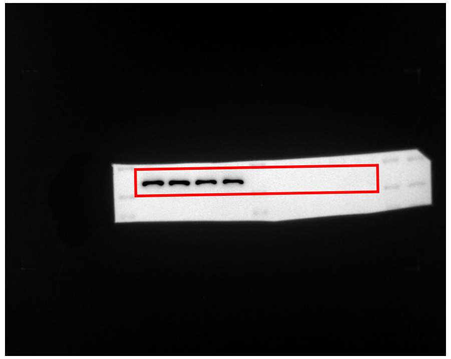

FLAG

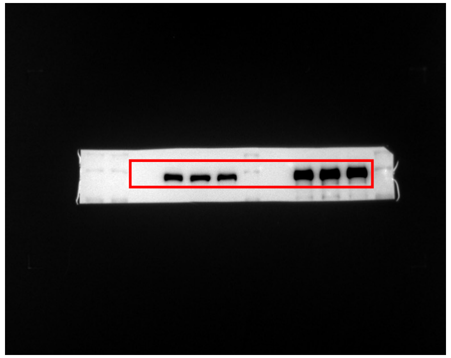

HA

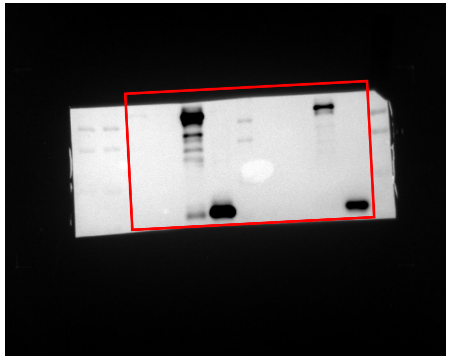

Supplementary Figure 5H

IKKβ

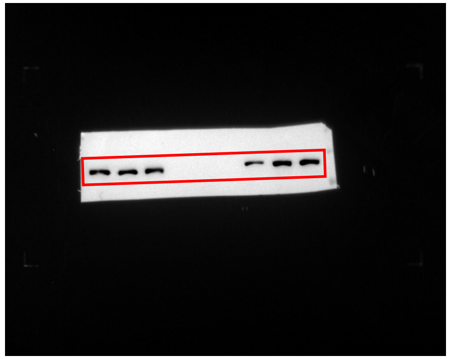

FLAG

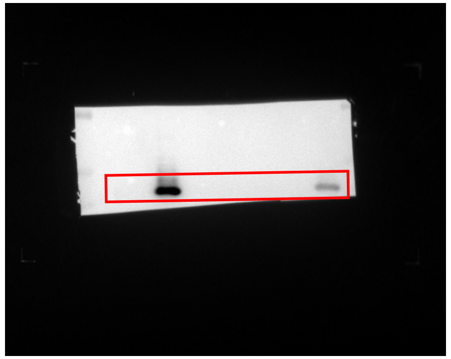

IKKα

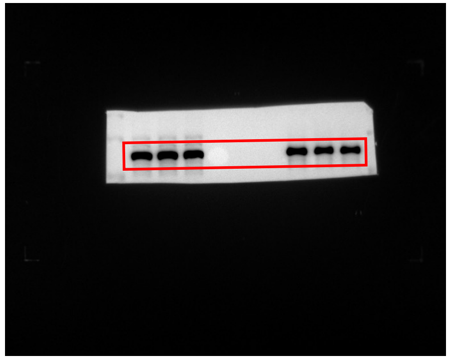

β-actin

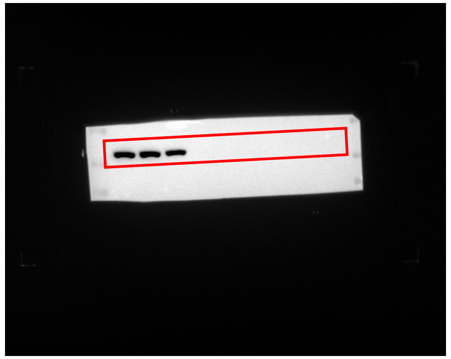

HHEX

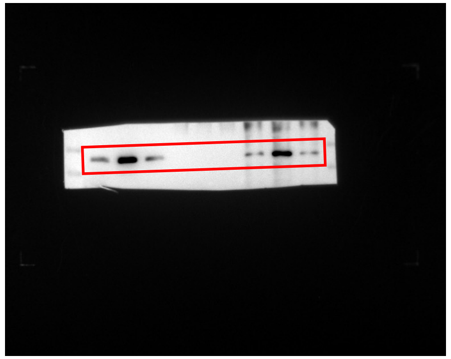

Supplementary Figure 6C

HHEX

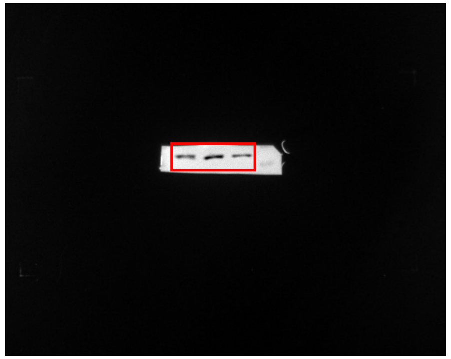

HA

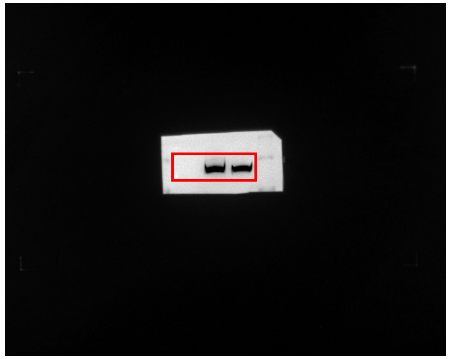

β-actin

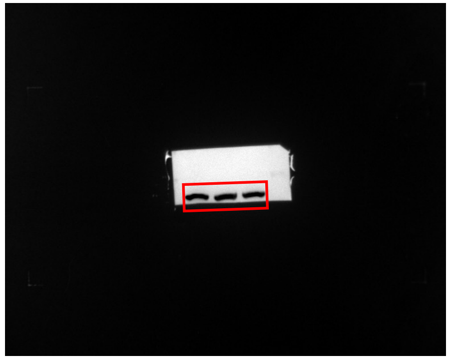

Supplementary Figure 6D

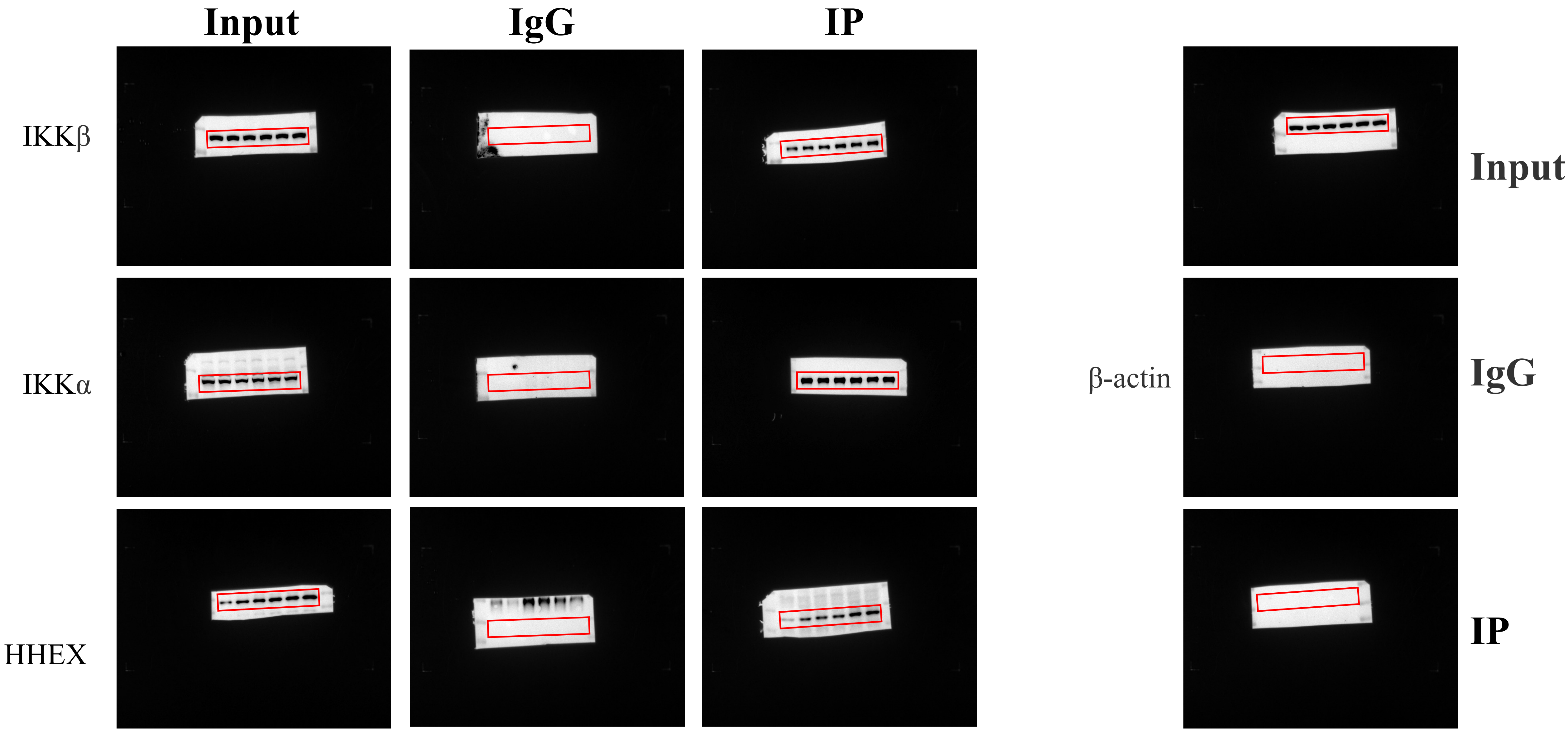

Supplementary Figure 6E

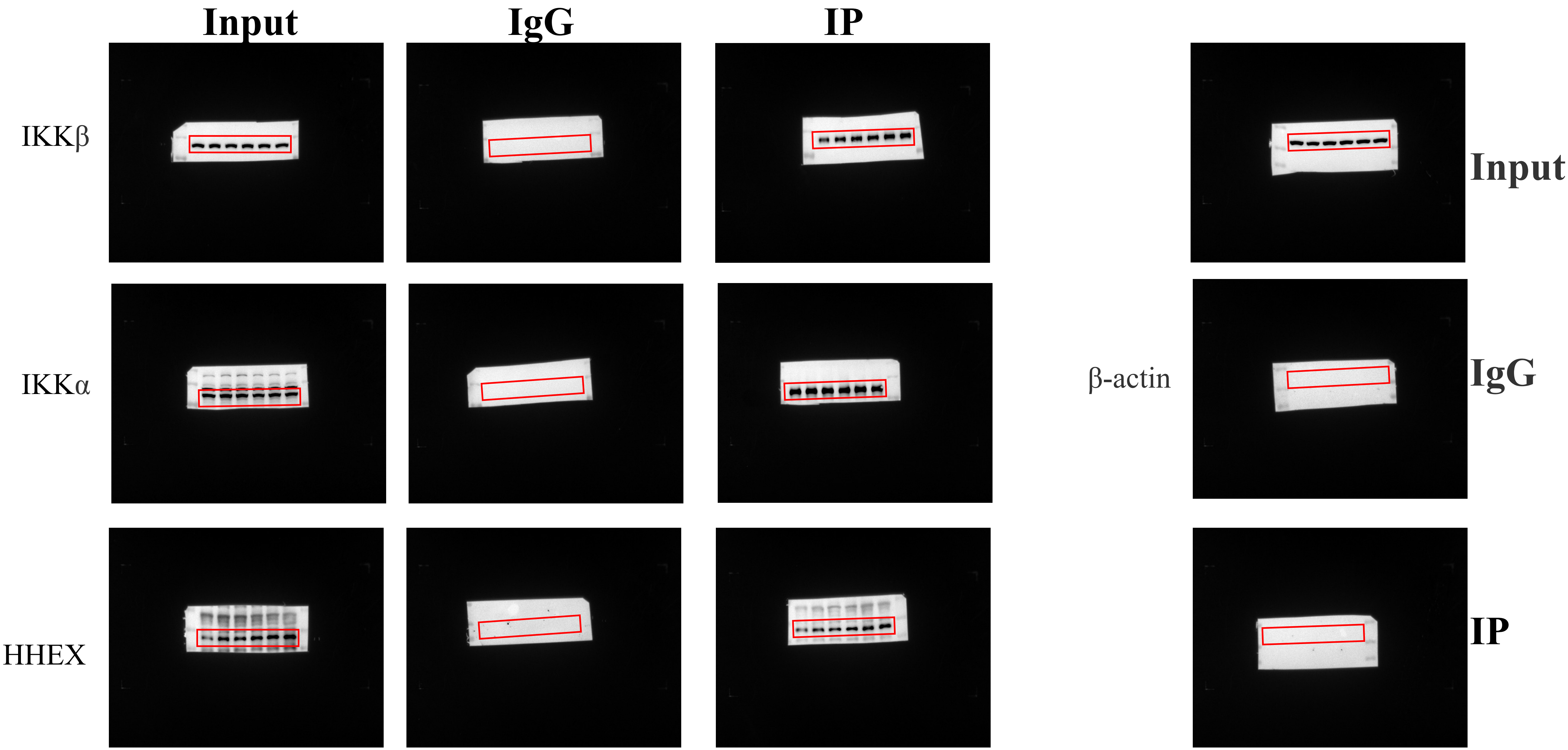

Supplementary Figure 7A

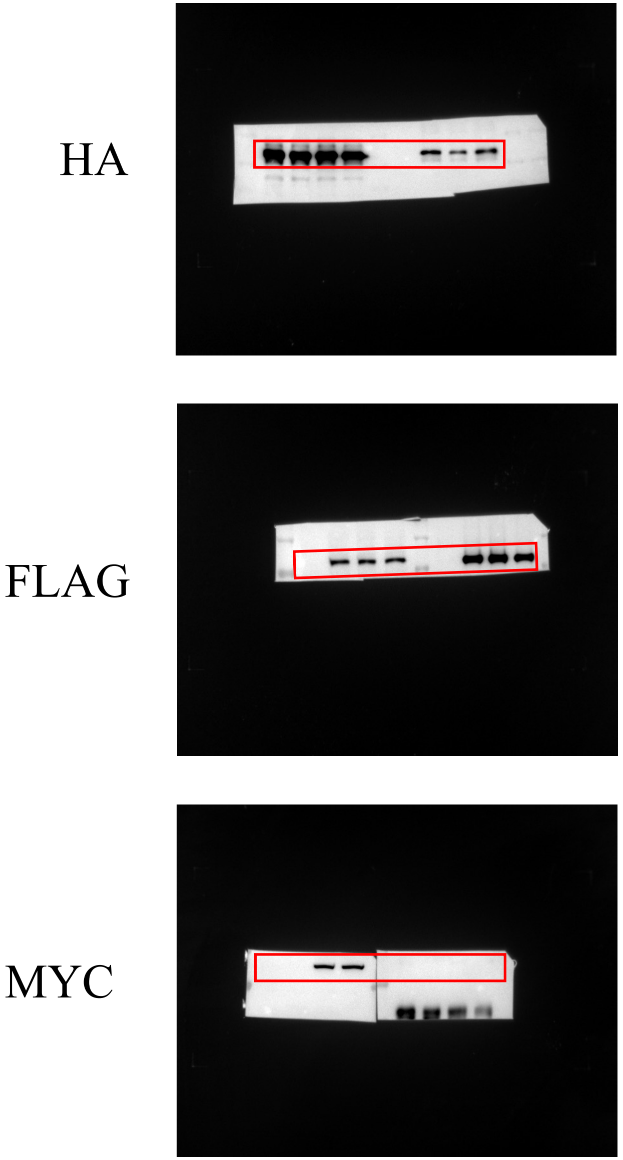

Supplementary Figure 7B

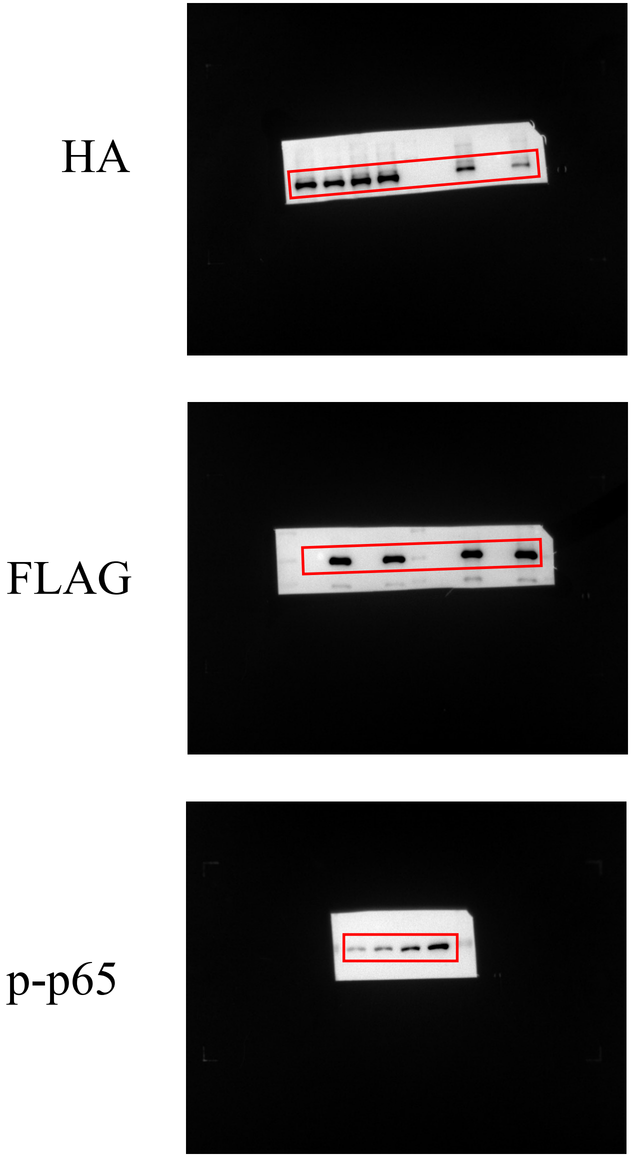

Supplementary Figure 7C

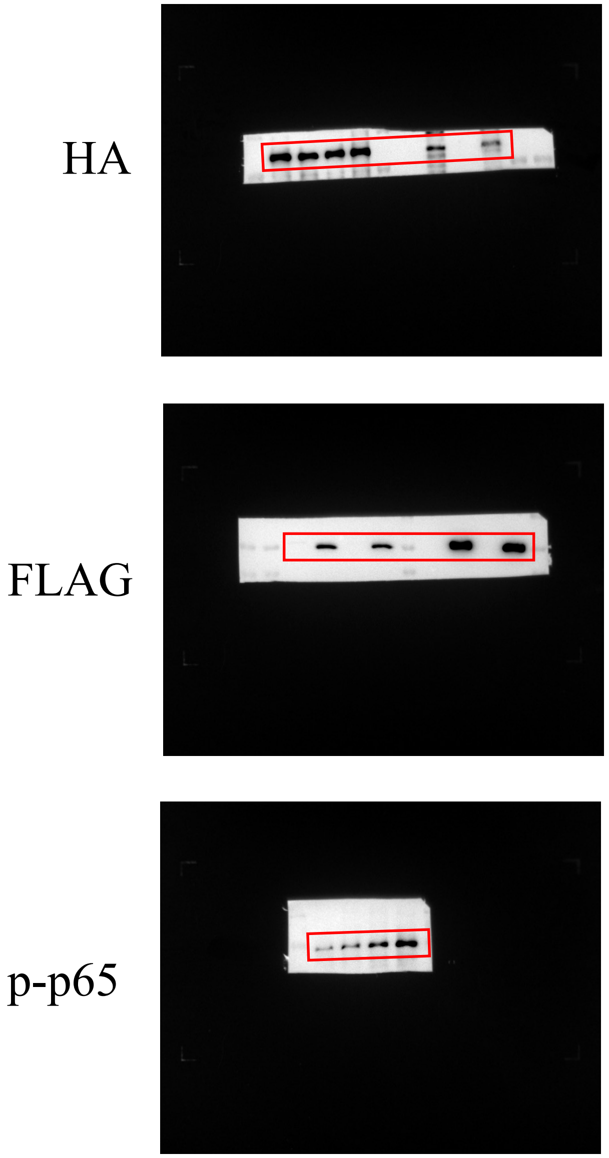

Supplementary Figure 7D

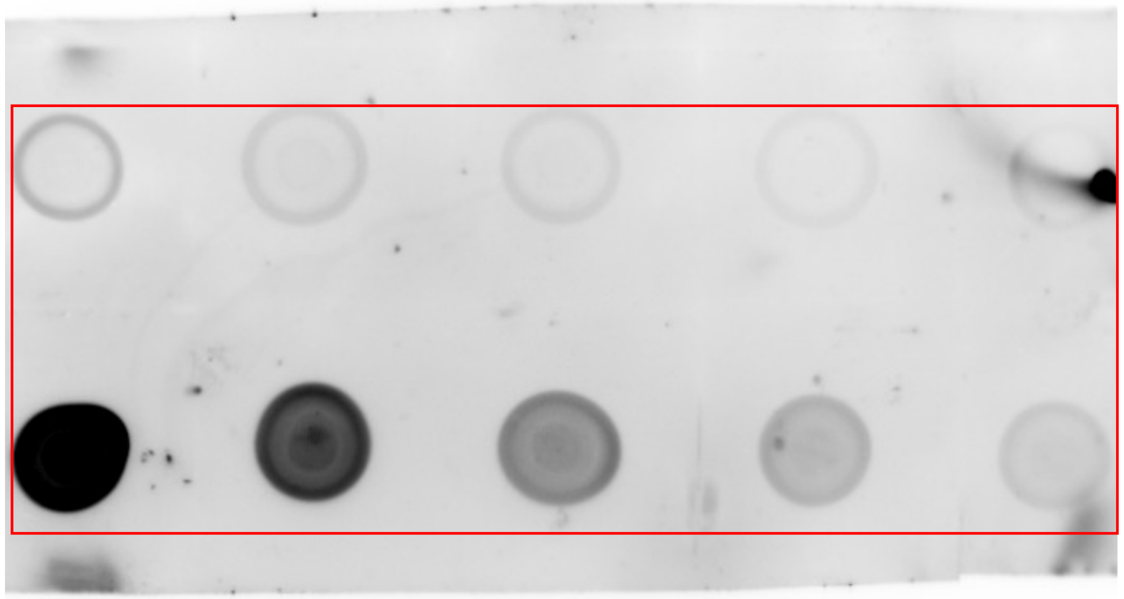

Supplementary Figure 7E

MID2

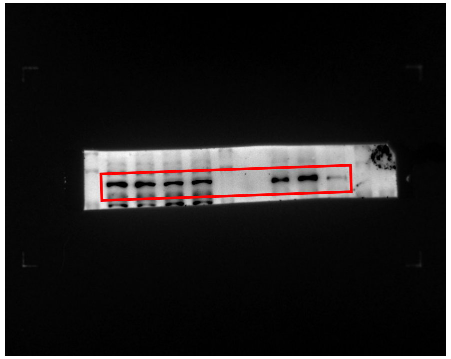

FLAG

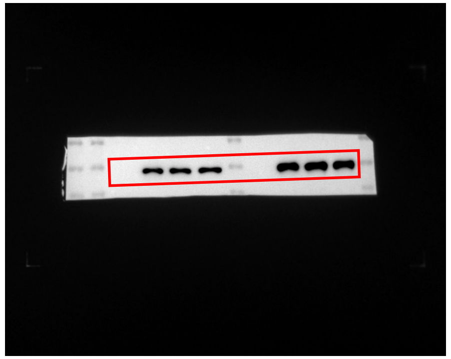

β-actin

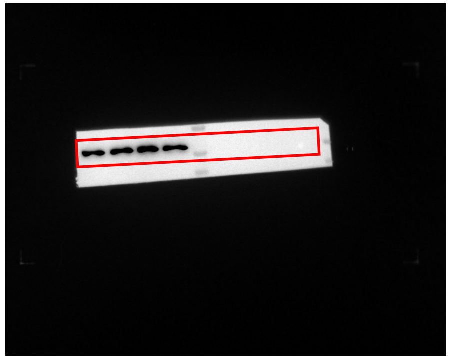

Supplementary Figure 7F

HA

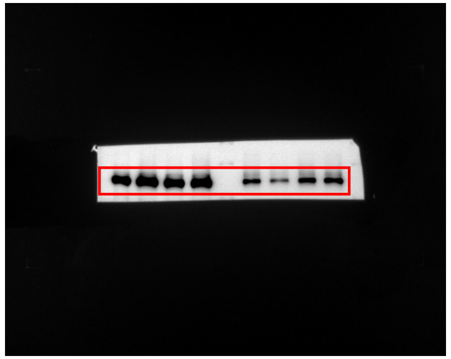

FLAG

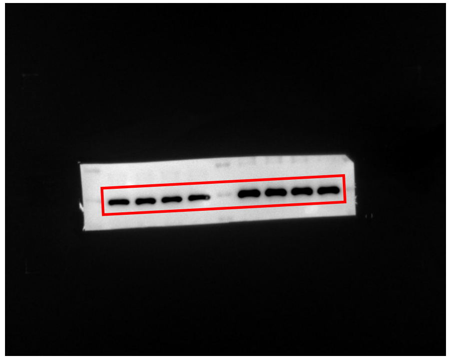

MYC

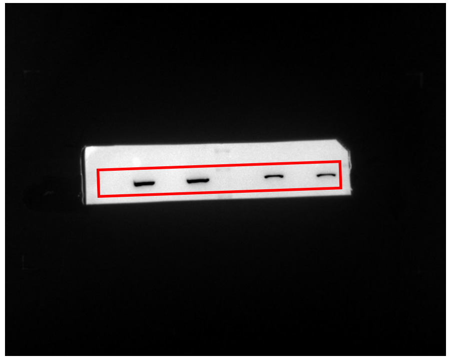

Supplementary Figure 7G

HA

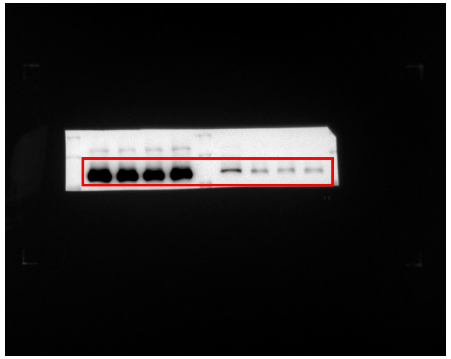

FLAG

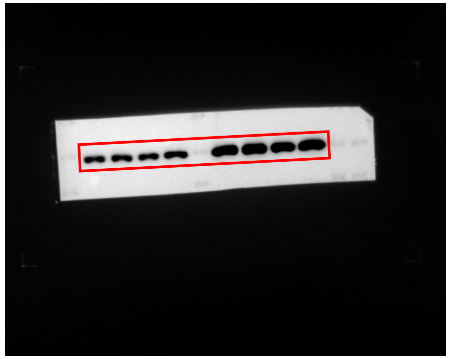

MYC

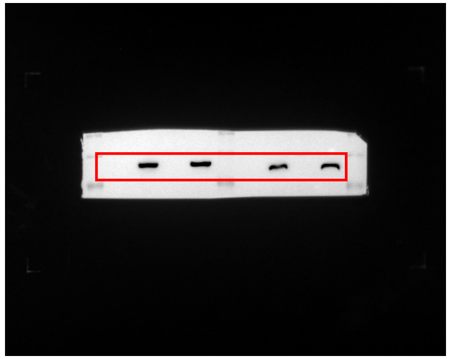

Supplementary Figure 7H

FLAG input

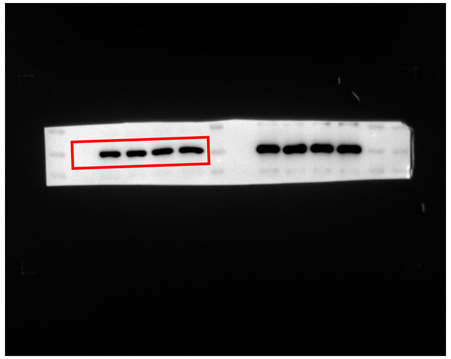

FLAG IP

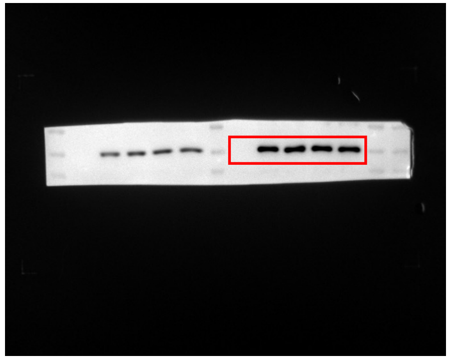

HA

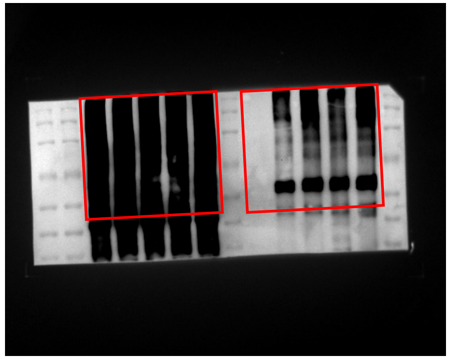

GFP

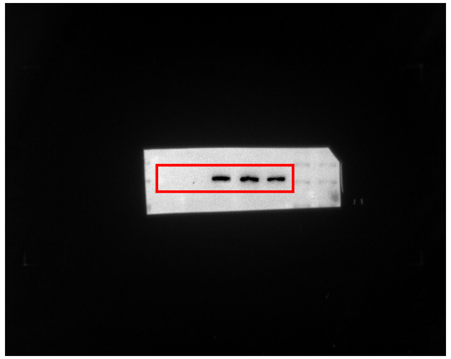

MYC

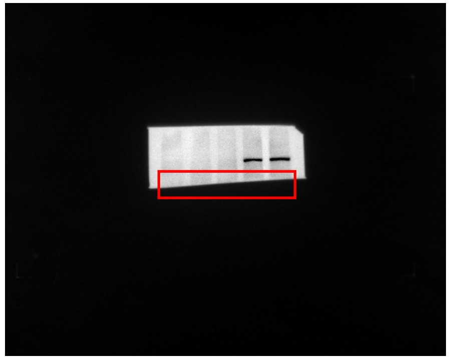

Supplementary Figure 7I

FLAG

HA input

HA IP

GFP

MYC

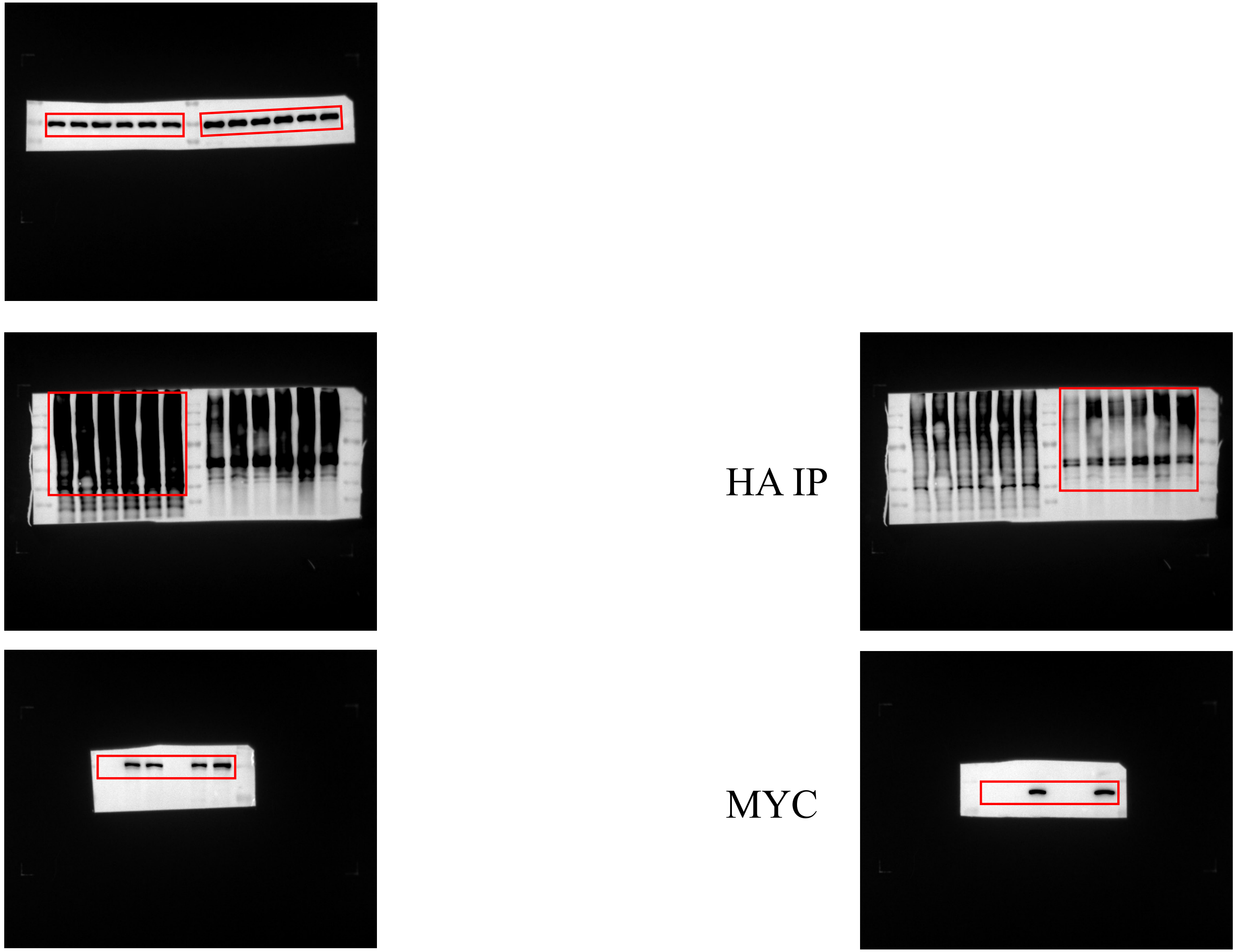

Supplementary Figure 7J

FLAG

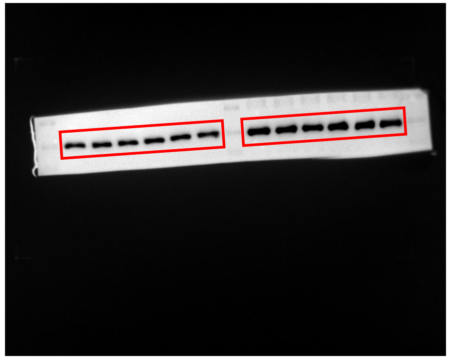

HA input

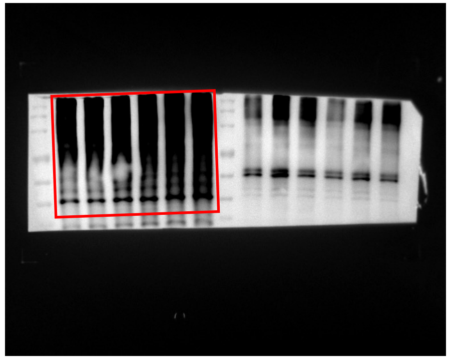

HA IP

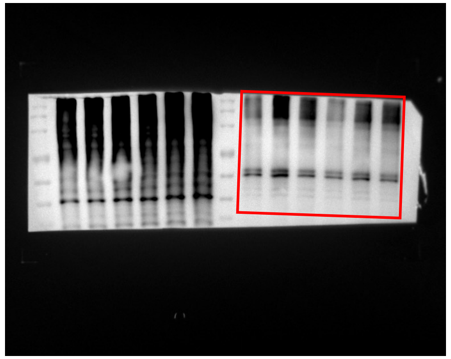

GFP

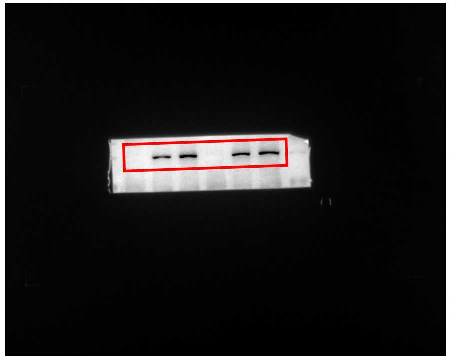

MYC

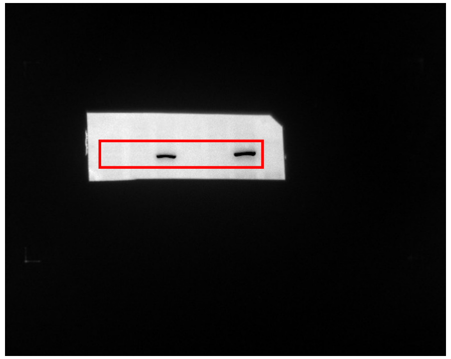

Supplementary Figure 7K

FLAG-HHEX

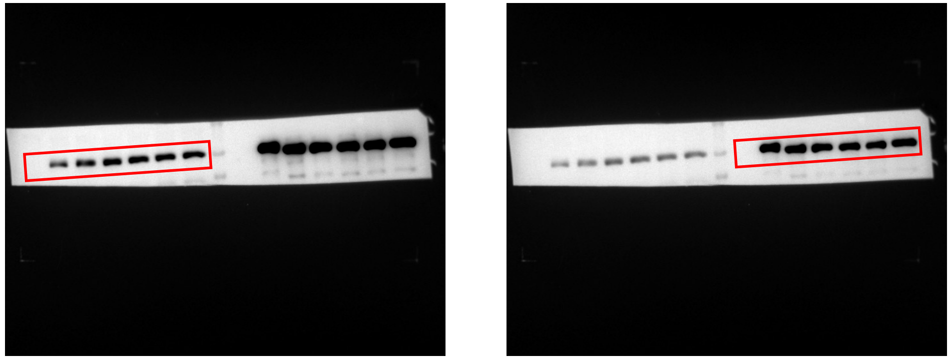

p-S213

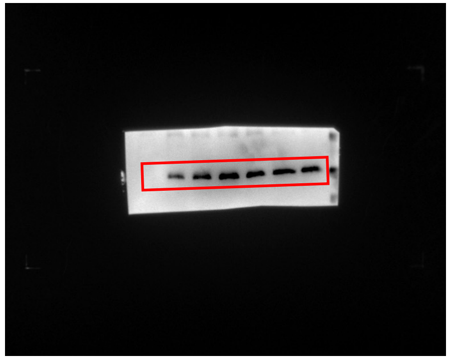

MID2

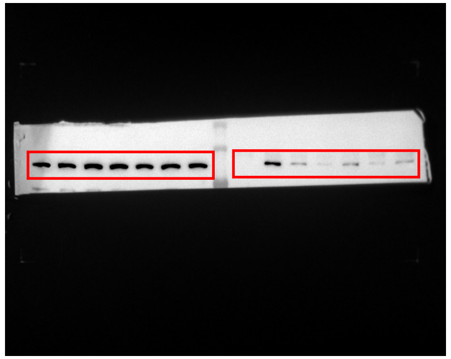

β-actin

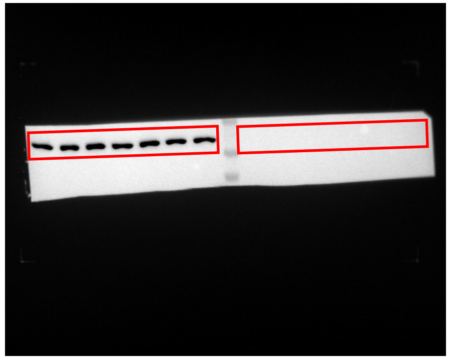

IKKα

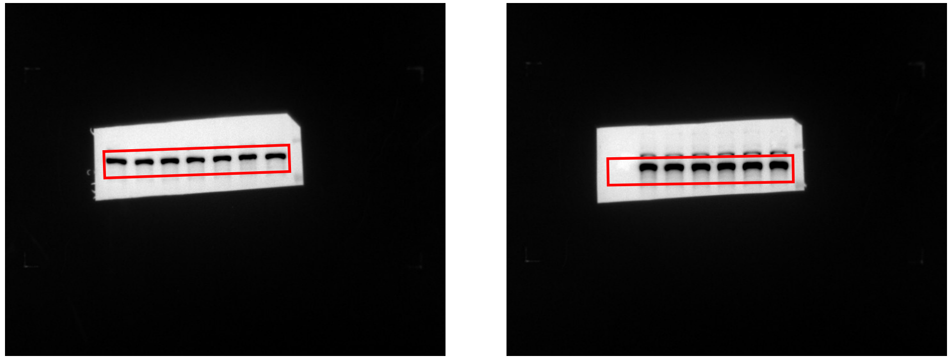

Supplementary Figure 8B

HHEX

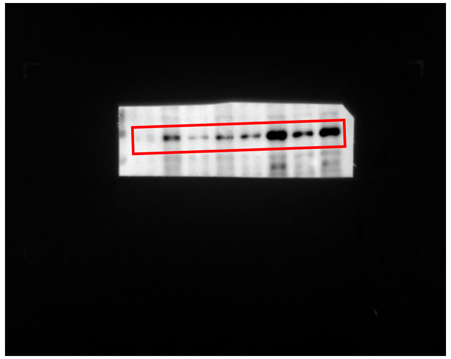

FLAG

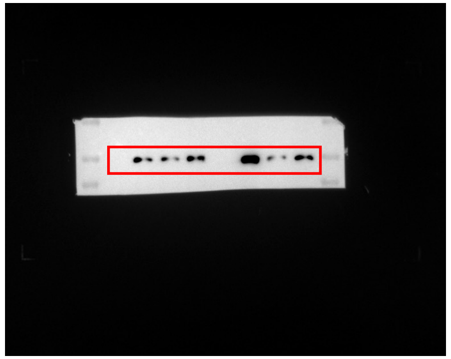

β-actin

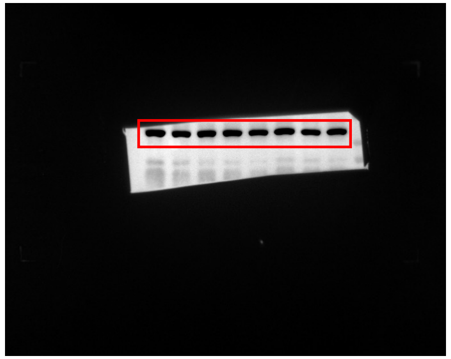

Supplementary Figure 8C

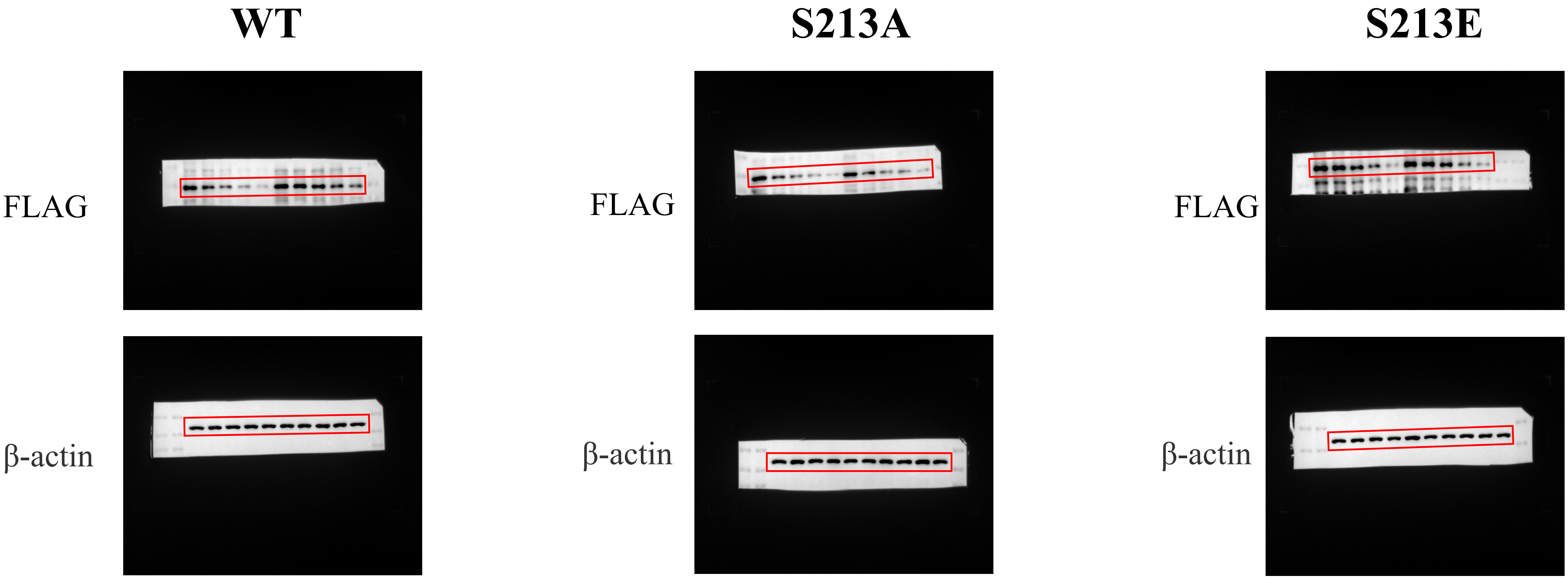

Supplementary Figure 9A

HHEX

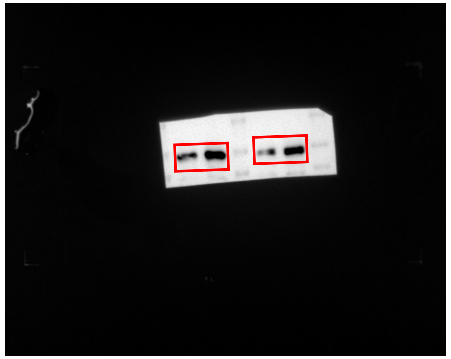

Lamin B1

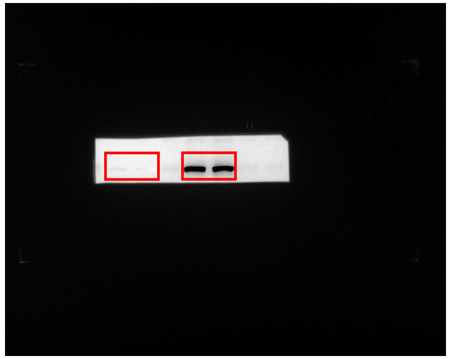

β-Tubulin

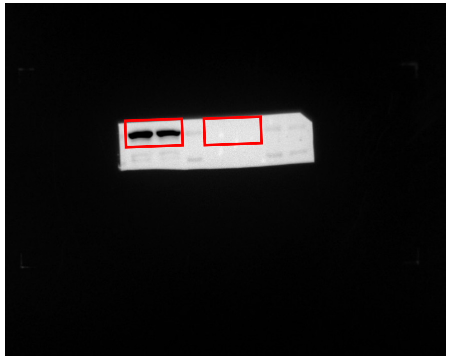

Supplementary Figure 9B HT29

Cytoplasmic Nuclear

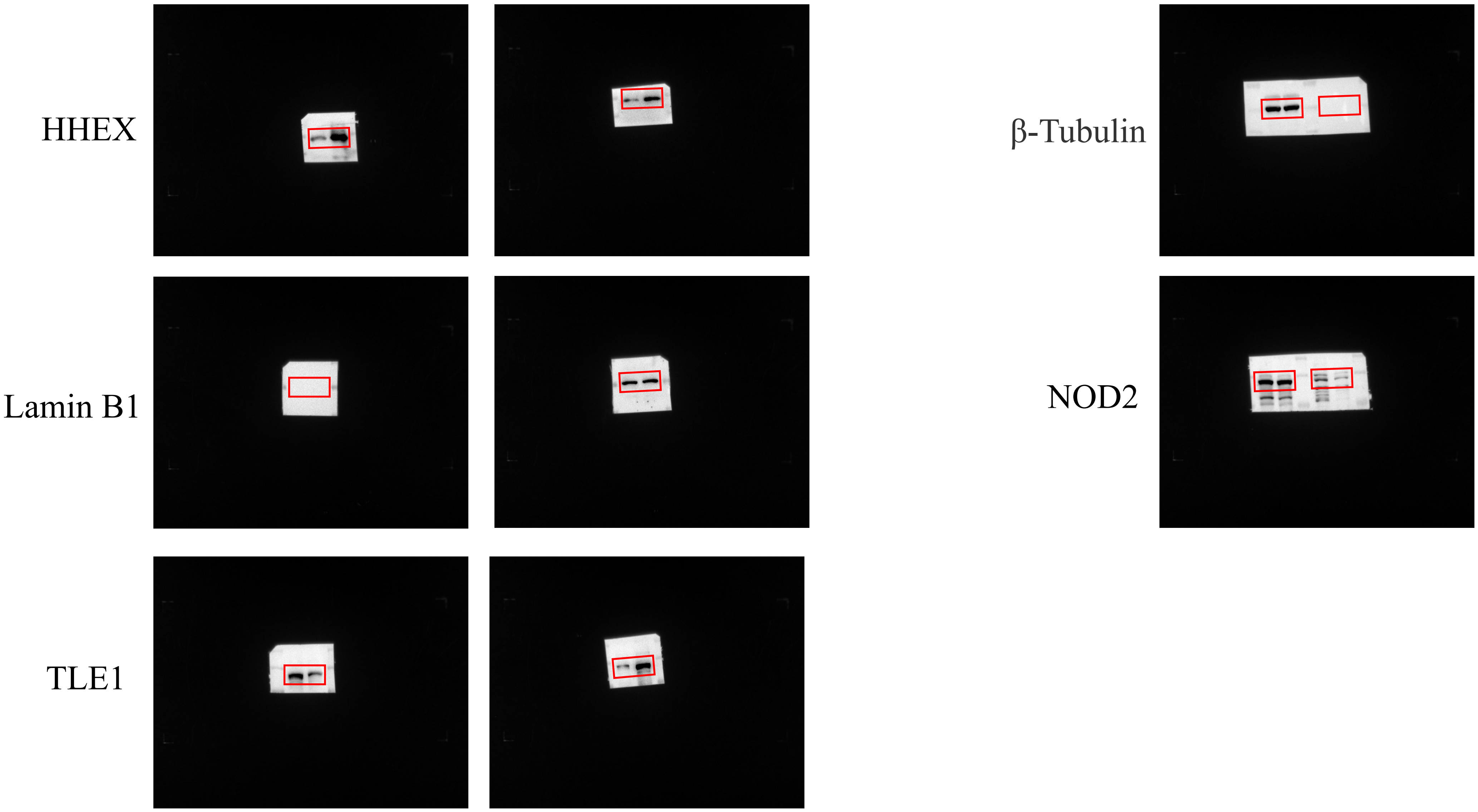

Supplementary Figure 9B HIEC-6

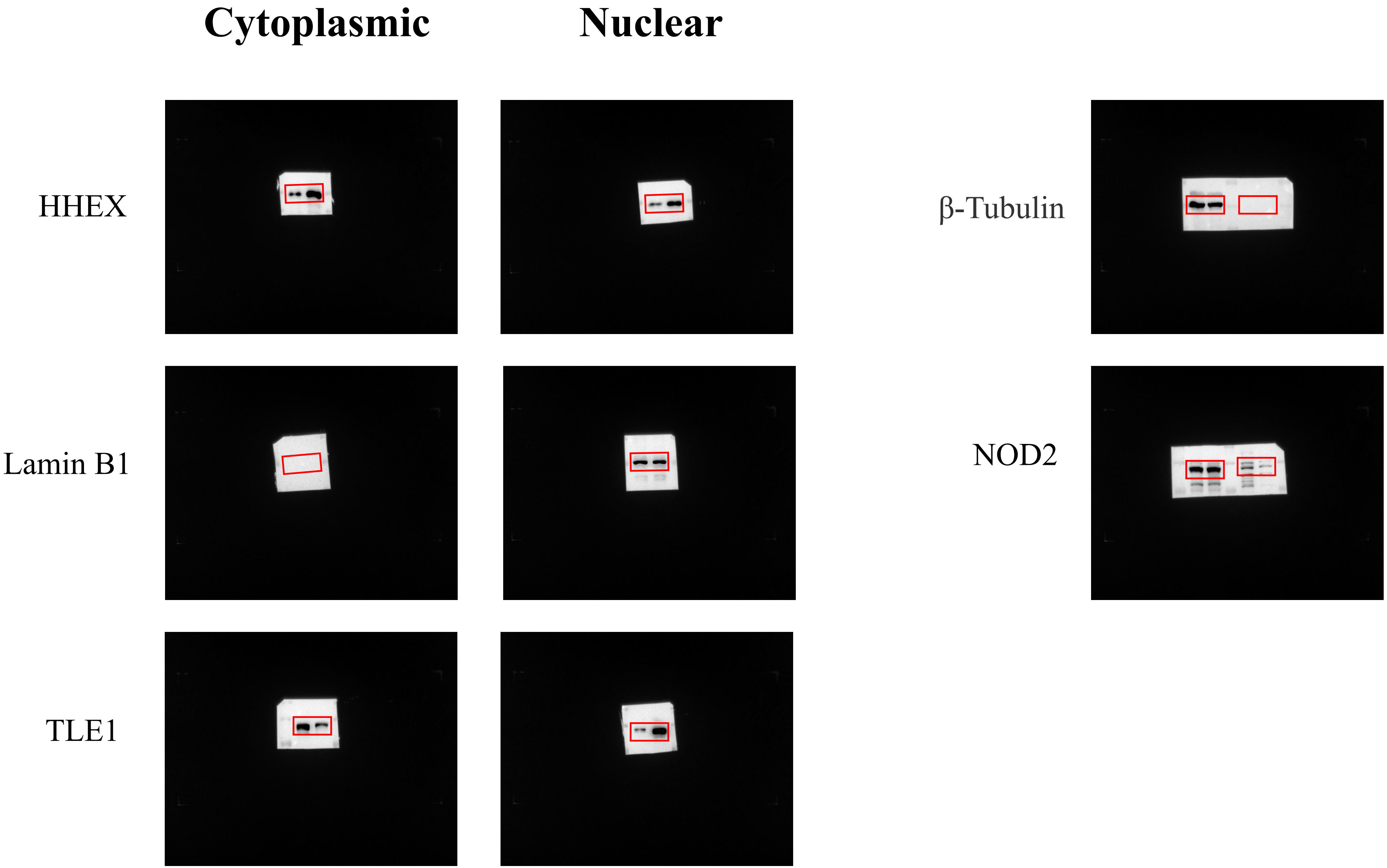

Supplementary Figure 9C

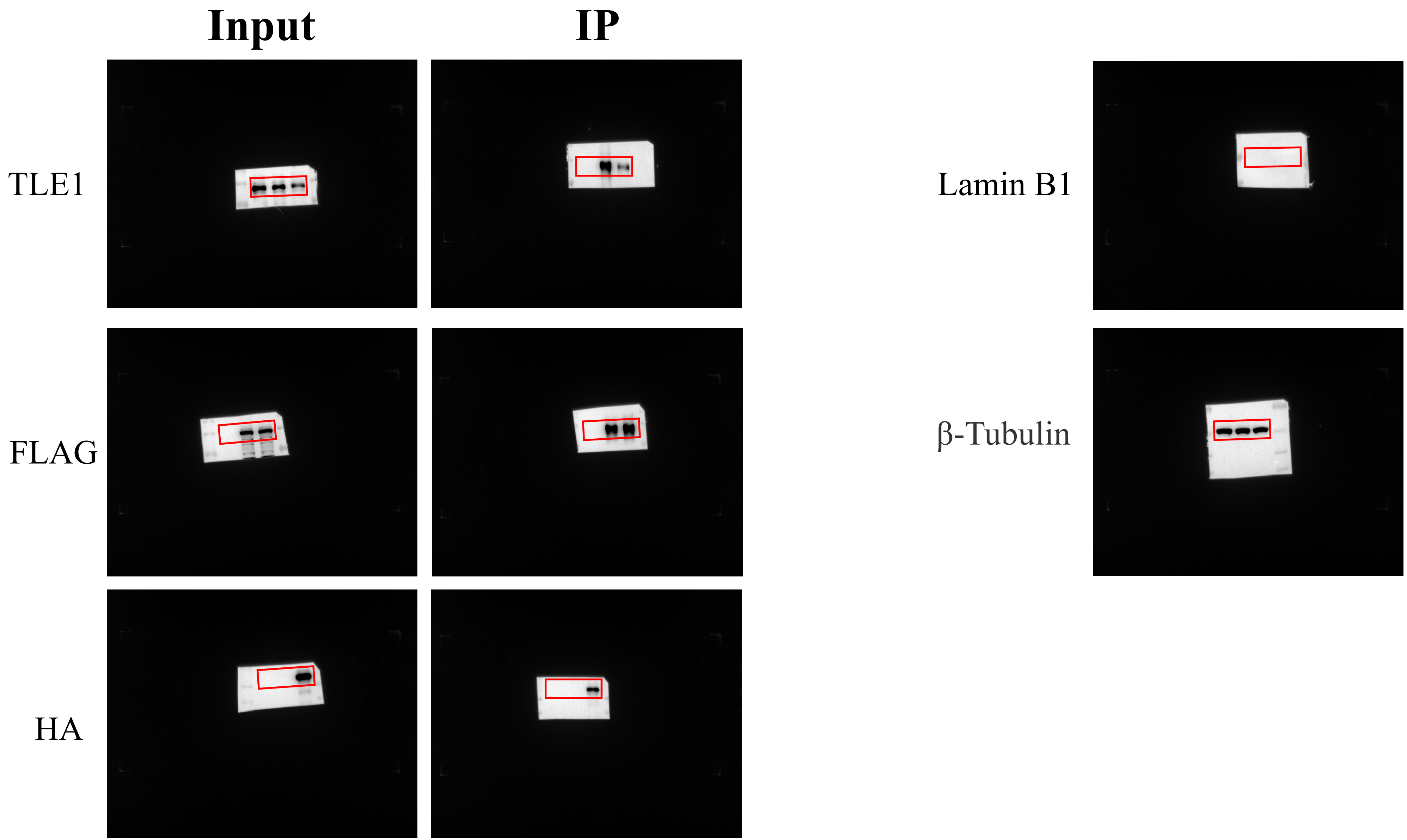

Supplementary Figure 9D

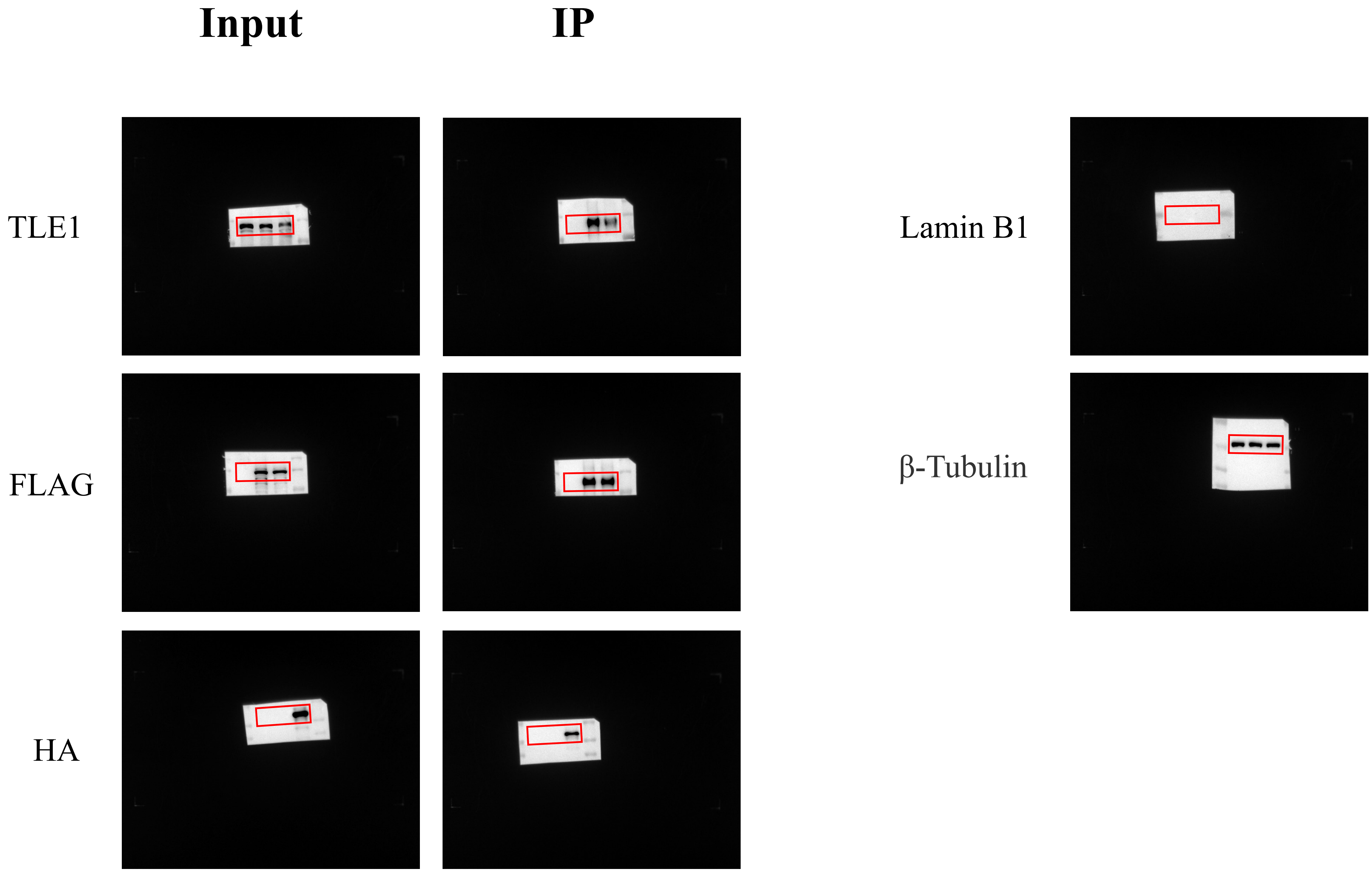

Supplement: Unedited blot and gel images [file jci-136-192074-s170.pdf]
